# Integrative analysis of proteomics and lipidomic profiles reveal the fat deposition and meat quality in Duroc × Guangdong small spotted pig

**DOI:** 10.3389/fvets.2024.1361441

**Published:** 2024-04-10

**Authors:** Zhuosui Wu, Zhonggang Wang, Pan Wang, Leiyan Cheng, Jianhao Li, Yanfeng Luo, Linfang Yang, Linfeng Li, Jianhua Zeng, Bin Hu

**Affiliations:** ^1^Guangdong Key Laboratory of Animal Breeding and Nutrition, Institute of Animal Science, Guangdong Academy of Agricultural Sciences, Guangzhou, China; ^2^Guangdong Guanghong Agriculture and Animal Husbandry Development Co, Ltd., Huizhou, China; ^3^Guangdong Yihao Foodstuff Co, Ltd., Guangzhou, China

**Keywords:** fat deposition, lipidomics, proteomics, metabolic pathways, Duroc × Guangdong small spotted pig

## Abstract

**Introduction:**

This study aims to explore the important factors affecting the characteristics of different parts of pork.

**Methods:**

Lipidomics and proteomics methods were used to analyze DAL (differential lipids) and DAPs (differential proteins) in five different parts (longissimus dorsi, belly meat, loin, forelegs and buttocks) of Duhua pig (Duroc × Guangdong small spotted pig), to identify potential pathways affecting meat quality, investigating fat deposition in pork and its lipid-protein interactions.

**Results:**

The results show that TG (triglyceride) is the lipid subclass with the highest proportion in muscle, and the pathway with the most significantly enriched lipids is GP. DAP clustered on several GO terms closely related to lipid metabolism and lipogenesis (lipid binding, lipid metabolism, lipid transport, and lipid regulation). In KEGG analysis, there are two main DAP aggregation pathways related to lipid metabolism, namely Fatty acid degradation and oxidative phosphorylation. In PPI analysis, we screened out 31 core proteins, among which NDUFA6, NDUFA9 and ACO2 are the most critical.

**Discussion:**

PC (phosphatidylcholine) is regulated by SNX5, THBS1, ANXA7, TPP1, CAVIN2, and VDAC2 in the phospholipid binding pathway. TG is regulated by AUH/HADH/ACADM/ACADL/HADHA in the lipid oxidation and lipid modification pathways. Potential biomarkers are rich in SFA, MUFA and PUFA respectively, the amounts of SFA, MUFA and PUFA in the lipid measurement results are consistent with the up- and down-regulation of potential biomarker lipids. This study clarified the differences in protein and lipid compositions in different parts of Duhua pigs and provided data support for revealing the interactions between pork lipids and proteins. These findings provide contributions to the study of intramuscular fat deposition in pork from a genetic and nutritional perspective.

## 1 Introduction

China is a major producer and consumer of pork. To obtain high-quality pork that meets today’s market demands, companies and researchers use existing genetic resources to improve and breed pigs. Fat deposition affects the eating quality characteristics of pork such as flavor, juiciness, and tenderness. Consumers show a clear preference for pork with high intramuscular fat (1). Parts with high intramuscular fat content, such as rib eye, pork belly, etc., usually have a higher market price than cuts with lower content.

As the market pursues high-quality pork, the use of genetic resources for improved breeding has become a key means to improve meat quality. In this context, Guangdong small-eared pigs, as the largest breed of local pigs in China, have a large base group and play an important role in meeting market demand. Products developed using Guangdong small-eared pigs are playing an important role in China’s local pork market, occupy a higher share.

Fat deposition directly affects the taste, juiciness, tenderness, and other quality characteristics of pork (2–4). Lipid is a general term for a variety of hydrophobic or amphipathic small molecules, including fatty acids, glycerolipids, glycerophospholipids, sphingolipids, Sterol lipids, glycolipids, pregnenolone lipids and polyethylene, etc. (5). Lipid substances are the main components of biological membrane structures and are also involved in physiological processes, signal transduction and energy reserves (6). Fatty acids exist as molecular structures or independently in cells (7). They can be divided into saturated and unsaturated fatty acids. They have a variety of functions and make important contributions to various aspects such as meat quality, nutritional value and human health (8). They are related to a variety of Disease-related (9–14), among which polyunsaturated fatty acids play a key role in multiple physiological processes (15–18). Protein is one of the main components of pork and affects quality characteristics such as taste and tenderness. The human body needs to obtain some essential amino acids that cannot be synthesized from the diet (19). Animals can convert low-quality proteins and store these nutrients, so that humans can obtain nutrients (20). Meat is an important source of human protein, and pork protein is excellent source of animal protein. Lipidomic systematically studies change in lipid composition and expression of organisms through high-throughput analysis technology, which is crucial to elucidating related biological activity processes and mechanisms. The application of proteomics and mass spectrometry technology reveals the molecular mechanisms behind meat quality and provides data support for exploring the regulatory network between pork lipids and proteins. This study comprehensively applied lipidomic and proteomics technologies to systematically study the changes in lipids and proteins in different parts of pork and look for potential biomarkers related to meat quality. The regulatory network between lipids and proteins provides important clues and contributes to the study of intramuscular fat deposition in pork from a genetic and nutritional perspective.

## 2 Materials and methods

### 2.1 Animals and samples

**Figure 1:**
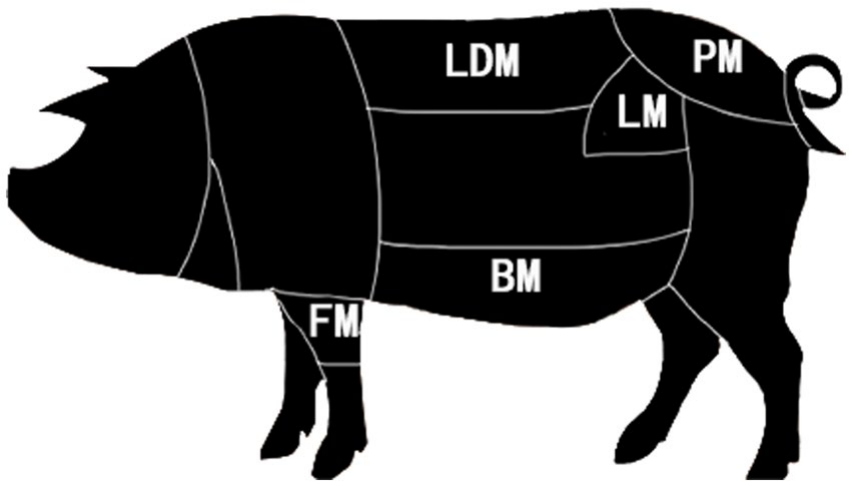
Pork segmentation.

Duhua crossbred pigs from the same pig farm fed the same commercial feed and fattened were selected. After reaching the market weight, 8 pigs were selected, they were fasted for 12 h and slaughtered in the local slaughterhouse according to conventional slaughterhouse procedures. After slaughter, the longissimus dorsi muscle, belly meat, loin, front leg meat, and buttock meat were sampled after removing the visible fat and connective tissue, placed in liquid nitrogen, and stored at-80°C for omics analysis immediately (Figure 1).

### 2.2 Lipidomic detection

1. Lipids extraction: Take an appropriate amount of sample, add 200 μL water, MP vortex, add 800 μL MTBE, vortex mix, add 240 μL pre-cooled methanol, vortex mix, ultrasonicate in a low-temperature water bath for 20 min, and leave at room temperature for 30 min, centrifuge at 14000 g and 10°C for 15 min, take the upper organic phase, blow dry with nitrogen, add 200 μL of 90% isopropyl alcohol/acetonitrile solution to reconstitute during mass spectrometry analysis, vortex thoroughly, take 90 μL of the reconstituted solution, and centrifuge at 14000 g and 10°C for 15 min. Min, take the supernatant and inject it for analysis.
2. Chromatographic conditions: The samples were separated using UHPLC Nexera LC-30A ultra-high performance liquid chromatography system. C18 chromatographic column; column temperature 45°C; flow rate 300 μL/min. Mobile phase composition A: acetonitrile aqueous solution (acetonitrile: water = 6:4, v/v) + 0.1% formic acid +0.1 mM ammonium formate, B: acetonitrile isopropanol solution (acetonitrile: isopropanol = 1:9, v/v) +0.1% formic acid +0.1 mM ammonium formate. The gradient elution program is as follows: 0–2 min, B is maintained at 30%; 2–25 min, B changes linearly from 30 to 100%; 25-35 min, B is maintained at 30%. The samples were placed in the autosampler at 10°C during the entire analysis process. In order to avoid the impact caused by instrument detection signal fluctuations, random order is used for continuous analysis of samples.
3. Mass spectrometry conditions: Electrospray ionization (ESI) positive ion and negative ion modes were used for detection, respectively. After the samples were separated by UHPLC, mass spectrometry analysis was performed using a Q Exactive series mass spectrometer (Thermo Scientific™). ESI source conditions are as follows: Heater Temp 300°C, Sheath Gas Flow rate 45 arb, Aux Gas Flow Rate15 arb, Sweep Gas Flow Rate 1arb, spray voltage 3.0KV, Capillary Temp 350°C, S-Lens RF Level 50%, MS 1 scan ranges: 200–1800 The mass-to-charge ratio of lipid molecules and lipid fragments is collected according to the following method: 10 fragment spectra (MS 2 scan, HCD) are collected after each full scan. MS 1 has a resolution of 70,000 at M/Z 200 and MS 2 has a resolution of 17,500 at M/Z 200.
4. Data analysis: Use LipidSearch to perform peak identification, peak extraction, lipid identification (secondary identification) and other processing on lipid molecules and internal standard lipid molecules. The main parameters are precursor tolerance: 5 ppm, product tolerance: 5 ppm, product ion threshold: 5%. The data extracted by LipidSearch are first evaluated for quality, and then analyzed for identification quantity statistics, lipid composition analysis, lipid difference analysis and other data analysis.

### 2.3 Free fatty acid detection

1. Metabolite extraction: After the sample is slowly thawed at 4°C, take 25 mg of the sample and add 5 mL of dichloromethane-methanol solution (2:1 v/v), vortex to mix, and add 2 mL of gold-labeled water. Wash, remove the layer solution, and blow dry with nitrogen. Add 2 mL of n-hexane, add internal standard, methyl esterify for 0.5 h, add 2 mL of gold-labeled water, absorb 2000 μL of the supernatant, blow dry with nitrogen, add n-hexane to reconstitute, and take the supernatant. Add the solution to the injection bottle and enter the GC–MS for detection. The injection volume is 1 μL, the split ratio is 10:1, and the sample is split.
2. Chromatographic conditions: The sample was separated using a capillary column (Agilent 19091S–433UI: HP-5 ms, 30 mx 250 μm × 0.25 μm) gas chromatography system. Programmed temperature rise: initial temperature 80°C; increase temperature to 180°C at 20°C/min and maintain for 8 min; then increase temperature to 280°C at 5°C/min and maintain for 3 min. The carrier gas was helium with a flow rate of 1.0 mL/min. A QC sample is set for every certain number of experimental samples in the sample queue to detect and evaluate the stability and repeatability of the system.
3. Mass spectrometry conditions: 5977B MSD mass spectrometer (Agilent) was used for mass spectrometry analysis. The 5977B MSD conditions are as follows: inlet temperature 280°C; ion source temperature 230°C; transfer line temperature 250°C. *electron* impact ionization (EI) source, electron energy 70 eV; use SCAN/SIM mode to detect the object to be measured. MSD ChemStation software was used to extract the chromatographic peak area and retention time. Draw a calibration curve and calculate the content of free fatty acids in the sample.

### 2.4 Proteomics testing

1. Muscle protein extraction and digestion: Take the samples out from-80°C, add 4 times the volume of lysis buffer (8 M urea, 1% protease inhibitor), and lyse by sonication. Centrifuge at 12,000 g for 10 min at 4°C to remove cell debris, transfer the supernatant to a new centrifuge tube, and measure the protein concentration using a BCA kit. An equal amount of each sample protein was subjected to enzymatic hydrolysis. First, dithiothreitol (DTT) was added to a final concentration of 5 mM and reduced at 56°C for 30 min. Then iodoacetamide (IAA) was added to a final concentration of 11 mM. Incubate at room temperature in the dark for 45 min. Add TEAB to dilute the urea, making sure the concentration is below 2 M. Add trypsin at a ratio of 1:50 (protease: protein, m/m) and hydrolyze overnight. Then add trypsin at a ratio of 1:100 (protease: protein, m/m) and continue enzymatic hydrolysis for 4 h. Use a C18 desalting column to desalt the sample. 100% acetonitrile activates the desalting column, and 0.1% formic acid balances the column. Load the sample onto the column. Then use 0.1% formic acid to wash the column to wash away impurities. Finally, use 50% acetonitrile to elute and collect the flow. Wear liquid and freeze-dry.
2. Protein extract analysis: The peptides were dissolved in liquid chromatography mobile phase A and then separated using the EASY-nLC 1,000 ultra-high performance liquid phase system. Mobile phase A is an aqueous solution containing 0.1% formic acid; mobile phase B is an aqueous solution containing 0.1% formic acid and 80% acetonitrile. Liquid phase gradient setting: 0-10 min, 8% ~ 12% B; 10–102 min, 12% ~ 30% B; 102–133 min, 30% ~ 40% B; 133–134 min, 40% ~ 95% B; 134–150 min, 95% B; flow rate maintained at 600 nL/min. The peptide fragments were separated by the ultra-high performance liquid phase system and then injected into the NSI ion source for ionization and then analyzed using the Orbitrap Exploris™ 480 mass spectrometer with the optional FAIMS Pro™ Interface. The compensation voltage CV switched between −45 and − 65 every 1S. Once, Nanospray Flex™ (NSI) ion source was used, the ion spray voltage was set to 2.0 kV, the ion transfer tube temperature was 320°C, the mass spectrometer adopted the data-dependent acquisition mode, and the full scan range of the mass spectrometer was m/z 350–1,500. The resolution of the first-level mass spectrometry is set to 120,000 (200 m/z), the AGC is 300%, and the maximum injection time of C-trap is 50 ms; the second-level mass spectrometry detection adopts the “Cycle Time” mode, and the resolution of the second-level mass spectrometry is set to 15,000 (200 m/z), the AGC is 75%, the maximum injection time is 22 ms, the peptide fragmentation collision energy is set to 33%, and the mass spectrometry detection raw data (.raw) is generated.
3. Data analysis and screening of differentially expressed proteins: Secondary mass spectrometry data were retrieved using Proteome Discoverer (v2.4.1.15). Search parameter settings: The database is Uniprot database, with anti-library added to calculate the false positive rate (FDR) caused by random matching, and common contaminating libraries added to the database to eliminate the impact of contaminating proteins in the identification results; enzyme digestion The mode is set to Trypsin/P; the number of missing cleavage sites is set to 2; the mass error tolerance of the primary precursor ion for First search and Main search is set to 20 ppm and 5 ppm respectively, and the mass error tolerance of the secondary fragment ion is 20 ppm. Cysteine alkylation was set as a fixed modification, and variable modifications were set as methionine oxidation, protein N-terminal acetylation, and glutamine and asparagine deamidation. The FDR for protein identification and PSM identification was set to 1%. Use GO annotation and KEGG pathways to analyze differentially expressed proteins in different parts of pork.

## 3 Results

### 3.1 Lipids in different parts

**Figure 2:**
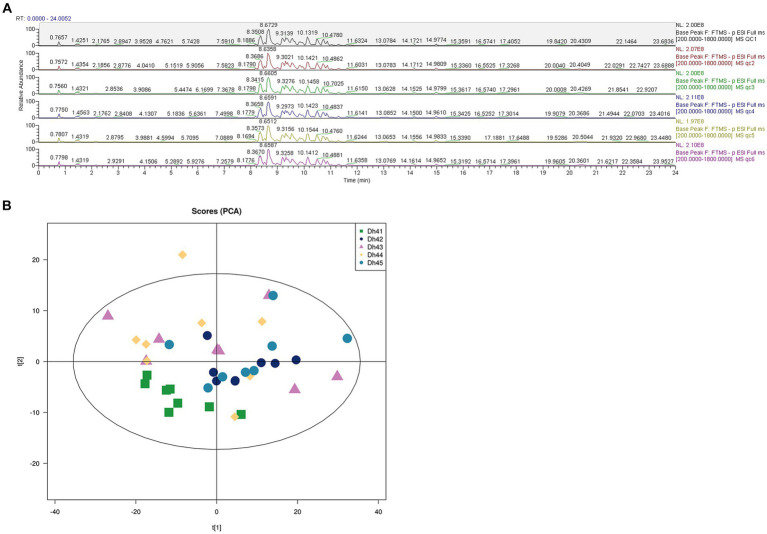
**(A)** Positive and negative ion mode base peak overlay spectra of QC samples **(B)** PCA score plot.

Comprehensively evaluates the stability of the instrument, the repeatability of the experiment, and the reliability of the data quality through six quality control items, uses UHPLC–MS/MS lipidomic to detect the differences in different combinations of muscle lipids. QC samples were used during the analysis process, and the BPC spectra of the QC samples were overlapped and compared (Figure 2A). The results show that the chromatographic peak response intensity and retention time of each QC sample basically overlap, indicating that the mass spectrometer has good stability when detecting the same substance at different times (21). Therefore, the lipidomic method used in this study has high reproducibility and reliability.

The principal component analysis (PCA) score chart shows (Figure 2B) that the QC samples have an aggregation trend, further indicating that the experimental data is relatively reliable.

**Figure 3:**
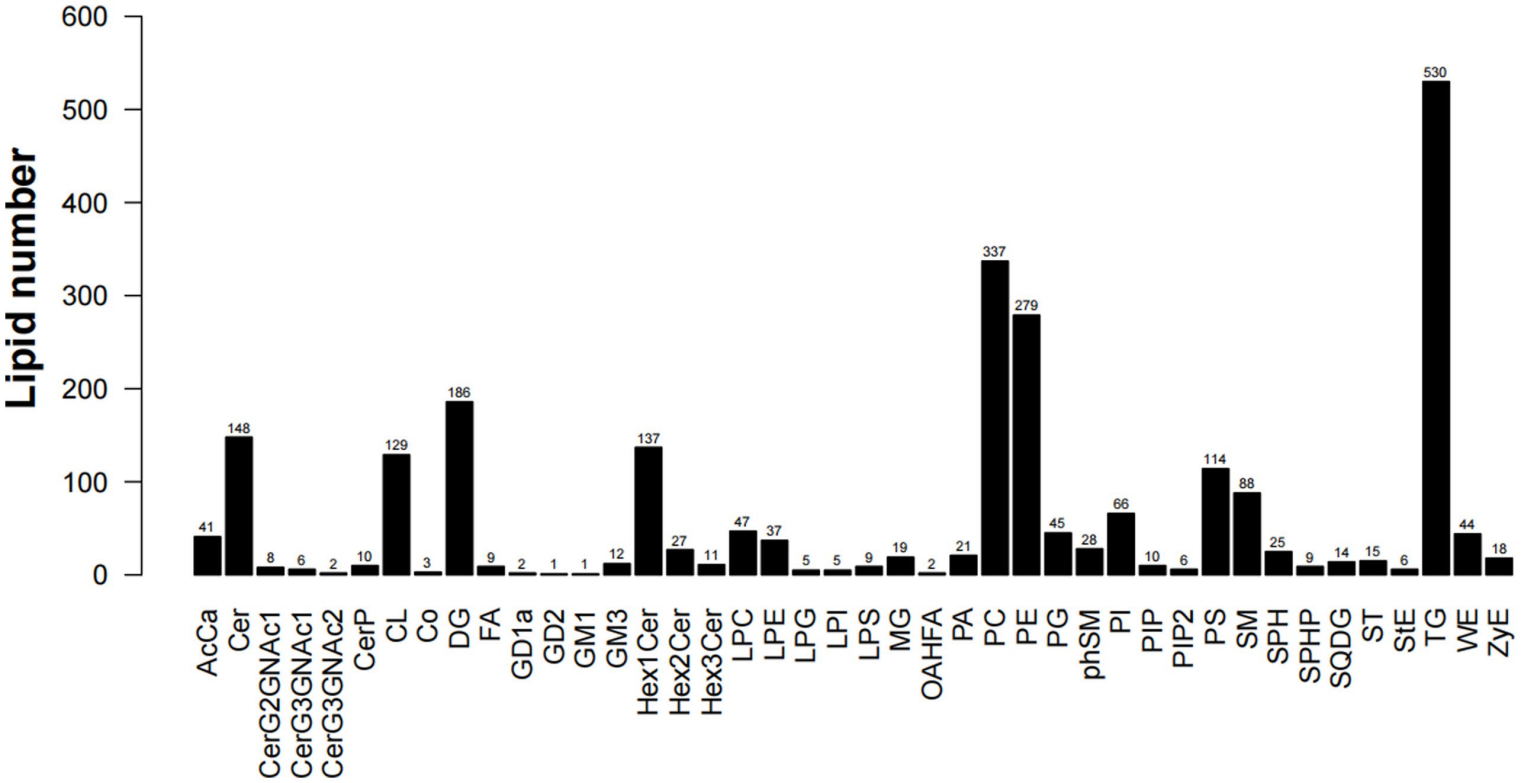
Lipid composition and quantity statistics.

Lipid composition refers to the types and proportions of lipids in the sample. Lipid composition analysis is one of the main contents of lipid data analysis. The composition of lipids is sample-specific. Different types of samples, such as cell membranes, mitochondria, endoplasmic reticulum, etc., contain different types and proportions of lipids at steady state. On the other hand, participating in different physiological response processes, the lipid composition will also change accordingly, which will lead to changes in the biophysical properties and functions of the membrane (22). Through lipid composition analysis, a total of 42 lipids were found in five parts of the muscles(S1), among which TG content is the highest, followed by PC and PE; there are certain differences in lipid content between different parts, but the overall trend is the same. As shown in Figure 3, the triglyceride (TG) in LDM is significantly higher than the other four parts, phosphatidylcholine (PC) in BM and PM was higher than LDM, LM and FM, and phosphatidylethanolamine (PE) in PM was higher than the other four sites. This shows that LDM contains higher energy per unit mass, but TG is usually the cause of atherosclerosis and many diseases. PC has dual lipophilic and hydrophilic properties, which plays a positive role in preventing skin moisture loss and clearing blood vessels. Phosphatidylethanolamine (PE) is the most abundant phospholipid in the nervous system and regulates the development and function of neurons (23).

### 3.2 Fatty acids in different parts

**Figure 4:**
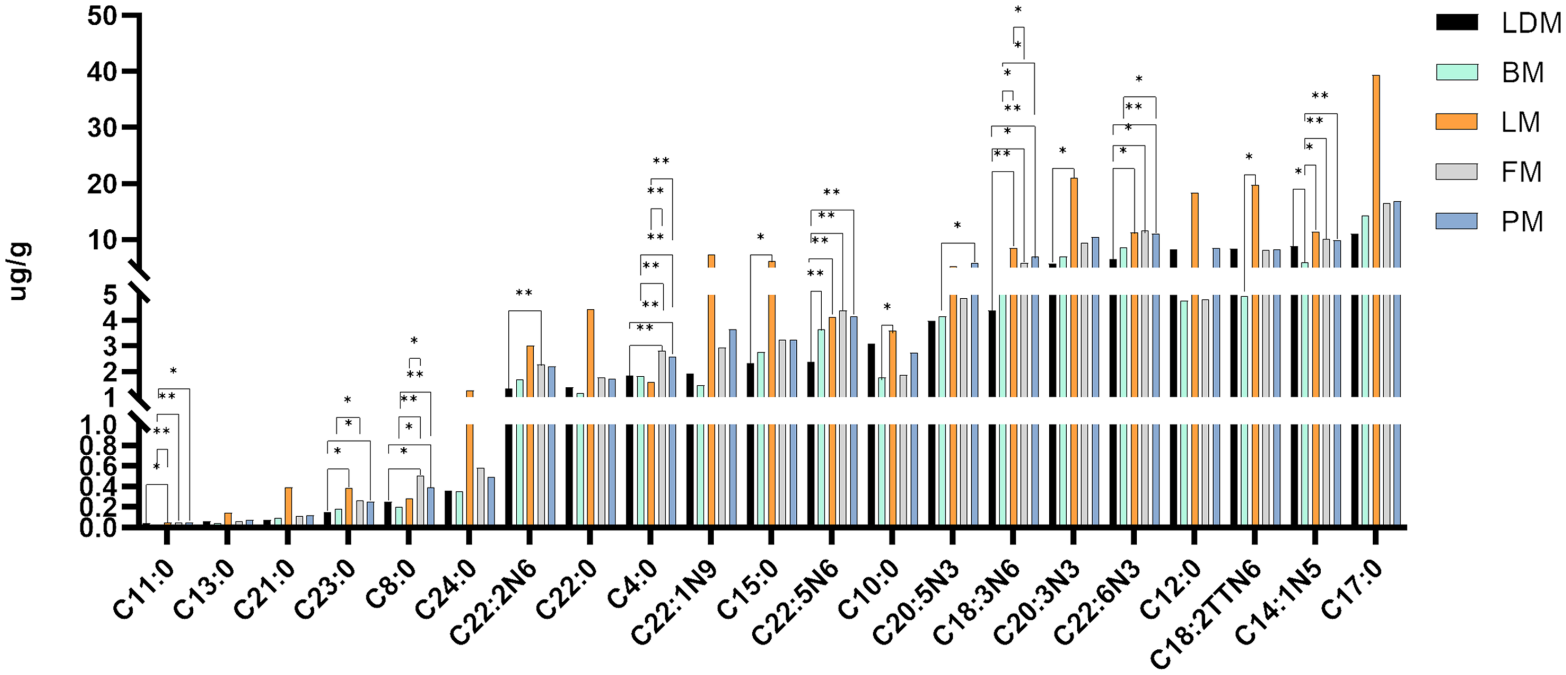
Fatty acid content and differences in different parts.

We organized and drew the data on fatty acid content and differences between different parts of pork. As shown in Figure 4 and Supplementary Figure 1, there are significant (*p* < 0.05) or extremely significant (*p* < 0.01) differences in fatty acids between the two groups. Includes: C11:0, C23:0, C8:0, C20:2 N6, C4:0, C15:0, C22:5 N6, C10:0, C20:5 N3, C18:3 N6, C20:3 N3, C22:6 N3, C18:2TTN6, C14:1 N5, C22:5 N3, C17:1 N7, C20:3 N6, C22:4 N6, C20:2 N6, C20:4 N6, C16:1 N7, C18:2 N6, C16:0, C18:1 N9. From the data results, it can be seen that the two fatty acids C4:0 and C18:3 N6 have the most significant differences among different parts, C8:0, C22:5 N3, and C20:3 N6 have the second most significant differences among different parts, and C11:0, C22:5 N6, C22:6 N3, C14:1 N5, C22:4 N6, C20:4 N6, C18:2 N6 are also significantly different between many parts. In addition, it should be noted that the fatty acid content of FM group and PM group is not significant (*p* > 0.05).

The fatty acid content and differences in different parts are shown in Figure 4. The fatty acid profiles of different parts are shown in Table 1. We summarized 12 fatty acids accounting for about 95% of the total fatty acids and three other calculated traits. The SFA, MUFA and PUFA proportions of LDM were 43, 49 and 8% respectively, the SFA, MUFA and PUFA proportions of BM were 41, 46 and 13% respectively, and the SFA, MUFA and PUFA proportions of LM were 43%, respectively., 47 and 10%, the proportions of SFA, MUFA and PUFA of FM were 40, 48 and 12% respectively, and the proportions of SFA, MUFA and PUFA of PM were 41, 47 and 12%, respectively.

**Table 1:** Fatty acid profiles of different parts.

Class	LDM	BM	LM	FM	PM
Myristic acid (C14:0)	358.81	229.91	876.08	313.49	364.66
Palmitic acid (C16:0)	4440.981	3803.43	8414.83	4749.03	4830.76
Stearic acid (C18:0)	3108.54	2796.64	7200.48	3363.57	3568.11
Arachidic acid (C20:0)	32.98	23.58	108.54	34.68	37.99
Palmitoleic acid (C16:1n-7)	991.73	651.55	2030.26	966.71	974.90
Oleic acid (C18:1n-9)	8005.91	6717.27	15564.69	9201.50	9085.16
Eicosenoic acid (C20:1n-9)	158.88	116.88	562.85	198.66	231.90
Linoleic acid (C18:2n-6)	1181.55	1678.24	3137.12	1812.42	1962.90
α-linolenic acid (C18:3n-3)	19.08	26.03	91.02	32.06	38.71
Eicosadienoic acid (C20:2n-6)	43.64	51.39	184.12	87.26	85.79
Homolonolenic acid (C20:3n-6)	25.13	34.07	45.06	38.38	41.00
Arachidonic acid (C20:4n-6)	184.93	343.38	348.14	376.82	381.18
SFA	7970.15	6881.10	16675.91	8493.43	8838.44
MUFA	9205.42	7526.25	18267.74	10431.37	10358.82
PUFA	1530.92	2236.95	3964.09	2470.58	2634.23

Unit:ug/g. (1) SFA: Saturated fatty acids, mainly including C14:0, C16:0, C18:0 and C20:0. (2) MUFA: Monounsaturated fatty acids, mainly including C16:1, C18:1n-9 and C20:1. (3) PUFA: Polyunsaturated fatty acids, mainly including C18:2n-6, C18:3n-3, C20:2, C20:3n-6 and C20:4n-6.

The C18:3n-3 and C20:3n-6 of LDM were significantly lower than those of the LM, FM, and PM groups, and the C18:2n-6 and C20:4n-6 were significantly lower than those of the BM, LM, FM, and PM groups (*p* < 0.05); BM’s C14:0, C1 6: 0, C18:0, C20:0, C16:1 n-7, C18:1n-9, C20:1 n-9 are lower than those of LDM, LM, FM, PM four groups, C18:3n-3, C20:2 n-6 are lower than LM, FM, PM three groups; LM’s C14:0, C16:0, C18:0, C20: 0, C16:1 n-7, C18:1n-9, C20:1 n-9, C18:2n-6, C18:3n-3, C20:2 n-6, C20:3n-6 were significantly higher than those of LDM, BM, FM and PM group; the fatty acid profiles of FM and PM were most similar.

From the perspective of fatty acids, the potentially harmful fatty acids of LM (C14:0, C16:0) are different from the beneficial monounsaturated fatty acids and polyunsaturated fatty acids (C18:1n-9, C20:1n −9, C20: 2n −6, C20:3n-6) are higher in content than other parts, and the content of potentially harmful fatty acids and beneficial monounsaturated fatty acids and polyunsaturated fatty acids in BM are lower than in other parts. Thirteen fatty acids (C13:0, etc.) were not significantly different among the five parts of pork (*p* > 0.05); C4:0 and C18:3 N6 showed significant(*p* < 0.05) or extremely significant (*p* < 0.01) differences among the most groups; followed by C8:0, C22:5 N3, C20:3 N6 and C18:2 N6 showed significant or extremely significant differences among many groups; in addition, C11:0, etc. Seven lipids showed significant or extremely significant differences among some comparison groups; 10 lipids such as C22:2 N6 only showed significant (p < 0.05) or extremely significant (*p* < 0.01) differences in one comparison group. The SFA we obtained accounted for 40–43% of the total fatty acids, the MUFA accounted for 46–49%, and the PUFA proportion ranged from 8 to 13%. FM has the lowest SFA ratio, LDM and LM have the highest SFA ratio; LDM has the highest MUFA ratio and the lowest PUFA ratio, and BM has the lowest MUFA ratio and the highest PUFA ratio.

Composition of similar fatty acids may be due to similar physiological functions or participation in the same biological process, C14:0, C16:0, C18:0, C20:0, C16:1n −7 and C18:1n-9, C20:1 n-9 and C18:2n-6, C18:3n-3, and C20:3n-6 may have certain relevance in terms of function or biological processes involved.

### 3.3 Characteristic lipids in different parts

The study found that there are differences in lipid content in different parts of muscles. To further understand the marker lipids in different parts, liquid chromatography-mass spectrometry (LC–MS) was used to determine the lipid differences in LDM, BM, LM, FM and PM. In the comparison between LDM and BM, LM, FM, and PM, a total of 1,103, 645, 544, and 999 lipids were screened out that showed significant differences (*p* value <0.05); Compared with LM, FM, and PM, 117, 479, and 204 lipids showed significant differences, respectively, in BM; when LM was compared with FM and PM groups, 29 and 43 lipids showed significant differences respectively; when FM and PM were compared, 271 lipids showed significant differences. To improve the reliability of the data, we used VIP to further select the differential lipids initially screened (24). The results are shown in S1, taking the LDM and BM groups as an example, we finally obtained 157 significantly different lipids (DAL, VIP > 1; *p* < 0.05) for further study, in which 85 lipids were down-regulated and 72 lipids were up-regulated.

### 3.4 Lipid metabolism pathways

**Figure 5:**
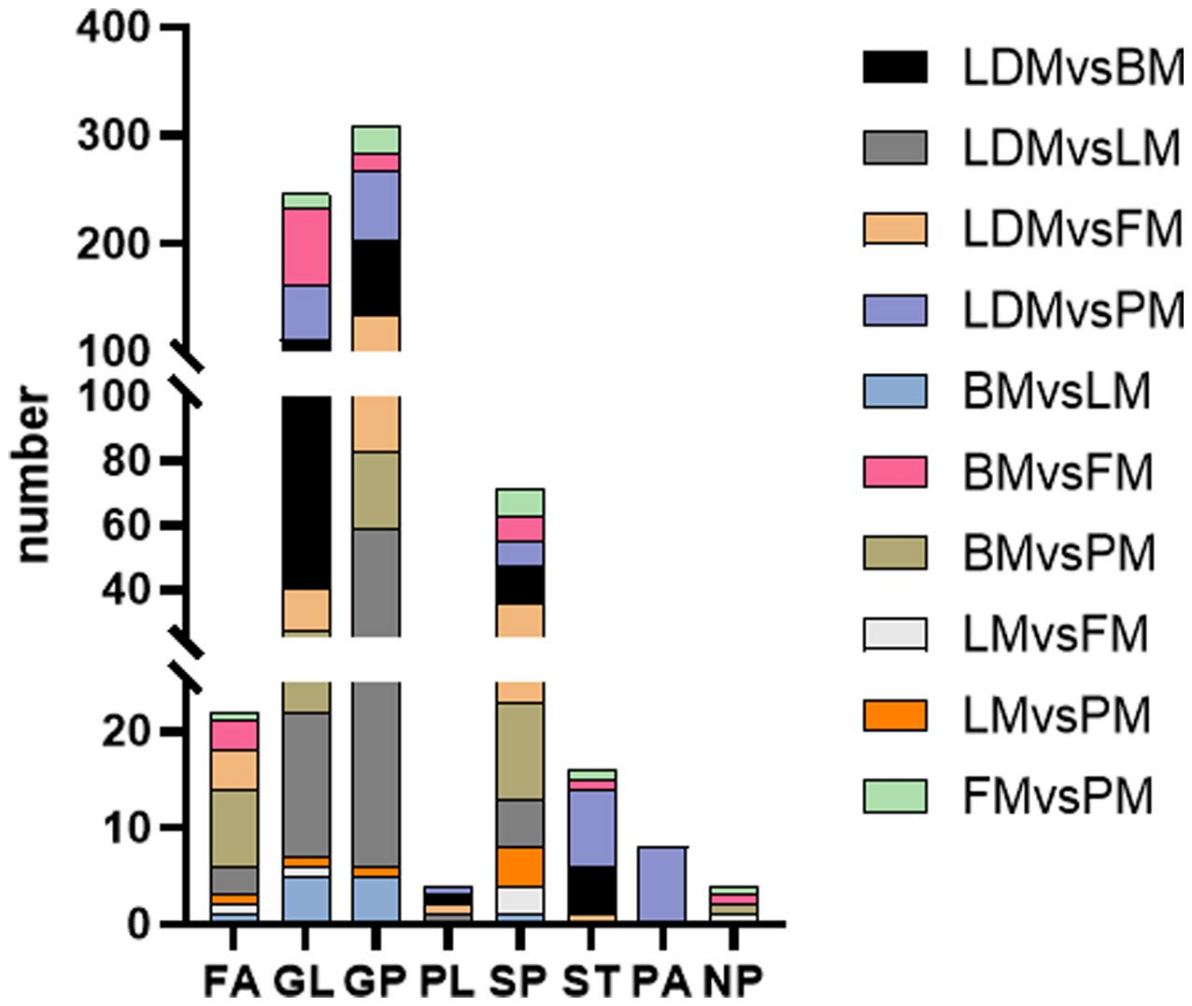
Differential lipid metabolism pathway enrichment in different parts.

This study identified a total of 286 extremely significantly different lipids (VIP > 1, *p* < 0.01). To understand the synthesis, degradation and transport processes of lipids *in vivo*, as well as their relationship with other metabolic pathways, we used the lipid search database to perform metabolic pathway enrichment analysis of the identified lipids (Figure 5). The results showed that GP metabolism is the most important lipid metabolism pathway, followed by SP and GL metabolism. This result is consistent with previous results on lipid metabolism pathways in different parts of donkey meat (23).

These three pathways may be the key metabolic pathways leading to lipid differences in different parts of Duhua pigs (25). After sorting the data, it can be concluded that compared with LDM and BM, the TG level in GL metabolism is significantly higher than CL, DG, and MG; compared with LDM and LM, the PE level in GL metabolism is significantly higher than CL and PC; LDM and FM compared with BM, the PC level in GL metabolism is close to the PE level; compared with LDM and PM, the PC level is slightly higher than PE; compared with BM and LM, the GP metabolism and GL metabolism levels are consistent; compared with BM and FM, the PC level in GL metabolism is slightly higher than that of PE. TG levels were significantly higher than DG and MG; BM compared with PM, PC levels in GP metabolism were significantly higher than CL and PG; LM compared with FM, SPH levels in SP metabolism were higher than CerP; LM compared with PM, The level of SPH in SP metabolism was higher than that of SM and SPHP; compared with PM, the level of PC in GP metabolism was significantly higher than that of FM. These results show that, there are differences in the metabolic pathways of GP, GL, and SP in different combinations of parts of Duhua, resulting in differences in the metabolic levels of certain lipids (such as TG, PC and PE). It provides important information for increasing the IMF content of pork and improving the flavor of pork.

### 3.5 Protein identification and quantification

To detect the protein expression patterns in different parts of Duhua pig, five longissimus dorsi muscles (LDM), belly meat (BM), loin meat (LM), foreleg meat (FM), and hip tip meat (PM) were selected, 15 samples from the site were analyzed by DIA proteomics (26–28). A total of 3,333 proteins and 22,970 peptides were identified in these samples, consistent with the abundance and normal distribution of the identified proteins.

**Figure 6:**
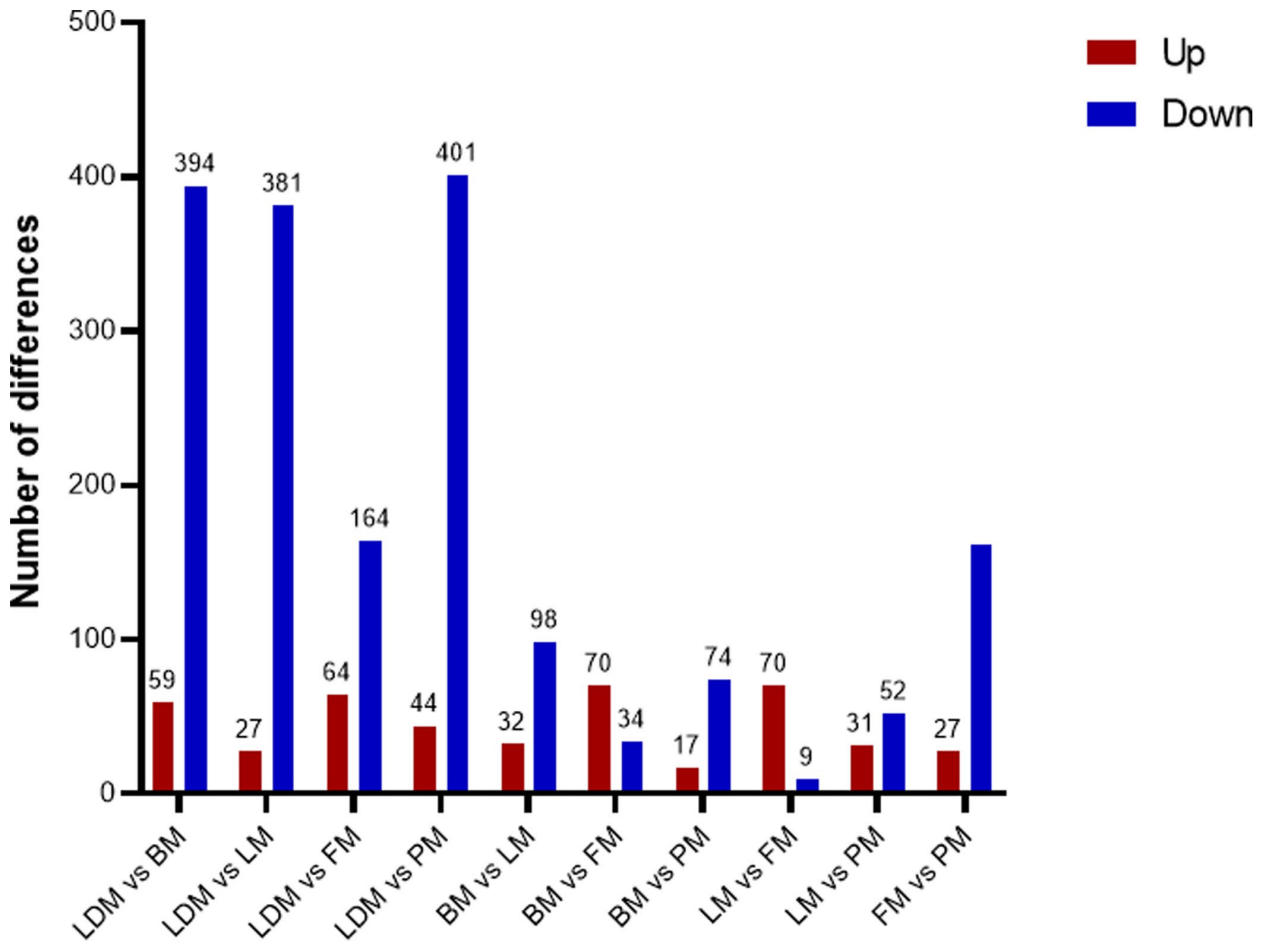
Number of differential proteins between different parts of pork.

**Figure 7:**
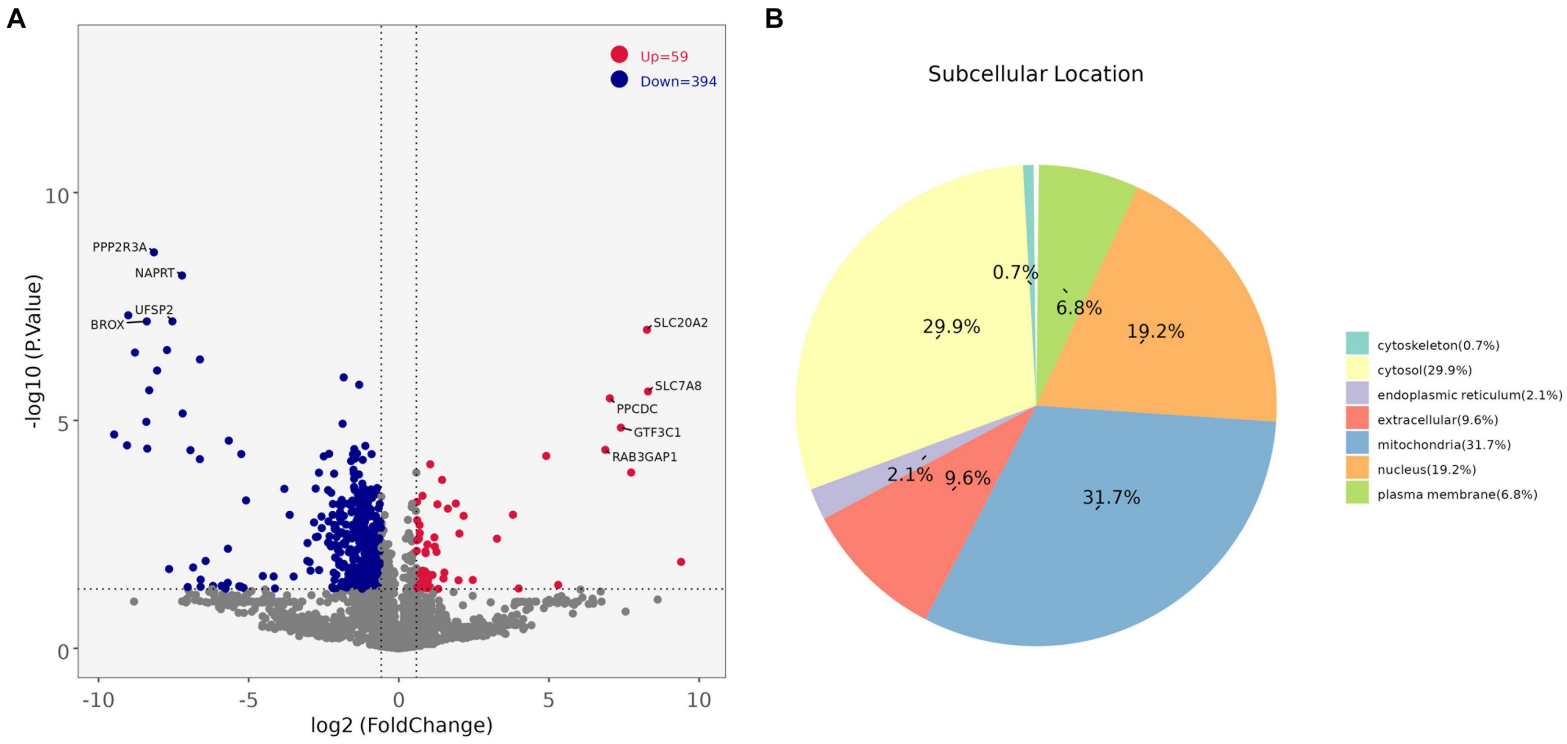
**(A)** Protein volcano map **(B)** DAPs subcellular localization map.

Comparing the proteins identified in each group, the number of differential proteins between the three groups of LDM vs. BM, LDM vs. LM, and LDM vs. PM was higher than that of other groups. Among the identified proteins, compared with the BM group, the expression of 59 proteins was significantly up-regulated (FC ≥ 1.5, value of *p* ≤ 0.05), and the expression of 394 proteins was significantly down-regulated (FC ≤ 0.67, value of *p* ≤ 0.05) in the LDM group (Figure 6).The five most up-regulated proteins were SLC20A2, SLC7A8, PPCDC, GTF3C1 and RAB3GAP1, and the five most strongly down-regulated proteins were PPP2R3A, NAPRT, DHRS11, UFSP2 and BROX (Figure 7A). The results of other groups are shown in Supplementary Figure 2.

The LM vs. FM group had the smallest number of differential proteins, with 70 proteins significantly up-regulated and 9 proteins significantly down-regulated. The five most highly up-regulated proteins were GBA1, MMAA, CTNNA1, MRPS21 and CTNNB1, and the five most strongly down-regulated proteins were NGLY1, PKN2, DHTKD1, HNRNPAB and LMNB2.

Subcellular localization analysis found that except for the BM vs. LM and LM vs. PM groups, the DAPs in each group were mainly mitochondrial proteins, cytoplasmic proteins, and nuclear proteins (Figure 7B and Supplementary Figure 3). The specific distribution of differential proteins in each group was from Table 2. Subcellular localization analysis in the BM vs. LM group found that DAP was mainly cytoplasmic protein (32.4%), extracellular protein (18.9%), and mitochondrial protein (27.0%) (take the top three groups with the highest content). Subcellular localization analysis in the LM vs. PM group found that DAP was mainly cytoplasmic protein (33.3%), extracellular protein (20.5%), mitochondrial protein (12.8%) and nuclear protein (25.6%). The criteria used in our study were appropriate, as confirmed by the hierarchical clustering of DAPs (Supplementary Figure 4). Subcellular localization analysis showed that the most abundant DAP in pork from five different parts was cytoplasmic protein. Through database comparison analysis, the differential proteins were classified into COG/KOG functional categories. It was found that the functions of DAPs are mainly manifested in energy production and conversion, signal transduction mechanisms, post-translational modification, protein turnover, chaperones, and lipid transport metabolism.

**Table 2:** Contents of DAPs in various subcellular locations.

Localization and group	Cytoskel-eton	Cytosol	Endoplas-mic reticulum	Extracellu-lar	Mitochas-ondria	Nucleus	Plasma membrane	Peroxisome
LDMvsBM	0.7	29.9	2.1	9.6	31.7	19.2	6.8	
LDMvsLM	0.4	27.2	1.7	8.8	40.8	13.4	6.7	0.4
LDMvsFM		38.2	0.8	9.9	24.4	18.3	8.4	
LDMvsPM	0.4	33.0	1.5	9.1	29.5	18.6	7.6	0.4
BMvsLM		32.4		18.9	27.0	13.5	8.1	
BMvsFM	1.5	47.1	1.5	7.4	19.1	22.1	1.5	
BMvsPM		38.7	1.6	14.5	17.7	19.4	8.1	
LMvsFM		39.2		5.9	29.4	19.6	5.9	
LMvsPM		33.3		20.5	12.8	25.6	5.1	2.6
FMvsPM	0.85	48.31	1.69	11.86	12.71	19.49	4.24	0.85

### 3.6 Functional enrichment and pathway analysis of DAPs

**Figure 8:**
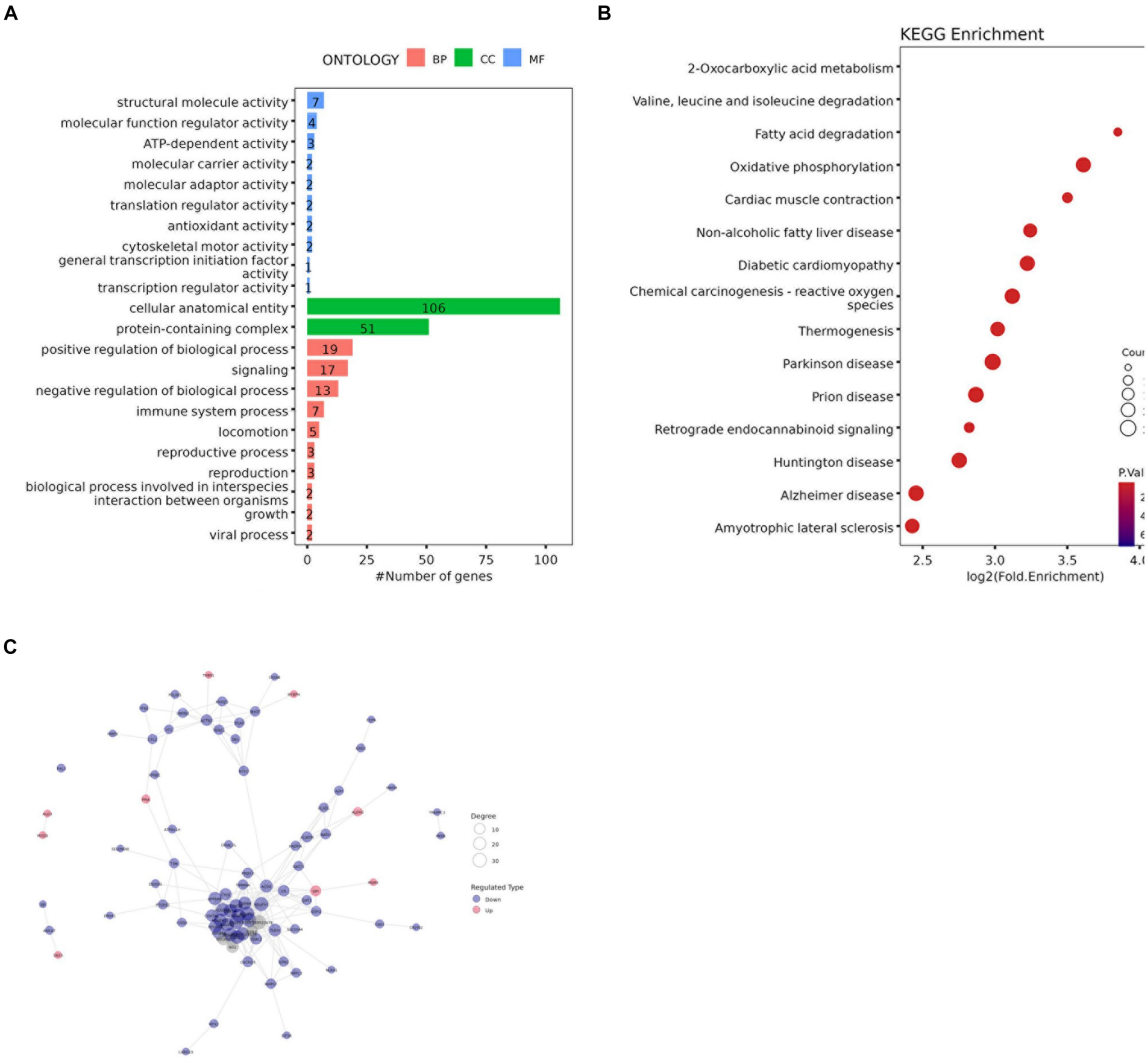
**(A)** GO enrichment analysis results **(B)** Bubble diagram of KEGG pathway enrichment analysis **(C)** Protein-protein interaction network of DAPs.

GO functional enrichment analysis showed that the identified DAPs were enriched in three main GO categories and 34 subcategories. Each comparison group contains the most DAPs in “cellular anatomical entity” and “protein-containing complex” or “cellular anatomical entity” and “binding,” these three items belong to cellular components and molecular function respectively (Figure 8A and Supplementary Figure 5). The BM vs. PM group has the same number of DAPs as ‘binding’ in ‘catalytic activity’ belonging to molecular functions (Ap22).

KEGG analysis was performed to evaluate the potential functions of the identified DAPs. The results of the LDM vs. BM group are shown in Figures 8B, 142 DAPs clustered on 15 KEGG pathways. Among these 15 pathways, 2 pathways are related to lipid metabolism, namely Fatty acid degradation and oxidative phosphorylation. Others The results of the group are shown in Supplementary Figure 6.

To study the interaction between proteins, we used the string database to perform PPI analysis on each group of differential proteins, and constructed PPI networks containing different numbers of DAP (Figure 8C and Supplementary Figure 7). The degree of the differential protein reflects the contribution of DAP to the regulation of the protein network (S2-3). Screened out the top three differential proteins between each group based on the degree, in the BM vs. PM and FM vs. PM groups, there were multiple proteins tied for the top three, in the LM vs. PM group, only one protein contributed to PPI. A total of 31 core proteins (NDUFA6, NDUFA9, ACO2, LOC100524873, COX6B, TUFM, NDUFA12, NDUFB9, RPL26, PSMA5, RPS11, USP2, SBDS, ALDH1B1, MRPL38, RPL35A, SKIC8, CSRP1, DPP3, CNDP2, CHORDC1, COPB2, COPS4, ETF1, FHL3, H2AC20, LMNB2, LOC100738684, PDCD5, SF3B2, YWHAG) were screened out and the expression of most of them DAPs is down-regulated DAP was down-regulated. The expression of RPS11 and USP2 was up-regulated in the BM vs. FM group, the expression of ACO2, CSRP1 and DPP3 was up-regulated in the LM vs. FM group, and the expression of COPB2, LMNB2 and YWHAG was up-regulated in the FM vs. PM group. NDUFA6, NDUFA9, ACO2, and RPS11 expressed strong protein interactions in multiple comparison groups. NDUFA9 contributed to PPI in the most groups, followed by NDUFA6 and ACO2, while NDUFA6 highest contributed to PPI in each group (Supplementary Tables S2–S3).

### 3.7 Relationship between key DAL and DAP expression levels

We filter out the top 3 DALs in each group’s VIP value as key DALs, and then analyze the relationship between the DAPs in the corresponding group and these DALs. Taking the LDM vs. BM group as an example, the key DAL classes belong to PC and TG, of which PC is down-regulated and both TGs are up-regulated, their DAPs showed the most significant differences in the phospholipid binding pathways of lipid oxidation, lipid modification, and lipid binding. Lipid oxidation and lipid modification pathways are related to several DAPs including AUH, HADH, ACADM, ACADL, HADHA. The main function is the regulation of lipid catabolism and lipid transport. Triglycerides are the main form of fatty acid storage and transport in cells and plasma (29–31), this may be the reason why these proteins regulate the upregulation of TG. Proteins related to the phospholipid binding pathway may regulate PC downregulation. SNX5, THBS1, ANXA7, TPP1, CAVIN2, and VDAC2 in the phospholipid binding pathway regulate cell assembly and are related to special transport methods such as endocytosis and membrane vesicles. Relatedly, since PC is the main component of cellular phospholipids (32), and the intracellular membrane structure is mainly composed of phospholipids, the up-regulation or down-regulation of PC may be regulated by these proteins. Among them, ANXA7, which regulates the calcium-dependent phospholipid binding pathway, is a protein unique to the phospholipid binding pathway and shows the most significant difference among several proteins.

### 3.8 Potential biomarkers in different parts of pork

Analyzing the DAL between the LDM group and the other four sites, the results are shown in Tables 3, a total of 35 significantly different lipids simultaneously showed significant differences when comparing LDM with the other four sites (VIP >1; *p* < 0.05), identified as the potential biomarkers between five sites, include 4 CL, 1 Co, 2 DG, 1 LPC, 8 PC, 13 PE, 1 phSM, 2 PI, 1 SM and 2 TG (Appendix, Lipid Table), in which 2 lipids (2 TG) were up-regulated, while 33 lipids were down-regulated. Based on their contribution to the protein network, the top three differential proteins among each group were screened out. A total of 31 proteins were determined to be core proteins and may be potential biomarkers. Among them, NDUFA6, NDUFA9, and ACO2 are most widely distributed or abundant among each group and may play an important regulatory role in the formation of protein characteristics of different parts of pork.

**Table 3:** The key DAL.

DALs						Fold change			
Lipid Ion	Class	FA1	FA2	FA3	FA4	vsBM	vsLM	vsFM	vsPM
TG(16:0_14:0_18:1) + NH4	TG	(16:0)	(14:0)	(18:1)		1.40	1.52	1.33	1.53
TG(16:0_16:0_16:0) + NH4	TG	(16:0)	(16:0)	(16:0)		1.38	1.51	1.33	1.54
CL(78:7)-H	CL	(78:7)				0.36	0.32	0.30	0.32
CL(18:2_18:2_18:2_18:2)-H	CL	(18:2)	(18:2)	(18:2)	(18:2)	0.32	0.27	0.27	0.26
CL(20:5_18:2_18:2_18:2)-H	CL	(20:5)	(18:2)	(18:2)	(18:2)	0.39	0.37	0.41	0.32
CL(74:5)-H	CL	(74:5)				0.48	0.39	0.32	0.34
Co(Q10) + NH4	Co	(Q10)				0.27	0.28	0.28	0.23
DG(34:3e) + Na	DG	(34:3e)				0.25	0.23	0.23	0.19
DG(36:4e) + H	DG	(36:4e)				0.38	0.46	0.53	0.44
LPC(15:0) + H	LPC	(15:0)				0.33	0.28	0.20	0.24
PC(16:0_18:1) + HCOO	PC	(16:0)	(18:1)			0.68	0.67	0.68	0.66
PC(16:0_18:2)-CH3	PC	(16:0)	(18:2)			0.52	0.46	0.46	0.43
PC(18:1_18:1) + HCOO	PC	(18:1)	(18:1)			0.52	0.61	0.62	0.63
PC(16:0_20:4) + HCOO	PC	(16:0)	(20:4)			0.53	0.53	0.54	0.46
PC(35:3) + H	PC	(35:3)				0.16	0.15	0.13	0.14
PC(35:4) + H	PC	(35:4)				0.32	0.28	0.26	0.29
PC(44:11) + H	PC	(44:11)				0.37	0.43	0.48	0.40
PC(44:4e) + H	PC	(44:4e)				0.27	0.29	0.28	0.23
PE(18:1_18:1)-H	PE	(18:1)	(18:1)			0.52	0.46	0.45	0.43
PE(18:0_18:2)-H	PE	(18:0)	(18:2)			0.53	0.46	0.46	0.43
PE(18:1_18:2)-H	PE	(18:1)	(18:2)			0.48	0.39	0.32	0.34
PE(16:1e_20:4)-H	PE	(16:1e)	(20:4)			0.63	0.64	0.71	0.61
PE(18:0_20:4)-H	PE	(18:0)	(20:4)			0.37	0.33	0.30	0.32
PE(18:1e_20:4)-H	PE	(18:1e)	(20:4)			0.61	0.61	0.65	0.63
PE(18:2e_20:4)-H	PE	(18:2e)	(20:4)			0.66	0.63	0.71	0.59
PE(12:0e_6:0) + H	PE	(12:0e)	(6:0)			0.33	0.28	0.20	0.24
PE(16:1e_18:1) + Na	PE	(16:1e)	(18:1)			0.61	0.64	0.66	0.58
PE(18:0_18:1) + Na	PE	(18:0)	(18:1)			0.32	0.28	0.25	0.29
PE(16:0p_20:4) + H	PE	(16:0p)	(20:4)			0.62	0.65	0.66	0.61
PE(16:0p_22:4) + H	PE	(16:0p)	(22:4)			0.55	0.59	0.61	0.56
PE(18:1p_20:4) + H	PE	(18:1p)	(20:4)			0.64	0.62	0.63	0.58
phSM(t38:2) + H	phSM	(t38:2)				0.47	0.52	0.52	0.56
PI(18:0_18:1)-H	PI	(18:0)	(18:1)			0.51	0.47	0.47	0.48
PI(18:1_18:1)-H	PI	(18:1)	(18:1)			0.45	0.53	0.61	0.54
SM(d42:1) + HCOO	SM	(d42:1)				0.56	0.62	0.49	0.69

## 4 Discussion

We identified significantly different lipids and proteins in different parts of pork, analyzed them and screened potential biomarkers.

In the lipidomic analysis, we first screened out the lipids that showed significant differences (*p* value <0.05). To improve the reliability of the data, we further used VIP for selection, and finally obtained the significantly different lipids (VIP > 1; *p* < 0.05) for subsequent analysis. The measurement found that the longissimus dorsi muscle of Duhua pig is the richest in triglycerides, followed by the front leg meat. The triglyceride content in the belly meat and hip tip meat is lower, more suitable for people with high triglycerides and patients with cardiovascular and cerebrovascular diseases (33). Metabolic pathway enrichment analysis was performed on the identified differential lipids, and it was found that GP metabolism is the most important lipid metabolism pathway. GP is mainly related to the membrane structure of cells, which is consistent with PC content and membrane structure-related pathways in subsequent analyses. The results of clustering more DAP is consistent.

GO enrichment analysis results showed that DAPs enriched in each pathway are involved in various structures and functions such as oxidative phosphorylation, energy production, aerobic respiration, protein binding and transmembrane transport, antioxidant and various enzyme activities (34–46). We also found the GO terms that we are concerned about that are closely related to lipid metabolism and lipogenesis: taking LDM vs. BM as an example, 6 DAPs clustered on lipid binding belonging to molecular functions, 9 DAPs aggregate in lipid metabolism processes in biological processes, lipid transport and lipid regulation processes each gather 2 DAPs. There are also fatty acid-related pathways such as fatty acid β-oxidation multienzyme complex (1DAP), fatty acid and its derivatives metabolism (7DAP and 1DAP) process, fatty acid transmembrane transport (1DAP), and fatty acid homeostasis (1DAP).

In KEGG pathway analysis, the reason why many DAPs accumulate in human disease-related pathways may be related to TG content, the accumulation of TG can cause various diseases (47, 48). Among the pathways directly related to lipids that we focus on; the identified DAPs mainly focus on fatty acid degradation and oxidative phosphorylation related to lipid metabolism. Relevant pathways include citric acid (TCA) cycle, pyruvate metabolism, peroxisome proliferator-activated receptor (PPAR) signaling pathway, cholesterol metabolism, fatty acid metabolism, ascorbic acid and aldehyde metabolism, β-alanine metabolism, and fatty acid degradation, glyceride metabolism, glycolysis/gluconeogenesis, glucagon signaling, amino acid biosynthesis, oxidative phosphorylation, cAMP signaling, arginine and proline metabolism, and apelin signaling pathways, etc. The PPAR signaling pathway plays a central role in adipogenesis (24, 49). The glucagon signaling pathway reduces lipid synthesis by increasing lipid oxidation and VLDL assembly in bovine hepatocytes, thereby reducing hepatic fat accumulation (50). The Apelin signaling pathway inhibits hormone-stimulated acute lipolysis by reducing perilipin phosphorylation (51), and its receptor APJ is expressed in various tissues including adipose tissue (52). In addition, the mitochondria-mediated apoptotic pathway also plays an important role in lipolysis (53). This study verified that APOC3, ACADM and other DAPs belong to the PPAR signaling pathway, and F1RHW4, F1SD45 and PRKAA2 participate in the Apelin signaling pathway and play an important role in energy metabolism and lipogenesis. KEGG analysis results provide further insights into lipid anabolism and catabolism.

Key DALs were screened according to the VIP value of each group, and the top 3 DALs were selected as key DALs. The relationship between key DALs and DAP expression levels in the group was analyzed, proving that TG is regulated by several proteins including AUH, HADH, ACADM, ACADL, and HADHA in the lipid oxidation and lipid modification pathways. PC is regulated by SNX5, THBS1, ANXA7, TPP1, CAVIN2, and VDAC2 in the phospholipid binding pathway.

About potential biomarkers of different parts of pork. Thirty-five significantly different lipids showed significant differences when comparing LDM with the other four parts (VIP >1; *p* < 0.05), identified as potential biomarkers. Protein markers identified a total of 31 core proteins according to their contribution (degree) to the protein network, and analyzed three important core proteins: NDUFA6, NDUFA9, and ACO2. Among the lipid markers, two up-regulated TG groups are rich in SFA such as 16:0 and 14:0, and one position is rich in MUFA. The down-regulated of DALs are rich in MUFA and PUFA, which is consistent with the results in Supplementary Table 1 and Table 1 showing that the levels of TG and SFA in LDM are significantly higher and the level of PUFA is significantly lower.

Protein markers, taking the LDM vs. BM group as an example, NDUFA9 targets 24 proteins (S3), regulates pathways related to cellular respiration and transmembrane transport through targeted proteins, and indirectly regulates mitochondrial envelope, organelle inner membrane, organelle envelope and other pathways, consistent with the result that the key DAL in the group contains PC. NDUFA6 targets 37 proteins (except NDUFA9), and part of the regulatory pathways overlap with NDUFA9. In addition to pathways related to cellular respiration and transmembrane transport, some of the proteins targeted by NDUFA6 are concentrated in pathways related to meat color such as cytochrome complexes and heme binding, NDUFA6 may have a certain impact on meat color (54). ACO2 targets 36 proteins, some of the targeted proteins and ACO2 together constitute mitochondrial aconitate hydratase, which participates in the regulation of cellular aerobic respiration, metal cluster binding, production of precursor metabolites and energy, and is closely related to a variety of enzyme activities. NDUFA6 and NDUFA9 indirectly regulate the related pathways of intracellular and intracellular membranes and transmembrane transport. At the same time, NDUFA6, NDUFA9, and ACO2 are all involved in cellular respiration. This corresponds to the result that the key DAL is mainly TG and PC, provides new evidence of the mutual regulation of lipids and proteins.

This study conducted proteomic and lipidomic analysis of different parts of pork from Duhua pigs to gain an in-depth understanding of the composition and biological functions of DAL and DAP in different parts of pork, and identified potential biomarkers between different parts, elucidated the differences in the composition and metabolic pathways of proteins and lipids in different parts, it was found that the interaction between lipid and protein expression in pork may be the reason for the difference in meat quality between different parts, providing new information for the study of metabolic regulation in pork.

## Data availability statement

Our data are publicly available and can be accessed under: ProteomeXchange accession: PXD050436; Project Webpage: http://www.ebi.ac.uk/pride/archive/projects/PXD050436; FTP Download: https://ftp.pride.ebi.ac.uk/pride/data/archive/2024/04/PXD050436.

## Ethics statement

Animal welfare in this study was safeguarded and approved by the Animal Care and Use Committee of Guangdong Academy of Agricultural Sciences. The studies were conducted in accordance with the local legislation and institutional requirements. Written informed consent was obtained from the owners for the participation of their animals in this study.

## Author contributions

ZWu: Data curation, Investigation, Methodology, Project administration, Software, Validation, Writing – original draft, Writing – review & editing. ZWa: Formal analysis, Funding acquisition, Project administration, Resources, Writing – review & editing. PW: Data curation, Investigation, Methodology, Validation, Writing – original draft, Writing – review & editing. LC: Investigation, Methodology, Project administration, Writing – review & editing. JL: Methodology, Supervision, Writing – review & editing. YL: Data curation, Writing – review & editing. LY: Resources, Writing – review & editing. LL: Writing – review & editing, Methodology, Supervision. JZ: Data curation, Project administration, Resources, Writing – review & editing. BH: Funding acquisition, Methodology, Project administration, Resources, Supervision, Writing – review & editing.

## Supplementary material

SUPPLEMENTARY FIGURE 1 Fatty acid content and differences in different parts.

SUPPLEMENTARY FIGURE 2 Volcano_label.

SUPPLEMENTARY FIGURE 3 Subcellular_classify.

SUPPLEMENTARY FIGURE 4 Heatmap.

SUPPLEMENTARY FIGURE 5 GO bar chart.

SUPPLEMENTARY FIGURE 6 KEGG pathway enrichment analysis.

SUPPLEMENTARY FIGURE 7 Differential protein interaction network diagram (PPI).

## References

[1] Frank D Joo ST Warner R. Consumer acceptability of intramuscular fat. Korean J Food Sci Anim Resour. (2016) 36:699–708. doi: 10.5851/kosfa.2016.36.6.699, PMID: 28115880 PMC5243953

[2] Fortin A Robertson WM Tong AKW. The eating quality of Canadian pork and its relationship with intramuscular fat. Meat Sci. (2005) 69:297–305. doi: 10.1016/j.meatsci.2004.07.011, PMID: 22062822

[3] van Laack RL Stevens SG Stalder KJ. The influence of ultimate pH and intramuscular fat content on pork tenderness and tenderization. J Anim Sci. (2001) 79:392–7. doi: 10.2527/2001.792392x, PMID: 11219448

[4] Wu W Zhan J Tang X Li T Duan S. Characterization and identification of pork flavor compounds and their precursors in Chinese indigenous pig breeds by volatile profiling and multivariate analysis. Food Chem. (2022) 385:132543. doi: 10.1016/j.foodchem.2022.132543, PMID: 35287104

[5] Fahy E Subramaniam S Murphy RC Nishijima M Raetz CRH Shimizu T. Update of the LIPID MAPS comprehensive classification system for lipids 1. J Lipid Res. (2009) 50:S9–S14. doi: 10.1194/jlr.R800095-JLR200, PMID: 19098281 PMC2674711

[6] Sun T Wang X Cong P Xu J Xue C. Mass spectrometry-based lipidomics in food science and nutritional health: a comprehensive review. Compr Rev Food Sci Food Saf. (2020) 19:2530–58. doi: 10.1111/1541-4337.12603, PMID: 33336980

[7] De Carvalho CCCR Caramujo MJ. The various roles of fatty acids. Molecules. (2018) 23:2583. doi: 10.3390/molecules23102583, PMID: 30304860 PMC6222795

[8] Wood JD Enser M Fisher AV Nute GR Sheard PR Richardson RI. Fat deposition, fatty acid composition and meat quality: a review. Meat Sci. (2008) 78:343–58. doi: 10.1016/j.meatsci.2007.07.019, PMID: 22062452

[9] Li D Wahlqvist ML Sinclair AJ. Advances in n-3 polyunsaturated fatty acid nutrition. Asia Pac J Clin Nutr. (2019) 28:1–5. doi: 10.6133/apjcn.201903_28(1).0001, PMID: 30896407

[10] Meyer A Montastier E Hager J Saris WHM Astrup A Viguerie N. Plasma metabolites and lipids predict insulin sensitivity improvement in obese, nondiabetic individuals after a 2-phase dietary intervention. Am J Clin Nutr. (2018) 108:13–23. doi: 10.1093/ajcn/nqy087, PMID: 29878058 PMC6600064

[11] Dasilva G Medina I. Lipidomic methodologies for biomarkers of chronic inflammation in nutritional research: ω-3 and ω-6 lipid mediators. Free Radic Biol Med. (2019) 144:90–109. doi: 10.1016/j.freeradbiomed.2019.03.017, PMID: 30902758

[12] Ding M Rexrode KM. A review of lipidomics of cardiovascular disease highlights the importance of isolating lipoproteins. Meta. (2020) 10:163. doi: 10.3390/metabo10040163, PMID: 32340170 PMC7240942

[13] Gong L Yang S Zhang W Han FF Lv YL Xuan LL. Discovery of metabolite profiles of metabolic syndrome using untargeted and targeted LC–MS based lipidomics approach. J Pharm Biomed Anal. (2020) 177:112848. doi: 10.1016/j.jpba.2019.112848, PMID: 31479998

[14] Calder PC. Functional roles of fatty acids and their effects on human health. J Parenter Enter Nutr. (2015) 39:18S–32S. doi: 10.1177/0148607115595980, PMID: 26177664

[15] Abdelhamid AS Brown TJ Brainard JS Biswas P Thorpe GC Moore HJ. Omega-3 fatty acids for the primary and secondary prevention of cardiovascular disease. Cochrane Database Syst Rev. (2018) 11:177. doi: 10.1002/14651858.CD003177.pub4, PMID: 30521670 PMC6517311

[16] Rao A Briskey D Nalley JO Ganuza E. Omega-3 eicosapentaenoic acid (EPA) rich extract from the microalga nannochloropsis decreases cholesterol in healthy individuals: a double-blind, randomized, placebo-controlled, three-month supplementation study. Nutrients. (2020) 12:1869. doi: 10.3390/nu12061869, PMID: 32585854 PMC7353404

[17] Zhao Y Zhao MF Yang ML Wu TY Xu CJ Wang JM. G protein–coupled receptor 30 mediates the anticancer effects induced by eicosapentaenoic acid in ovarian cancer cells. Cancer Res Treat. (2020) 52:815–29. doi: 10.4143/crt.2019.380, PMID: 32138466 PMC7373874

[18] Watanabe Y Tatsuno I. Omega-3 polyunsaturated fatty acids for cardiovascular diseases: present, past and future. Expert Rev Clin Pharmacol. (2017) 10:865–73. doi: 10.1080/17512433.2017.1333902, PMID: 28531360

[19] Herreman L Nommensen P Pennings B Laus MC. Comprehensive overview of the quality of plant-and animal-sourced proteins based on the digestible indispensable amino acid score. Food Sci Nutr. (2020) 8:5379–91. doi: 10.1002/fsn3.1809, PMID: 33133540 PMC7590266

[20] Wyngaarden SL Lightburn KK Martin RC. Optimizing livestock feed provision to improve the efficiency of the Agri-food system. Agroecol Sustain Food Syst. (2020) 44:188–214. doi: 10.1080/21683565.2019.1633455

[21] Palyzová A Guschina IA Řezanka T. Chiral analysis of glycerol phosphates-can bacteria biosynthesize heterochiral phospholipid membranes. J Chromatogr A. (2022) 1676:463267. doi: 10.1016/j.chroma.2022.463267, PMID: 35767906

[22] Hou X Zhang R Yang M Niu N Wu J Shu Z. Metabolomics and lipidomics profiles related to intramuscular fat content and flavor precursors between Laiwu and Yorkshire pigs. Food Chem. (2023) 404:134699. doi: 10.1016/j.foodchem.2022.134699, PMID: 36444028

[23] Li M Zhu M Chai W Wang Y Fan D Lv M. Determination of lipid profiles of Dezhou donkey meat using an LC-MS-based lipidomics method. J Food Sci. (2021) 86:4511–21. doi: 10.1111/1750-3841.15917, PMID: 34535907

[24] Semple RK Chatterjee VKK O’Rahilly S. PPARγ and human metabolic disease. J Clin Invest. (2006) 116:581–9. doi: 10.1172/JCI28003, PMID: 16511590 PMC1386124

[25] Lee ICH Tumanov S Wong JWH Stocker R Ho JWK. Integrative processing of untargeted metabolomic and lipidomic data using MultiABLER. iScience. (2023) 26:106881. doi: 10.1016/j.isci.2023.106881, PMID: 37260745 PMC10227420

[26] Kim NK Park HR Lee HC Yoon D Son ES Kim YS. Comparative studies of skeletal muscle proteome and transcriptome profilings between pig breeds. Mamm Genome. (2010) 21:307–19. doi: 10.1007/s00335-010-9264-8, PMID: 20532784

[27] Ma C Wang W Wang Y Sun Y Kang L Zhang Q. TMT-labeled quantitative proteomic analyses on the longissimus dorsi to identify the proteins underlying intramuscular fat content in pigs. J Proteome. (2020) 213:103630. doi: 10.1016/j.jprot.2019.103630, PMID: 31881348

[28] Yang H Xu X Ma H Jiang J. Integrative analysis of transcriptomics and proteomics of skeletal muscles of the Chinese indigenous Shaziling pig compared with the Yorkshire breed. BMC Genet. (2016) 17:80. doi: 10.1186/s12863-016-0389-y 27296698 PMC4906580

[29] Zhao T Li J Wang Y Guo X Sun Y. Integrative metabolome and lipidome analyses of plasma in neovascular macular degeneration. Heliyon. (2023) 9:329. doi: 10.1016/j.heliyon.2023.e20329, PMID: 37780745 PMC10539639

[30] Shi M Zhang R Jin Q Cui Y Shi J Chen X. Subacute sarin exposure disrupted the homeostasis of purine and pyrimidine metabolism in Guinea pig striatum studied by integrated metabolomic, lipidomic and proteomic analysis. Toxicol Lett. (2022) 367:48–58. doi: 10.1016/j.toxlet.2022.07.008, PMID: 35868497

[31] Pan J Tao C Cao C Zheng Q Lam SM Shui G. Adipose lipidomics and RNA-Seq analysis revealed the enhanced mitochondrial function in UCP1 knock-in pigs. Biochimica et Biophysica acta (BBA)-molecular and cell biology of. Lipids. (2019) 1864:1375–83. doi: 10.1016/j.bbalip.2019.06.017 31271850

[32] Yang Z Chen G Liao G Zheng Z Zhong Y Wang G. UHPLC-MS/MS-based lipidomics for the evaluation of the relationship between lipid changes and Zn-protoporphyrin formation during Nuodeng ham processing. Food Res Int. (2023) 174:113509. doi: 10.1016/j.foodres.2023.113509, PMID: 37986504

[33] Chen C Shi Z Fan X du L Zhou C Pan D. Combined application of high-throughput sequencing and LC-MS-based lipidomics in the evaluation of microorganisms and lipidomics of restructured ham of different salted substitution. Food Res Int. (2023) 174:113596. doi: 10.1016/j.foodres.2023.113596, PMID: 37986459

[34] Langemeyer L Ungermann C. BORC and BLOC-1: shared subunits in trafficking complexes. Dev Cell. (2015) 33:121–2. doi: 10.1016/j.devcel.2015.04.008, PMID: 25898163

[35] Park SY Kim J Kim IJ Choi BK Jung KY Lee S. Reabsorption of neutral amino acids mediated by amino acid transporter LAT2 and TAT1 in the basolateral membrane of proximal tubule. Arch Pharm Res. (2005) 28:421–32. doi: 10.1007/BF02977671, PMID: 15918515

[36] Zelko IN Mariani TJ Folz RJ. Superoxide dismutase multigene family: a comparison of the CuZn-SOD (SOD1), Mn-SOD (SOD2), and EC-SOD (SOD3) gene structures, evolution, and expression. Free Radic Biol Med. (2002) 33:337–49. doi: 10.1016/S0891-5849(02)00905-X, PMID: 12126755

[37] Robichon C Varret M Le Liepvre X Lasnier F Hajduch E Ferré P. Dna JA4 is a SREBP-regulated chaperone involved in the cholesterol biosynthesis pathway. Biochimica et Biophysica acta (BBA)-molecular and cell biology of. Lipids. (2006) 1761:1107–13. doi: 10.1016/j.bbalip.2006.07.007 16950652

[38] McGee AM Douglas DL Liang Y Hyder SM Baines CP. The mitochondrial protein C1qbp promotes cell proliferation, migration and resistance to cell death. Cell Cycle. (2011) 10:4119–27. doi: 10.4161/cc.10.23.18287, PMID: 22101277 PMC3272292

[39] Gaudet P Livstone MS Lewis SE Thomas PD. Phylogenetic-based propagation of functional annotations within the gene ontology consortium. Brief Bioinform. (2011) 12:449–62. doi: 10.1093/bib/bbr042, PMID: 21873635 PMC3178059

[40] Erickson JW Cerione RA. Multiple roles for Cdc42 in cell regulation. Curr Opin Cell Biol. (2001) 13:153–7. doi: 10.1016/S0955-0674(00)00192-7, PMID: 11248548

[41] Wang JF Guan ZP Zhang SL Pei Z Chen YY Pan H. Programmed cell death 5 correlates with disease activity and interleukin-17 in serum and synovial fluid of rheumatoid arthritis patients. Chin Med J. (2013) 126:296–9. doi: 10.3760/cma.j.issn.0366-6999.20122693, PMID: 23324280

[42] Bachelor MA Lu Y Owens DM. L-3-phosphoserine phosphatase (PSPH) regulates cutaneous squamous cell carcinoma proliferation independent of L-serine biosynthesis. J Dermatol Sci. (2011) 63:164–72. doi: 10.1016/j.jdermsci.2011.06.001, PMID: 21726982 PMC3152677

[43] Rawat V Malvi P Della Manna D Yang ES Bugide S Zhang X. PSPH promotes melanoma growth and metastasis by metabolic deregulation-mediated transcriptional activation of NR4A1. Oncogene. (2021) 40:2448–62. doi: 10.1038/s41388-021-01683-y, PMID: 33674745 PMC8026604

[44] Sato K Masuda T Hu Q Tobo T Kidogami S Ogawa Y. Phosphoserine phosphatase is a novel prognostic biomarker on chromosome 7 in colorectal cancer. Anticancer Res. (2017) 37:2365–71. doi: 10.21873/anticanres.11574, PMID: 28476802

[45] Huang MY Liu XY Shao Q Zhang X Miao L Wu XY. Phosphoserine phosphatase as a prognostic biomarker in patients with gastric cancer and its potential association with immune cells. BMC Gastroenterol. (2022) 22:1–10. doi: 10.1186/s12876-021-02073-0, PMID: 34979926 PMC8722028

[46] Fujihira H Masahara-Negishi Y Akimoto Y Hirayama H Lee HC Story BA. Liver-specific deletion of Ngly1 causes abnormal nuclear morphology and lipid metabolism under food stress. BBA-Mol Basis Dis. (2020) 1866:165588. doi: 10.1016/j.bbadis.2019.165588, PMID: 31733337

[47] Xu Z Chen W Wang L You W Wang Y Wang Y. UCP1 knockin induces lipid dynamics and transcriptional programs in the skeletal muscles of pigs. Front Cell Dev Biol. (2022) 9:808095. doi: 10.3389/fcell.2021.808095, PMID: 35096834 PMC8790096

[48] Xu Z Chen W Wang L Zhou Y Nong Q Valencak TG. Cold exposure affects lipid metabolism, fatty acids composition and transcription in pig skeletal muscle. Front Physiol. (2021) 12:748801. doi: 10.3389/fphys.2021.748801, PMID: 34690816 PMC8526723

[49] Yu YH Ding ST. Ectopic expression of porcine peroxisome-proliferator-activated receptor delta regulates adipogenesis in myoblasts. FASEB J. (2007) 21:A703–3. doi: 10.1096/fasebj.21.5.A703-a 17878286

[50] Li Y Ding HY Dong J Ur Rahman S Feng S Wang X. Glucagon attenuates lipid accumulation in cow hepatocytes through AMPK signaling pathway activation. J Cell Physiol. (2019) 234:6054–66. doi: 10.1002/jcp.27258, PMID: 30478902

[51] Than A Cheng YQ Foh LC Leow MK Lim SC Chuah YJ. Apelin inhibits adipogenesis and lipolysis through distinct molecular pathways. Mol Cell Endocrinol. (2012) 362:227–41. doi: 10.1016/j.mce.2012.07.002, PMID: 22842084

[52] Bertrand C Valet P Castan-Laurell I. Apelin and energy metabolism. Front Physiol. (2015) 6:115. doi: 10.3389/fphys.2015.00115, PMID: 25914650 PMC4392293

[53] Li DD Luo Z Ling SC Wu K Chen GH Cheng J. Mitochondrial apoptotic pathway mediated the Zn-induced lipolysis in yellow catfish Peteobagrus fulvidraco. Chemosphere. (2018) 208:907–15. doi: 10.1016/j.chemosphere.2018.05.200, PMID: 30068034

[54] Yu Q Wu W Tian X Hou M Dai R Li X. Unraveling proteome changes of Holstein beef M. Semitendinosus and its relationship to meat discoloration during post-mortem storage analyzed by label-free mass spectrometry. J Proteome. (2017) 154:85–93. doi: 10.1016/j.jprot.2016.12.012, PMID: 28039026

