# Supplementary figures and images for: Integrative analysis of proteomics and lipidomic profiles reveal the fat deposition and meat quality in Duroc × Guangdong small spotted pig
Source: Front Vet Sci. 2024 Apr 10;11:1361441. doi: 10.3389/fvets.2024.1361441 (PMC11041638; doi:10.3389/fvets.2024.1361441)

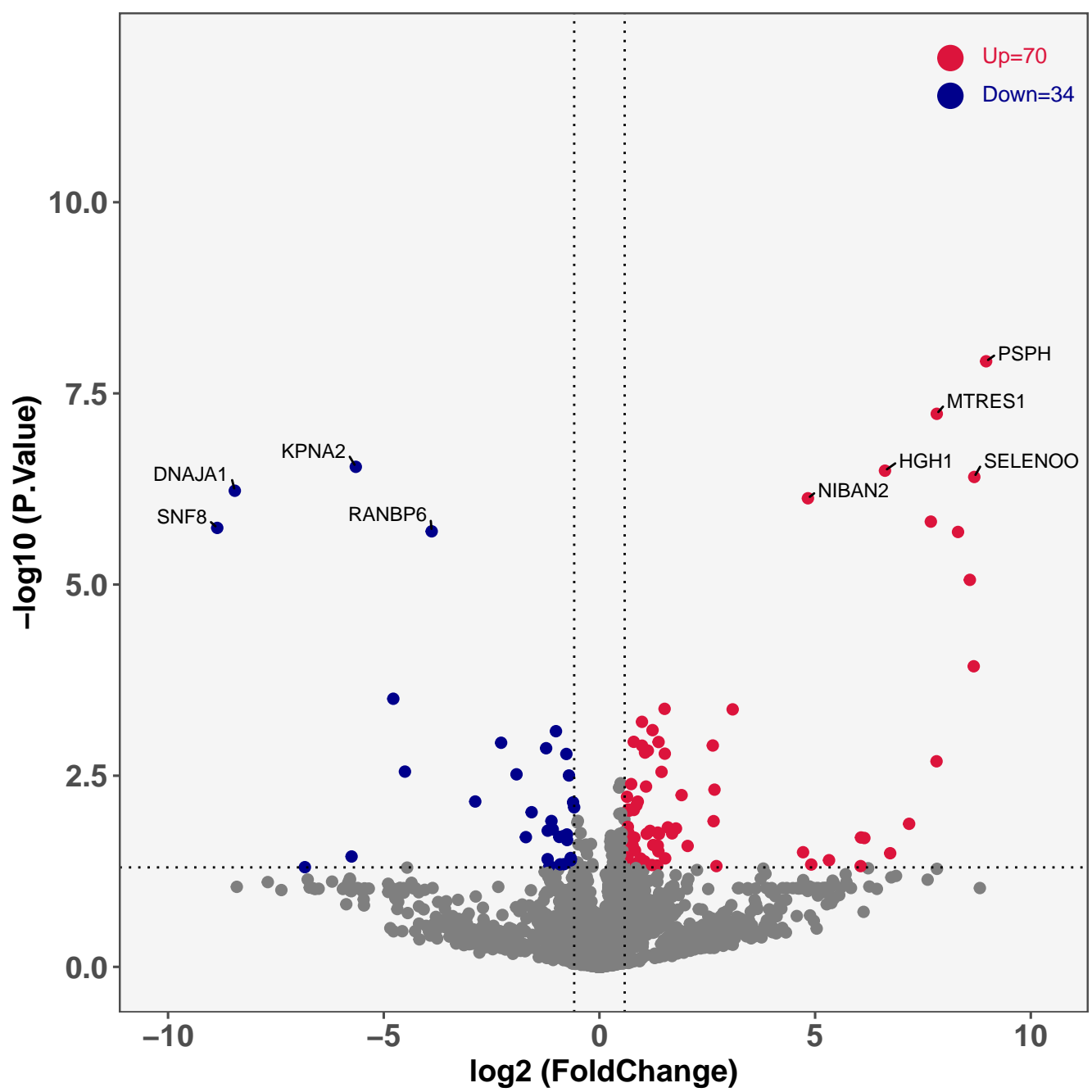

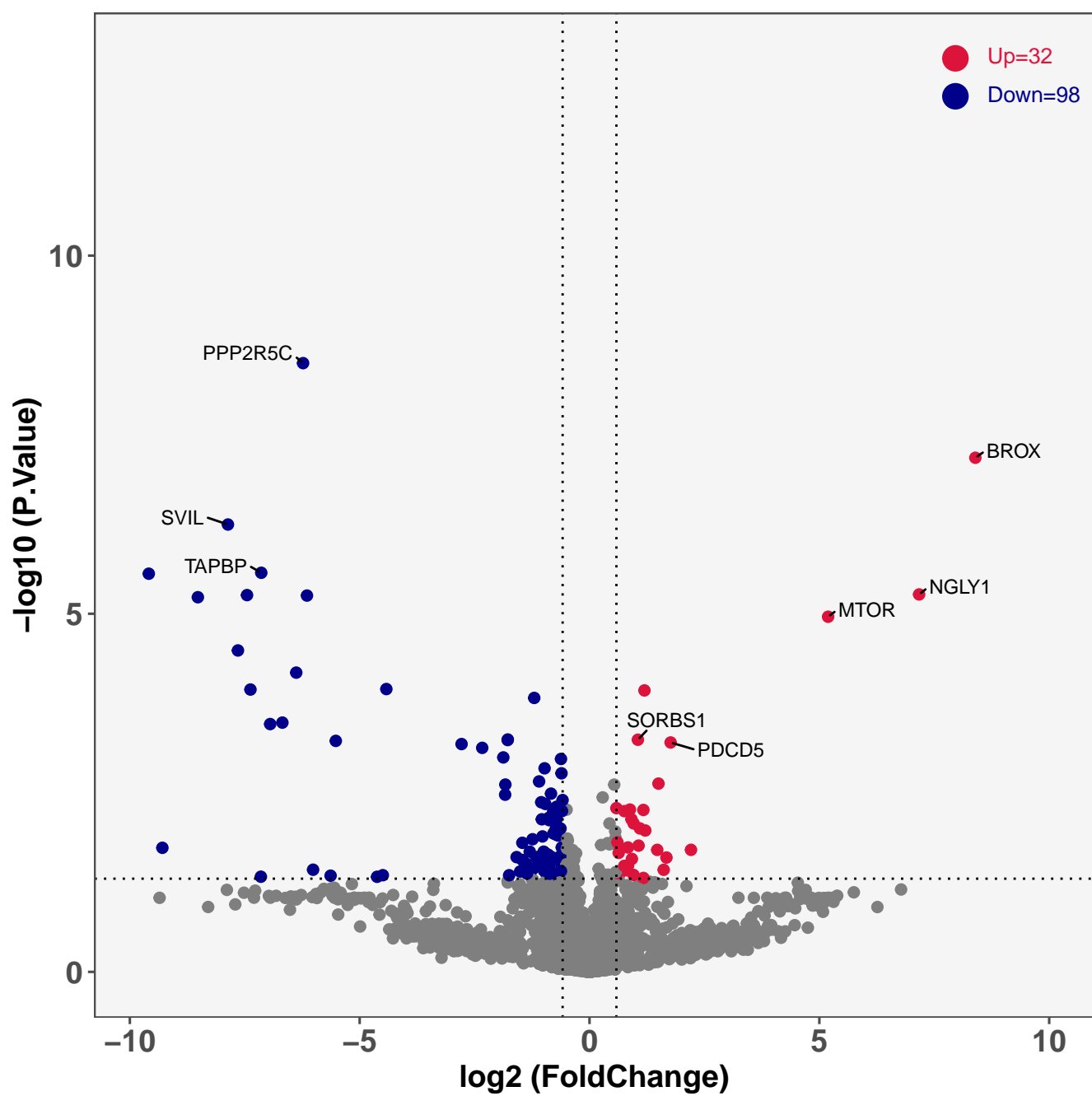

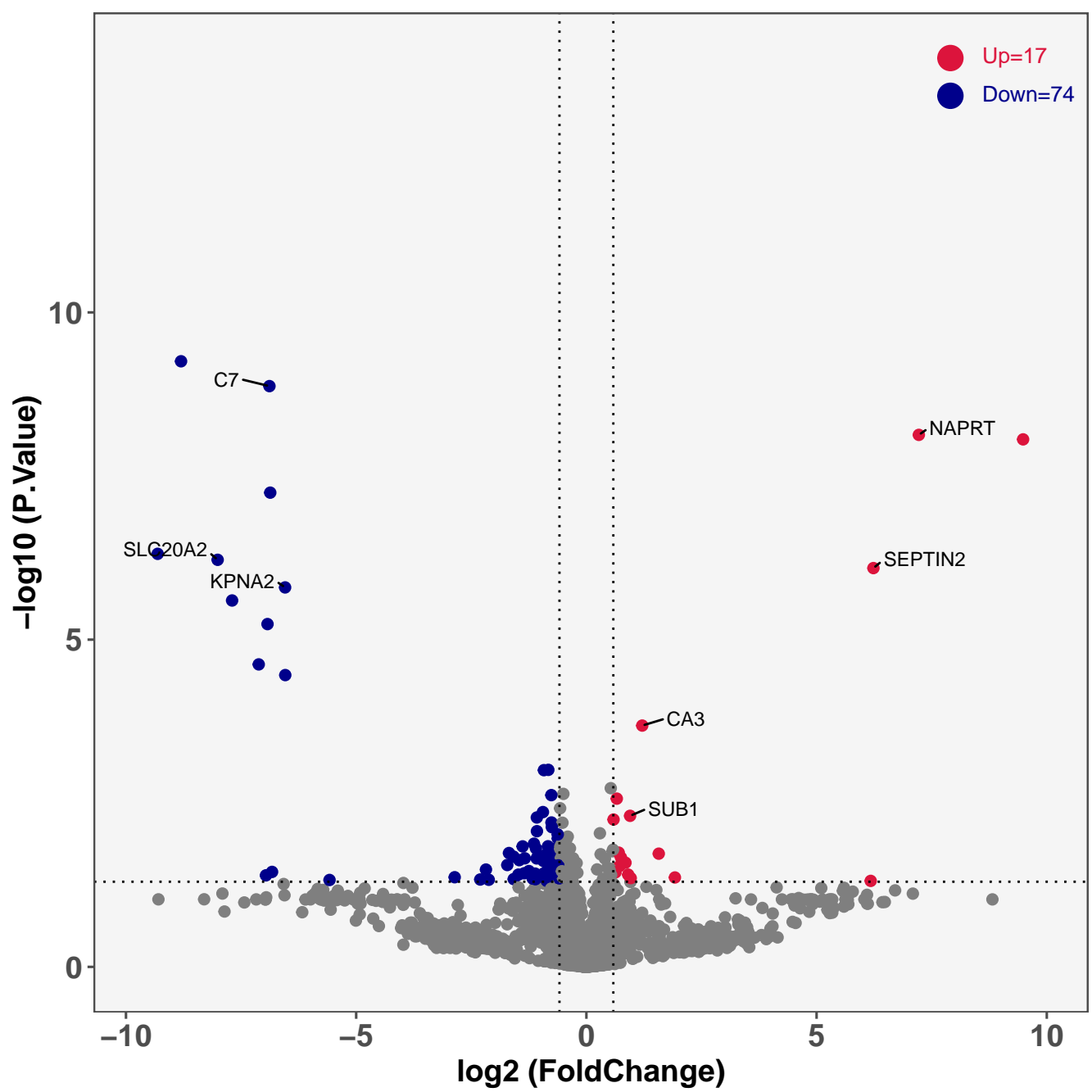

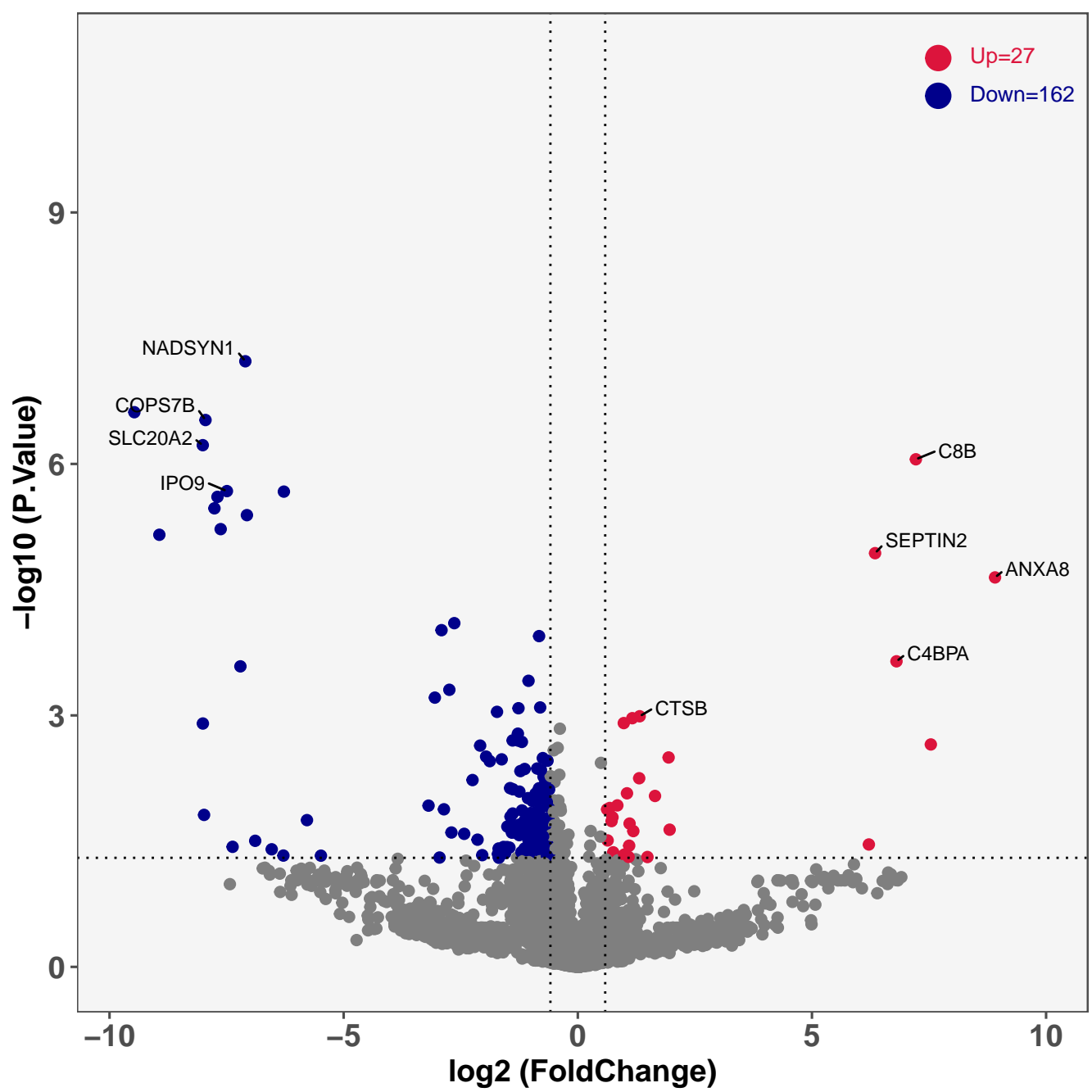

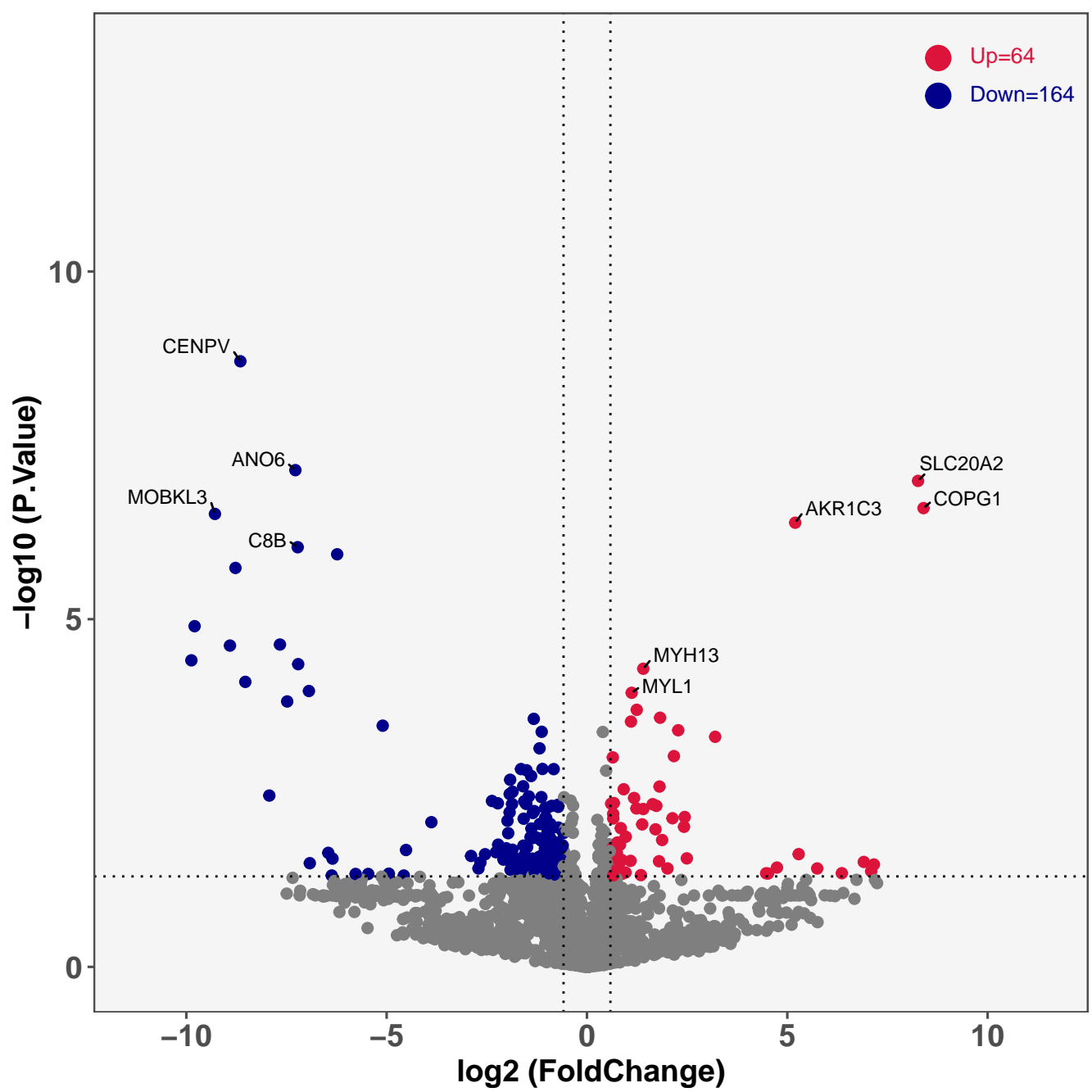

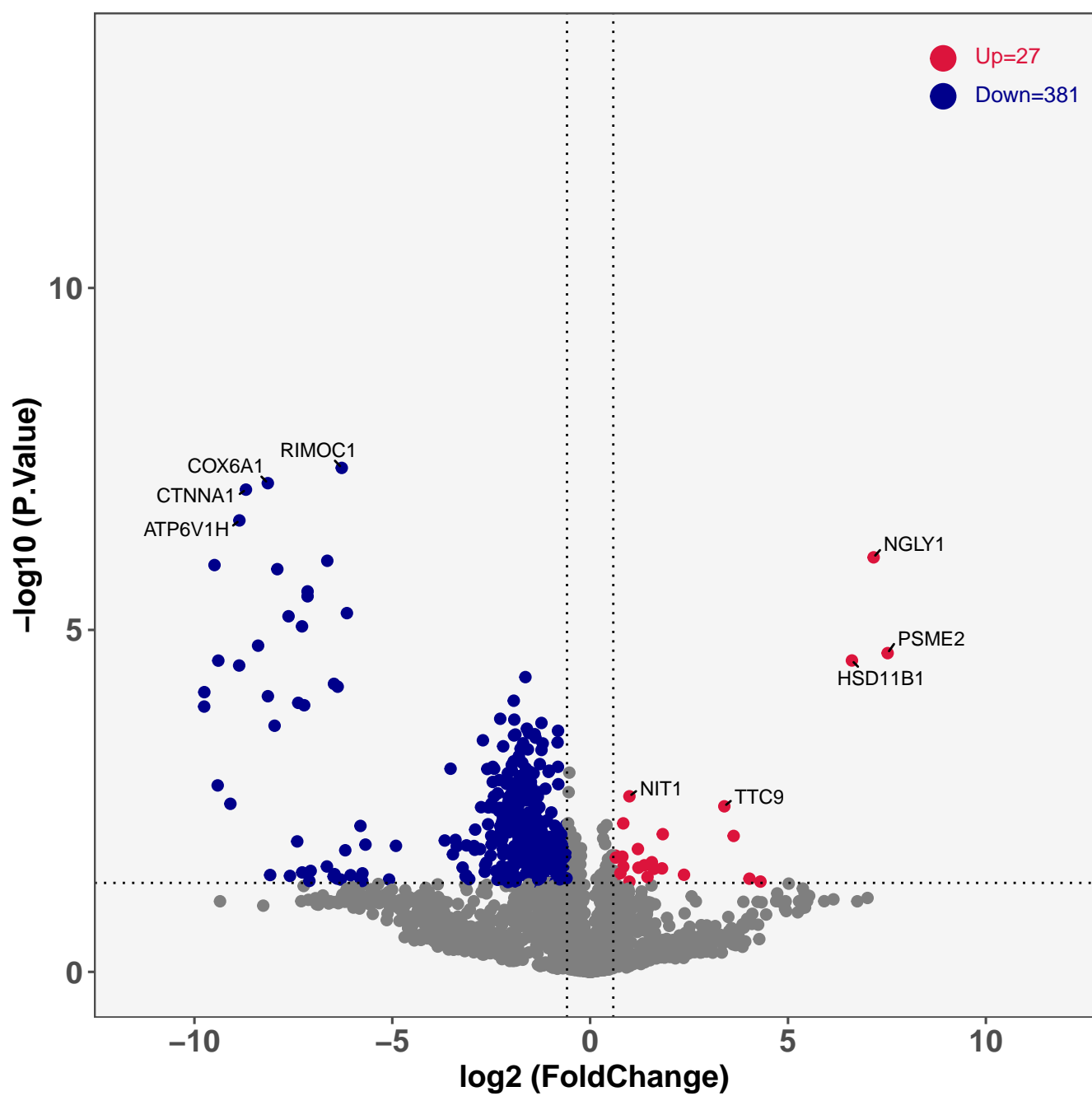

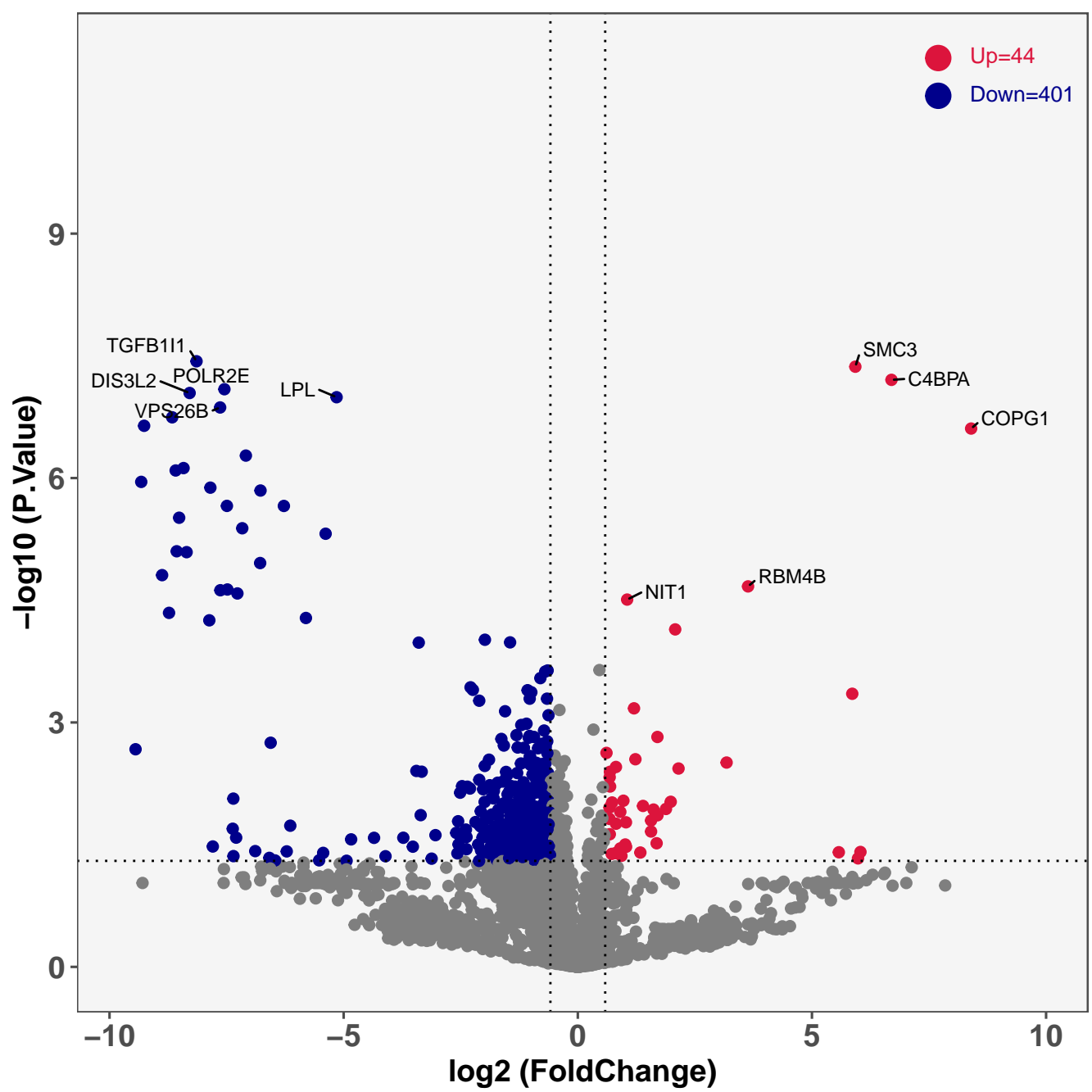

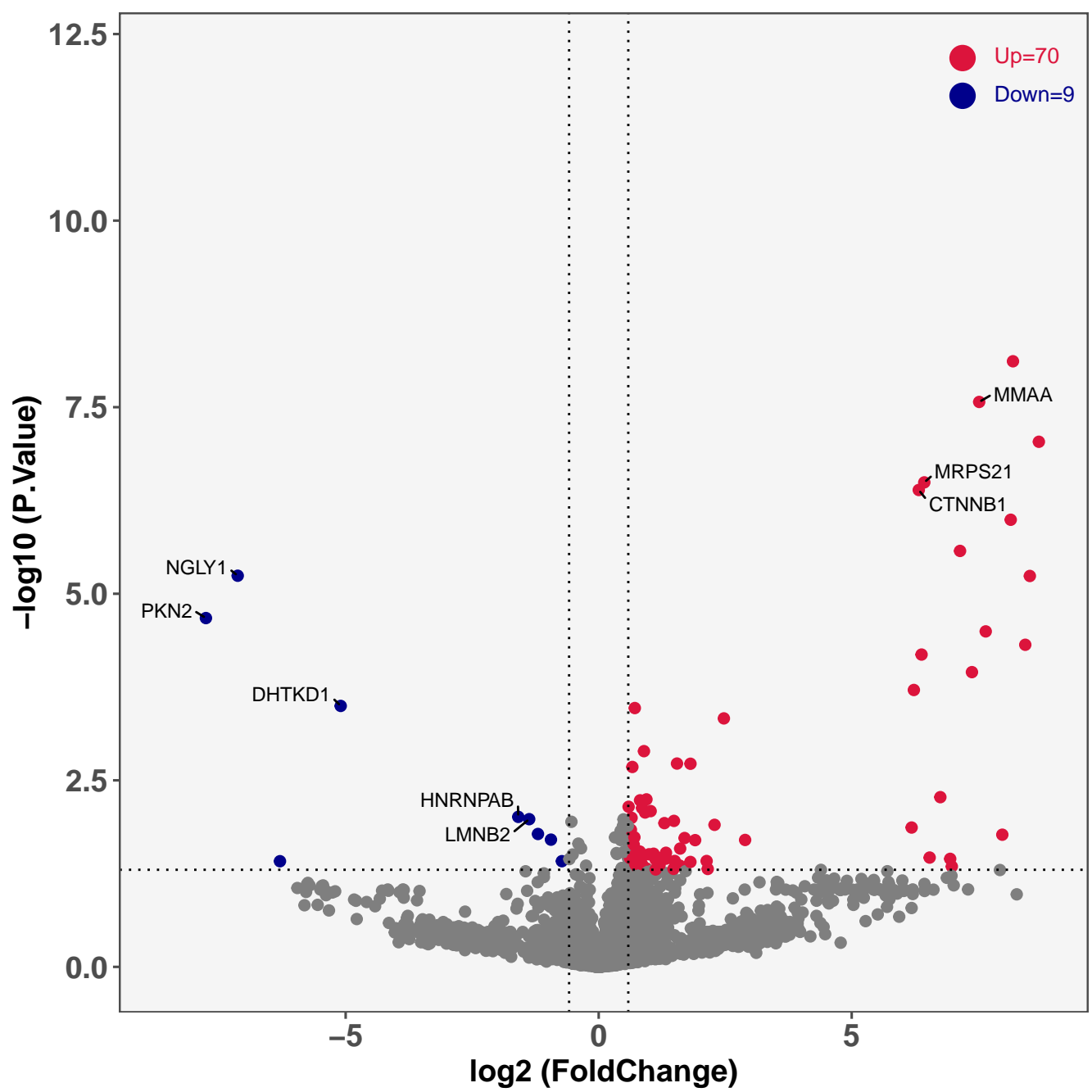

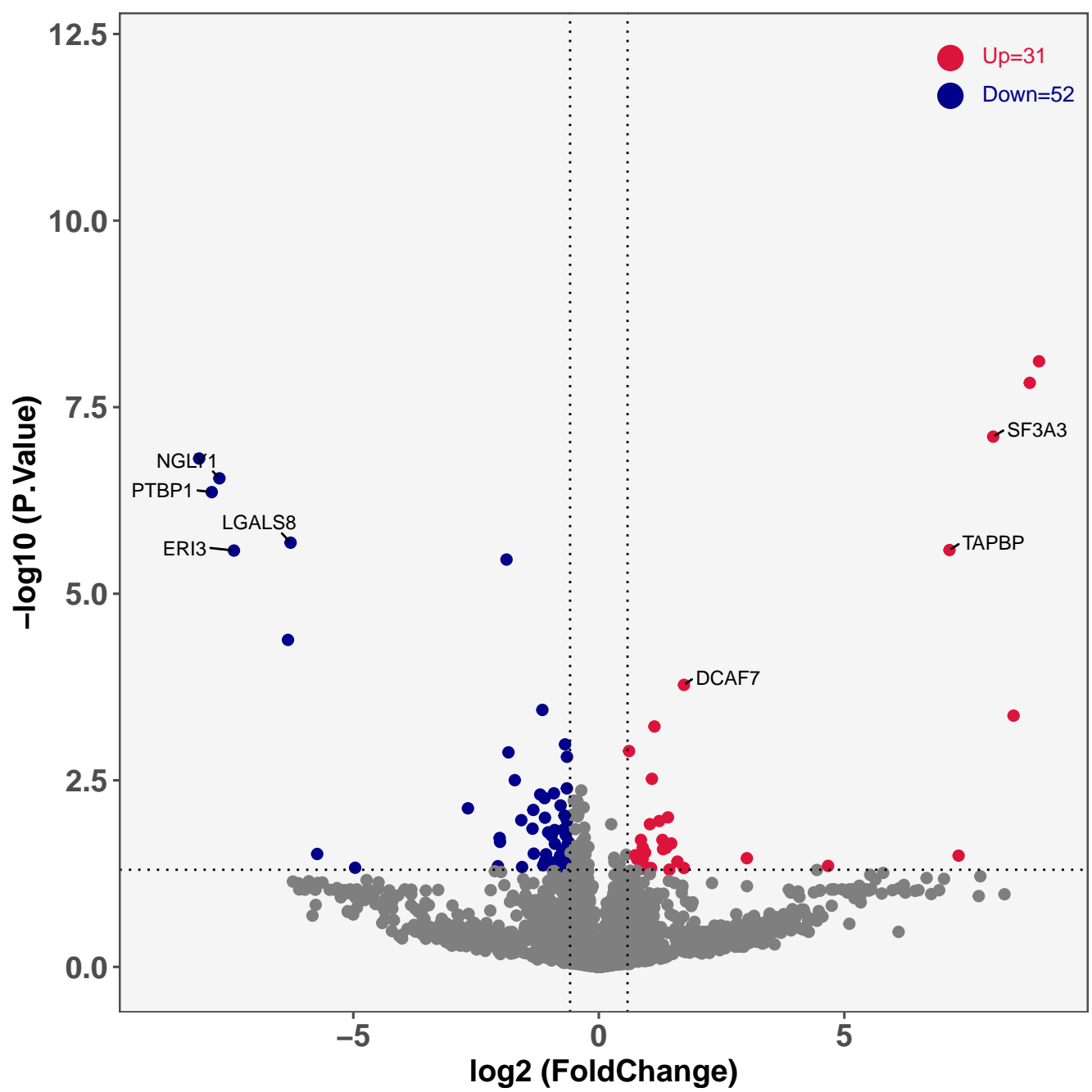

Supplement: SUPPLEMENTARY FIGURE 2 — Volcano_label. [file Image_2.pdf]

Heatmap

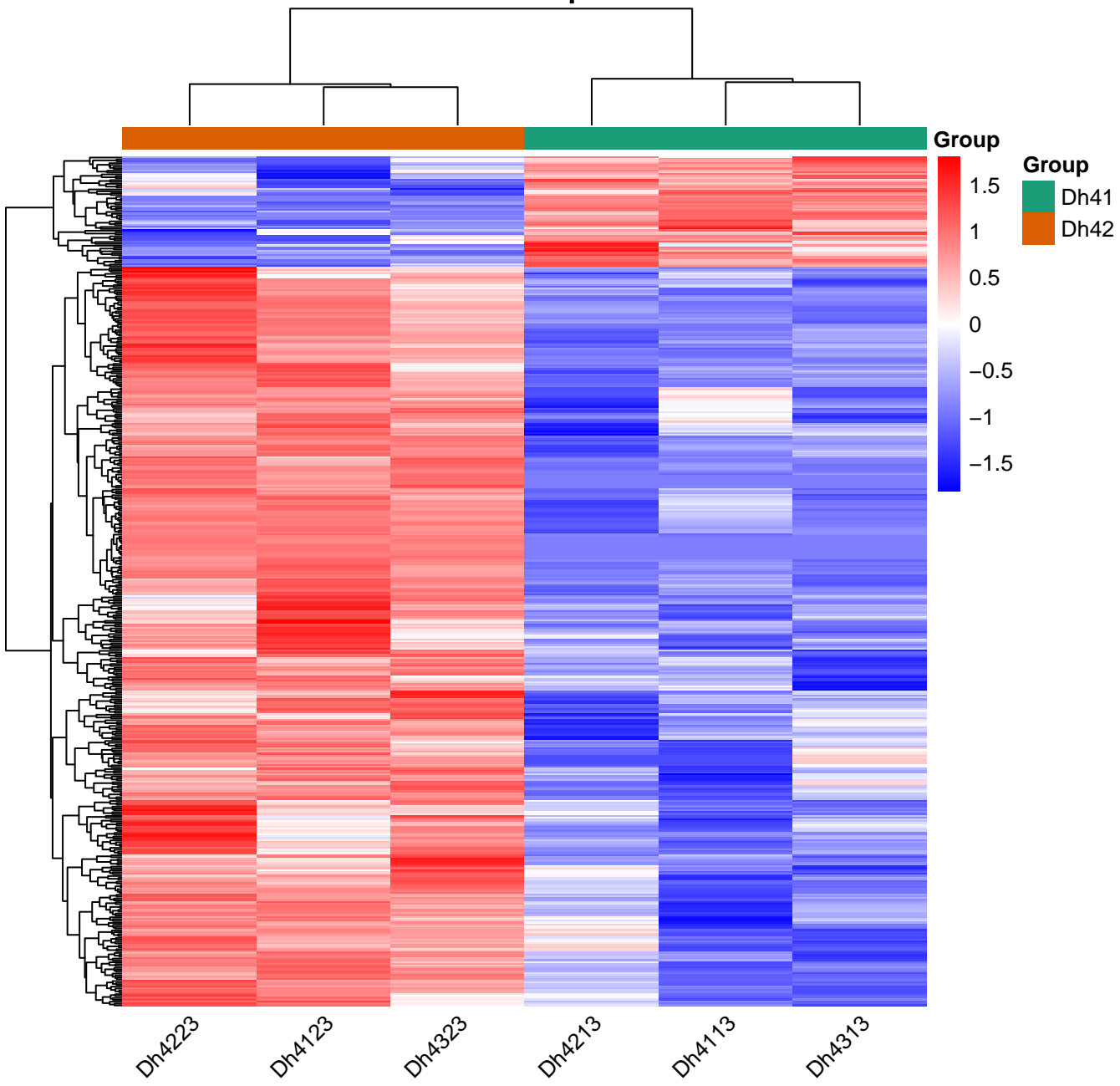

Heatmap

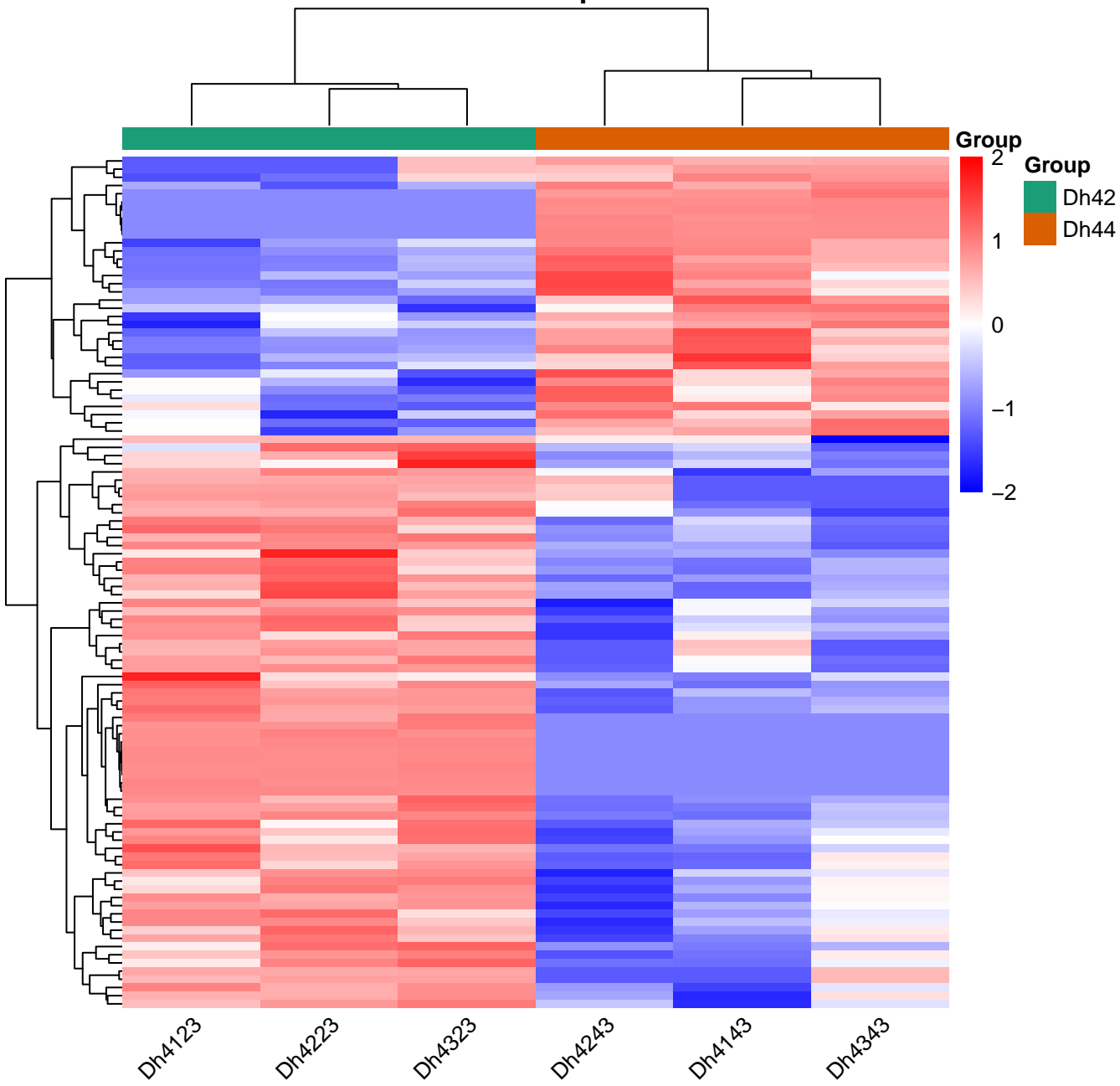

Heatmap

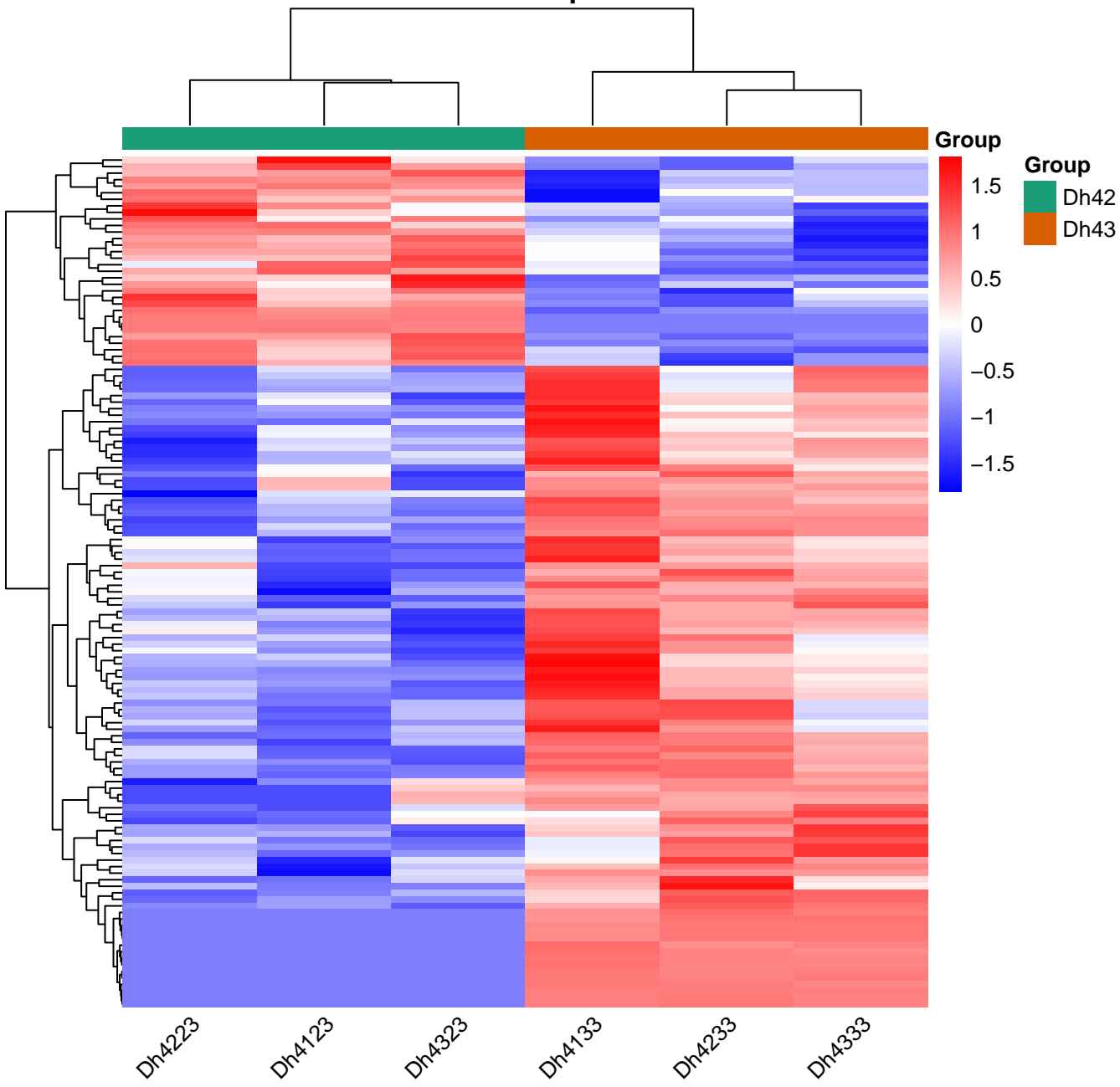

Heatmap

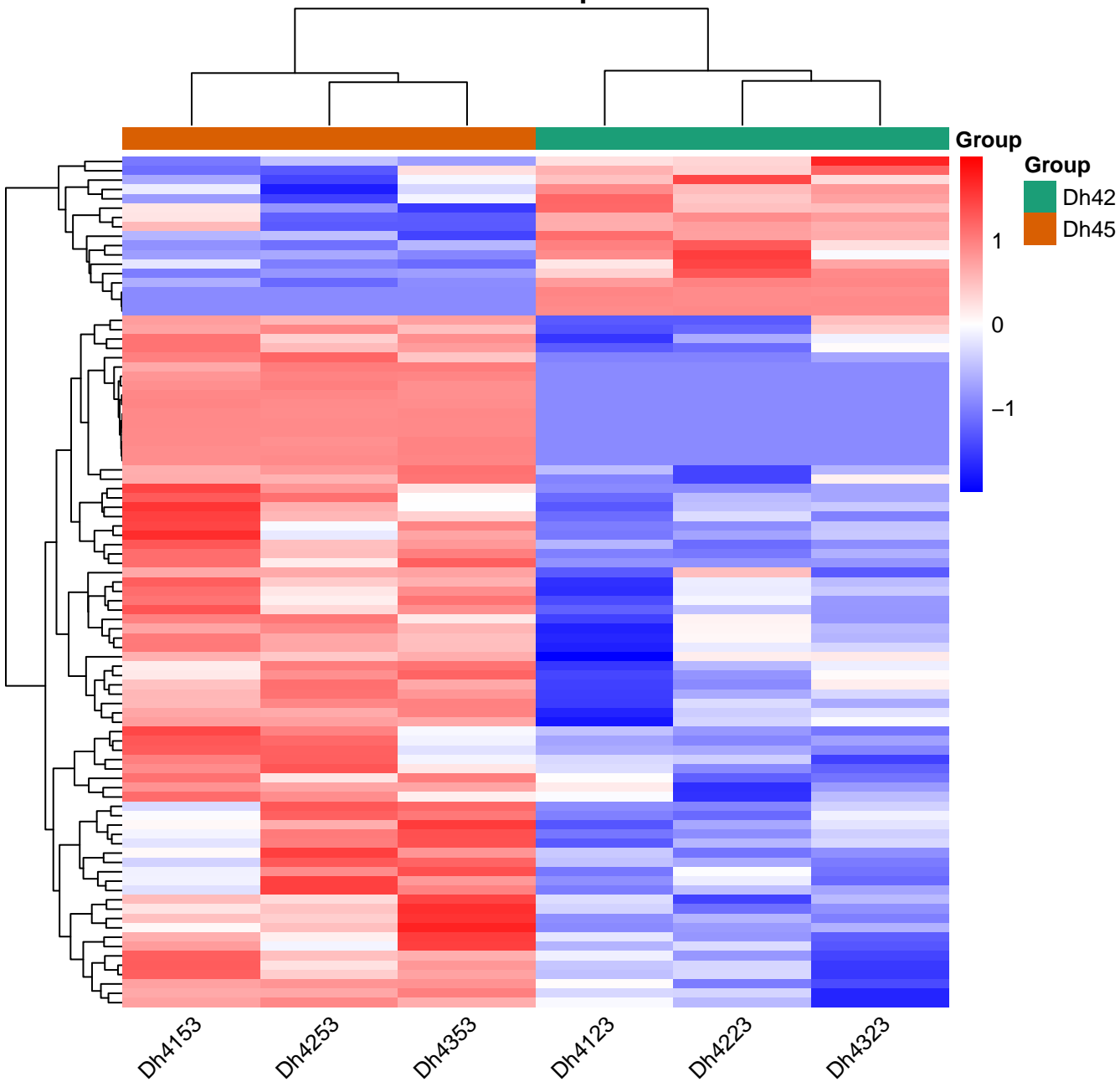

Heatmap

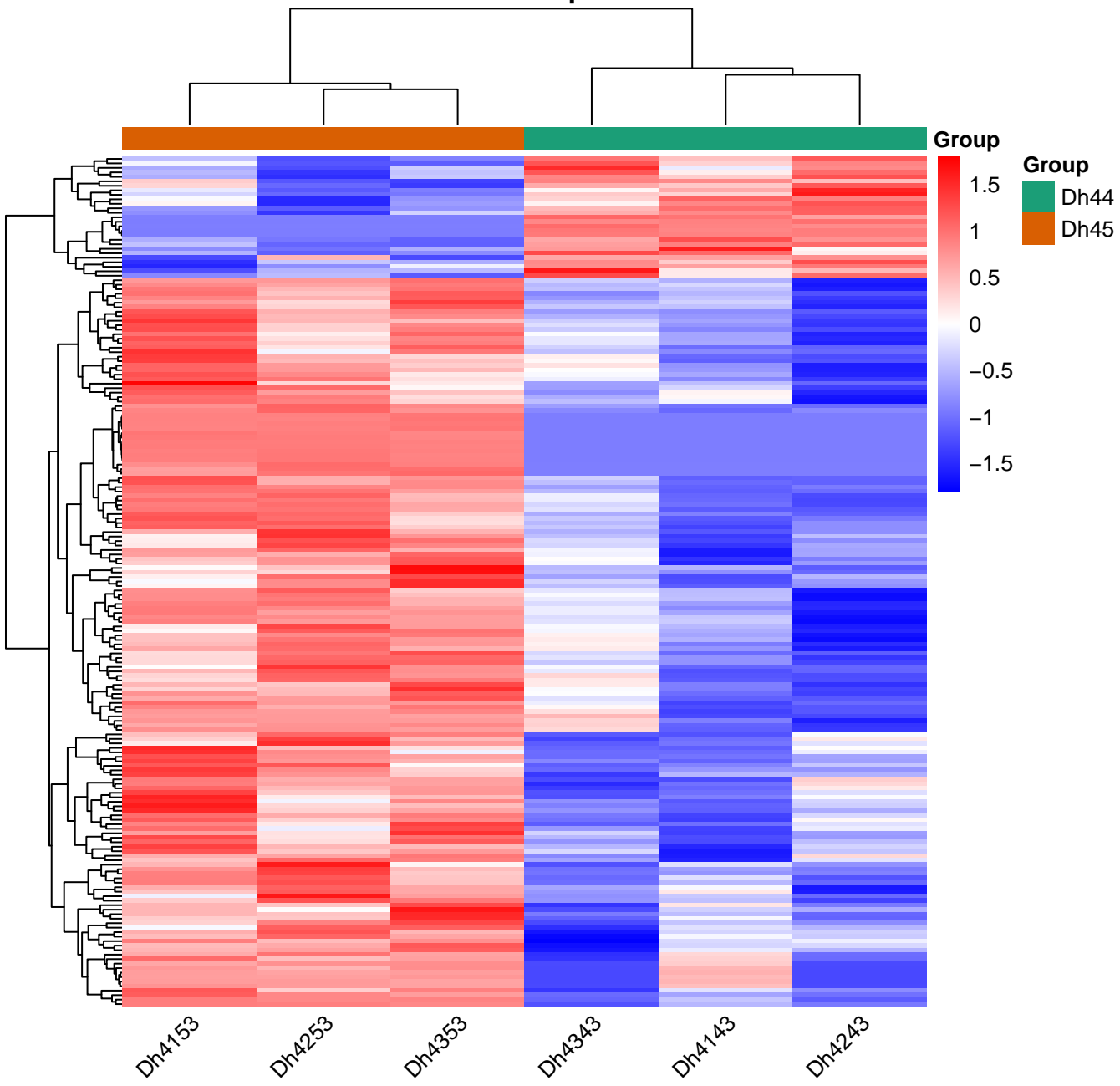

Heatmap

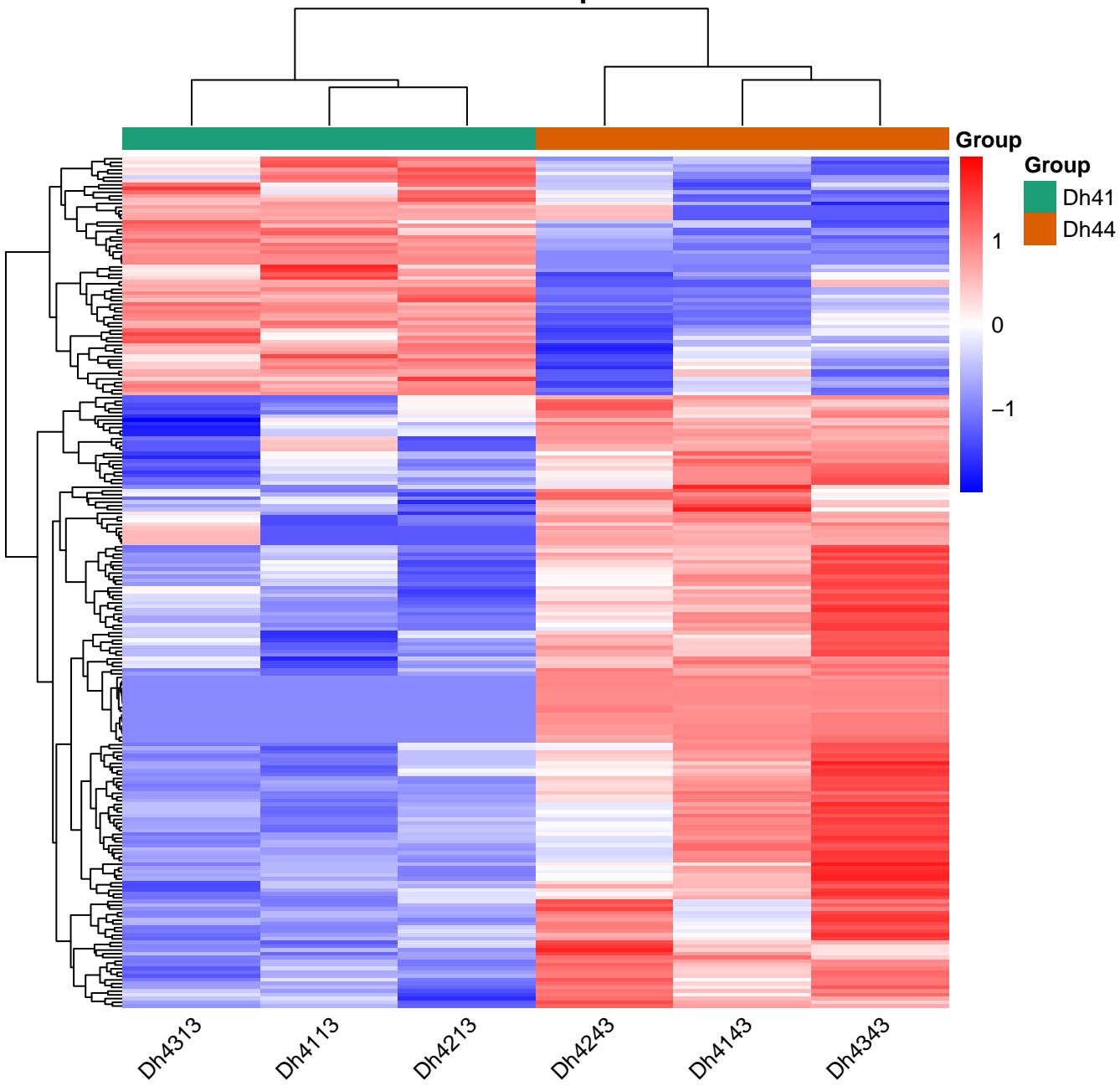

Heatmap

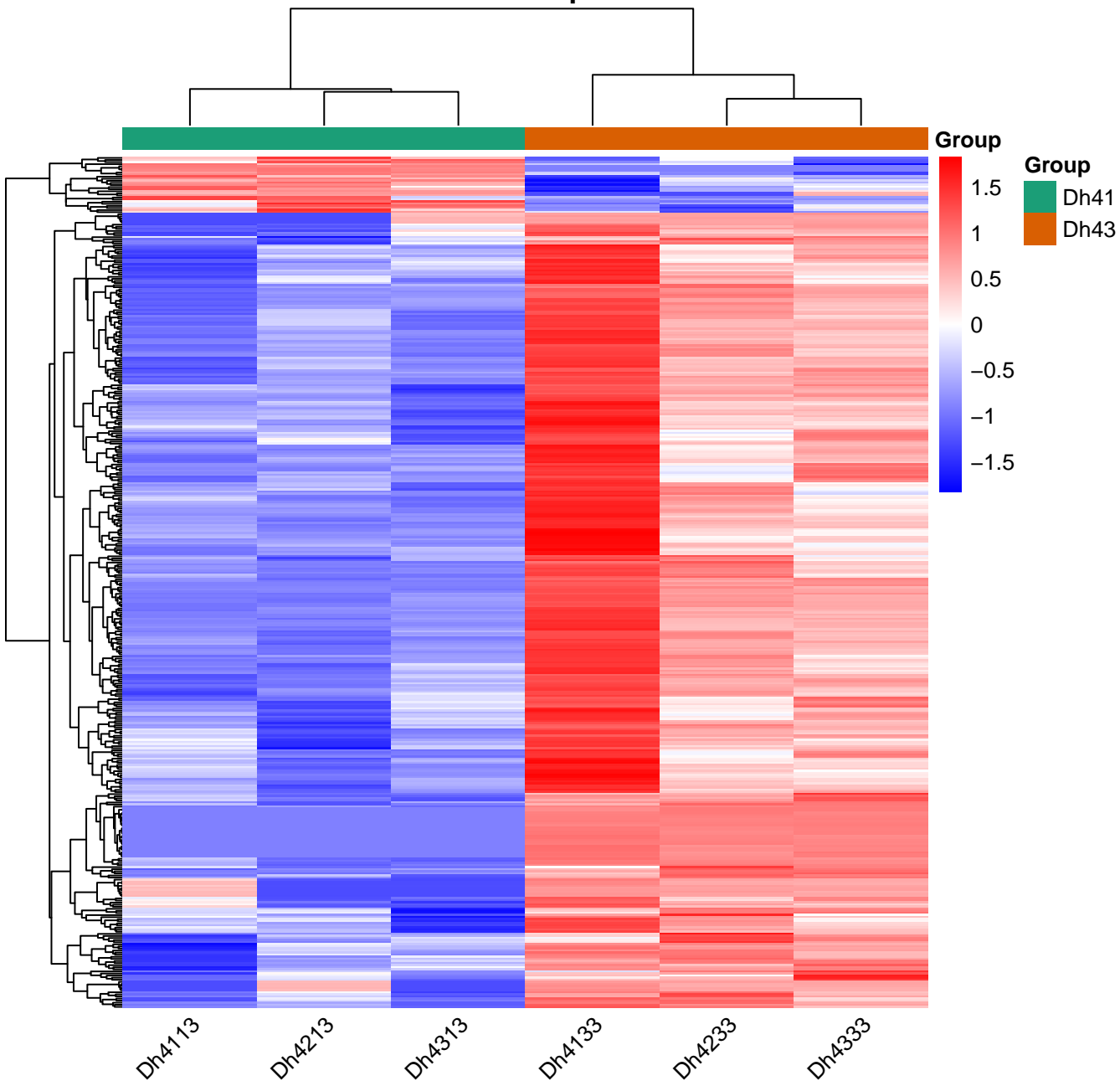

Heatmap

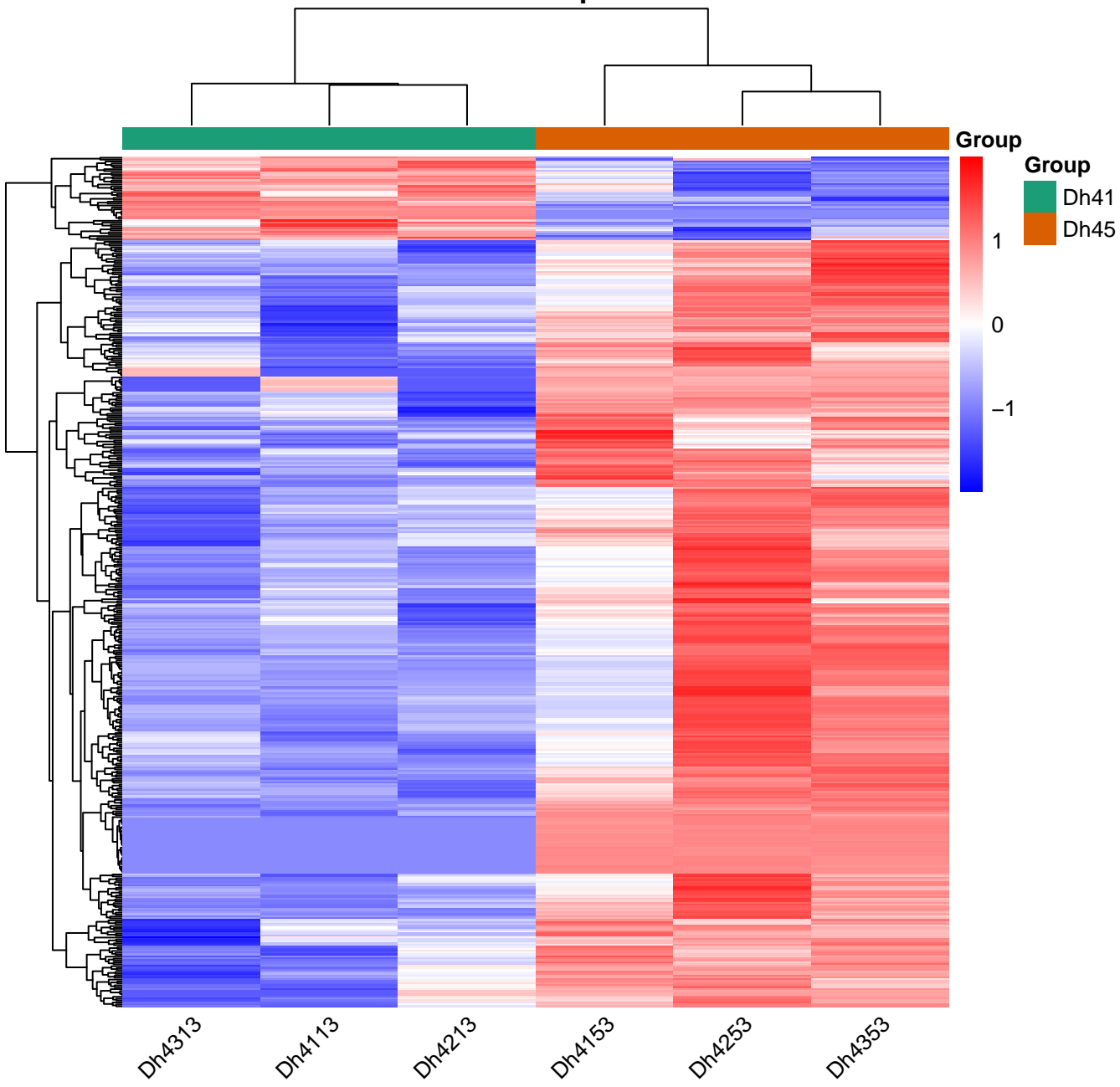

Heatmap

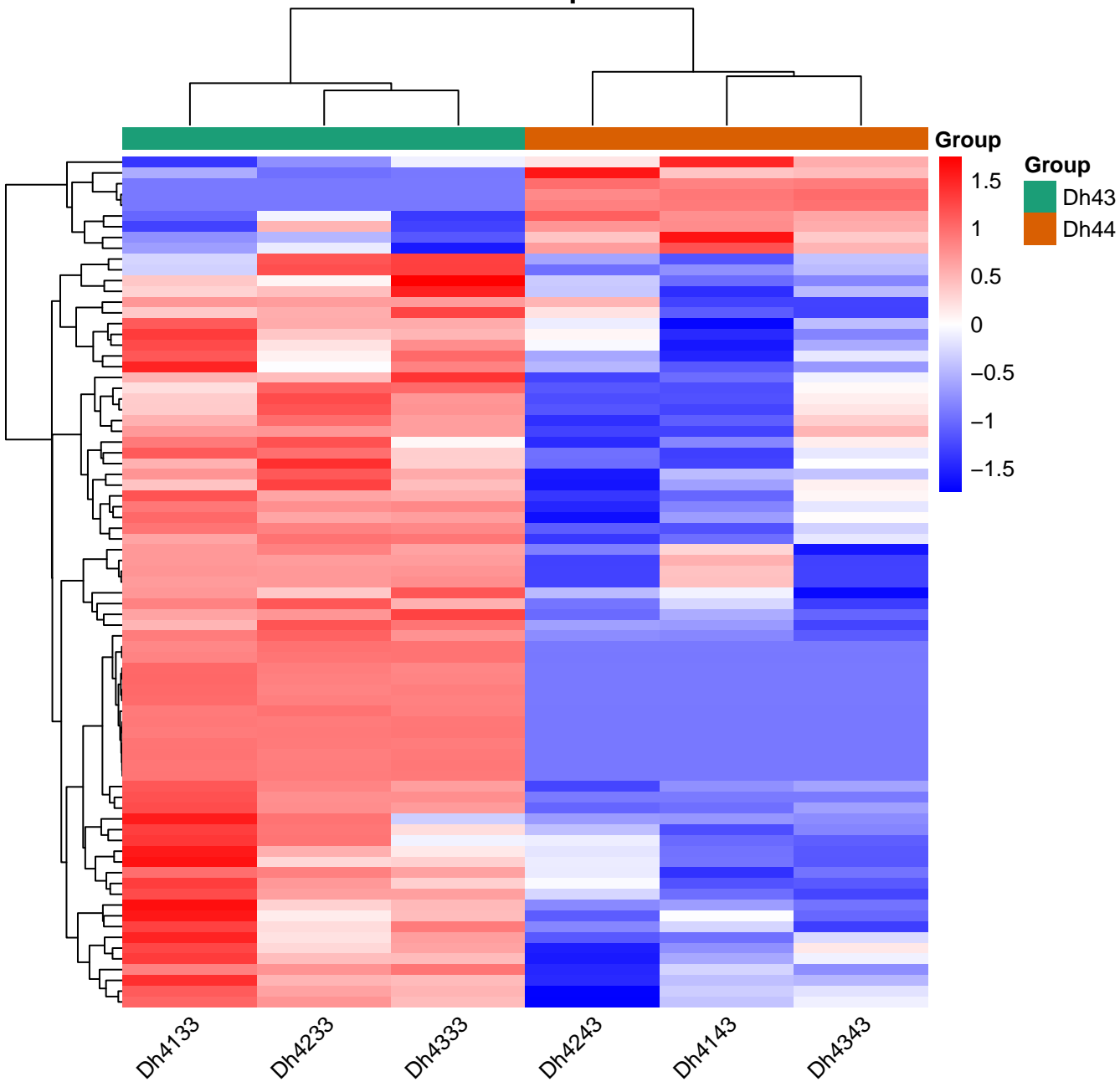

Heatmap

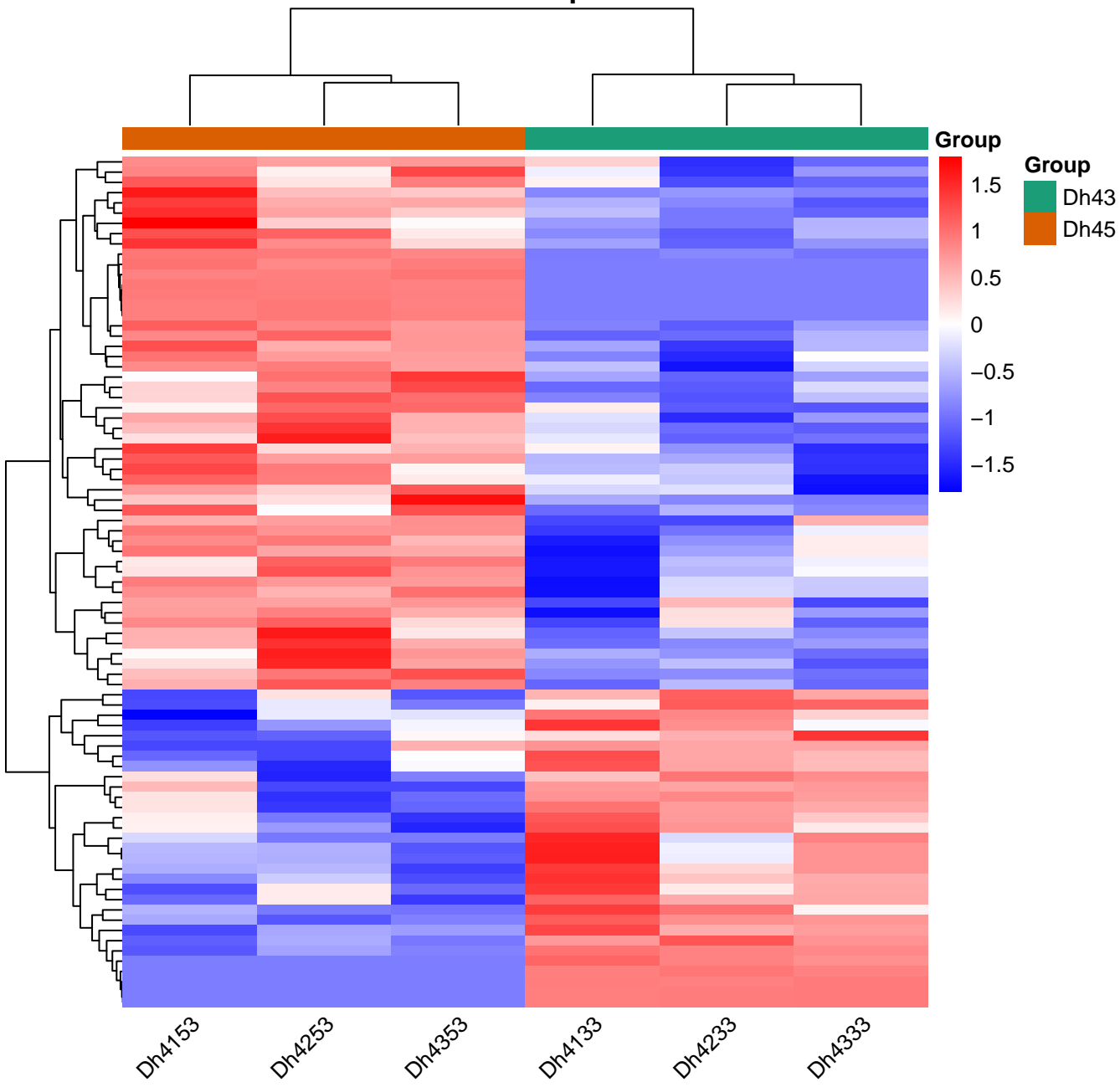

Supplement: SUPPLEMENTARY FIGURE 4 — Heatmap. [file Image_4.pdf]

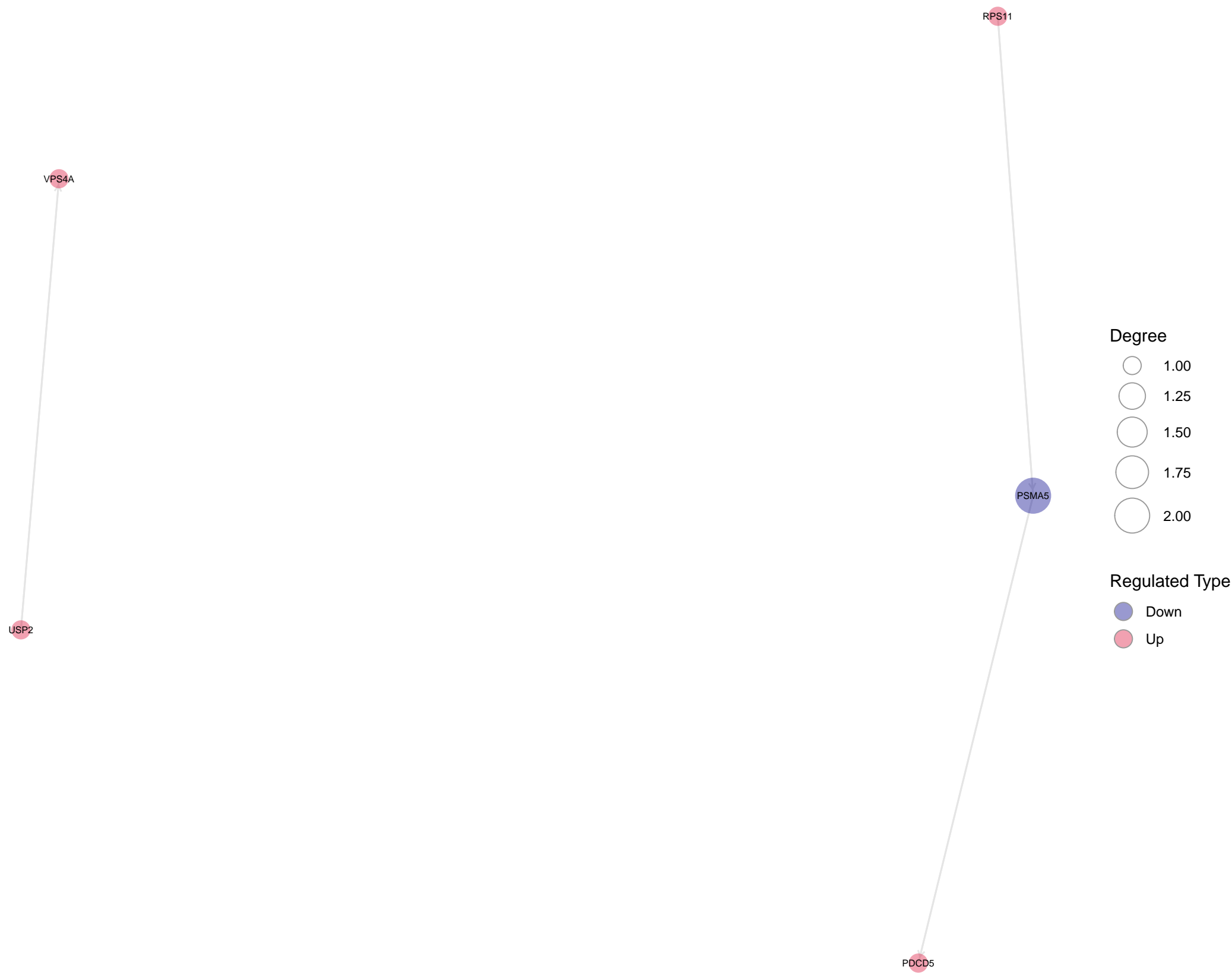

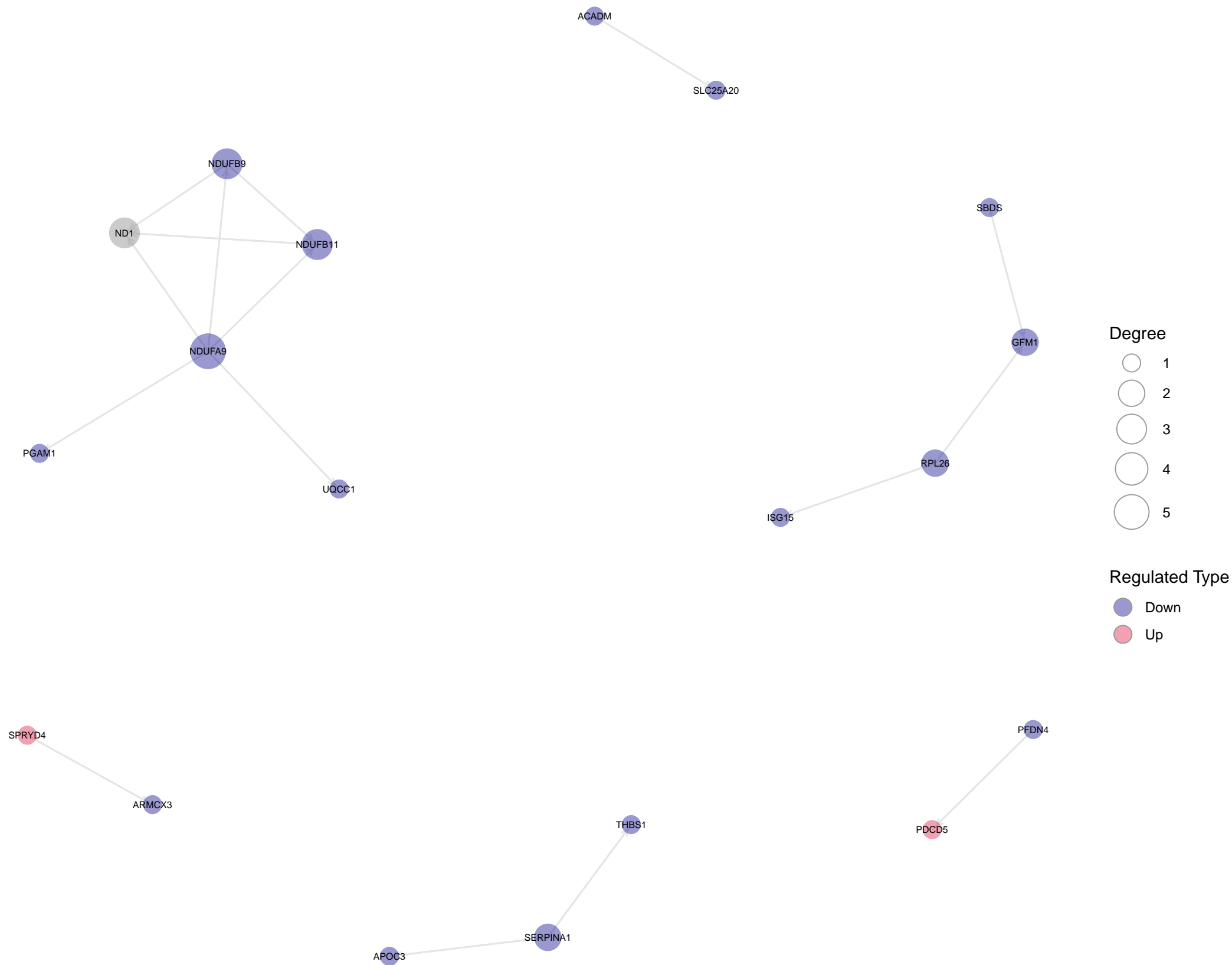

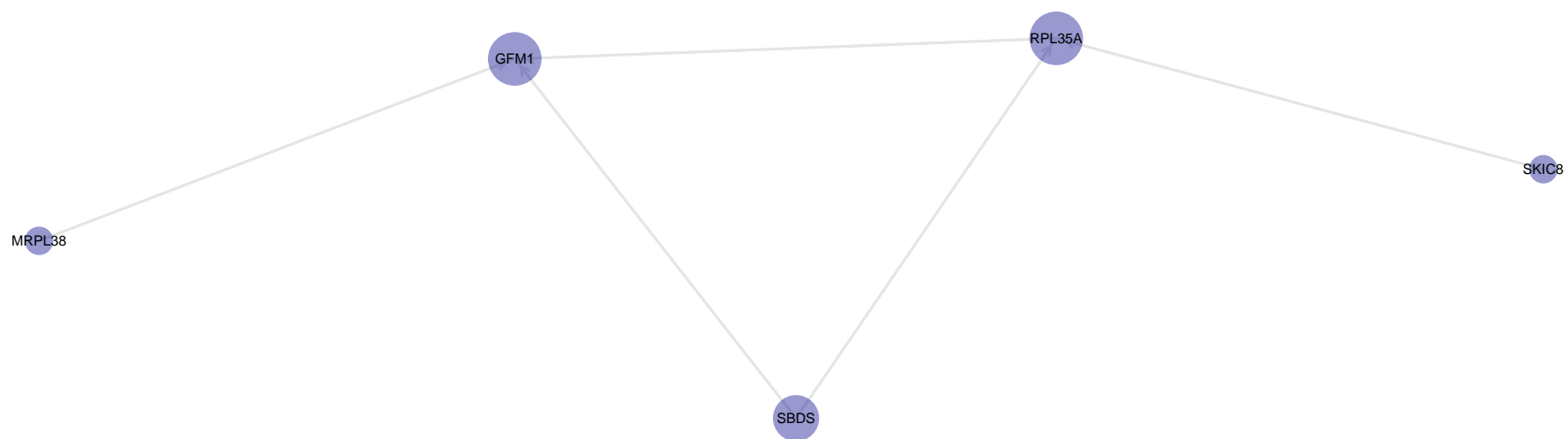

Regulated Type

Down

Degree

1.0

1.5

2.0

2.5

3.0

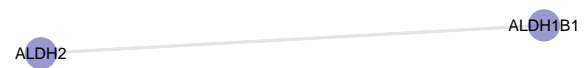

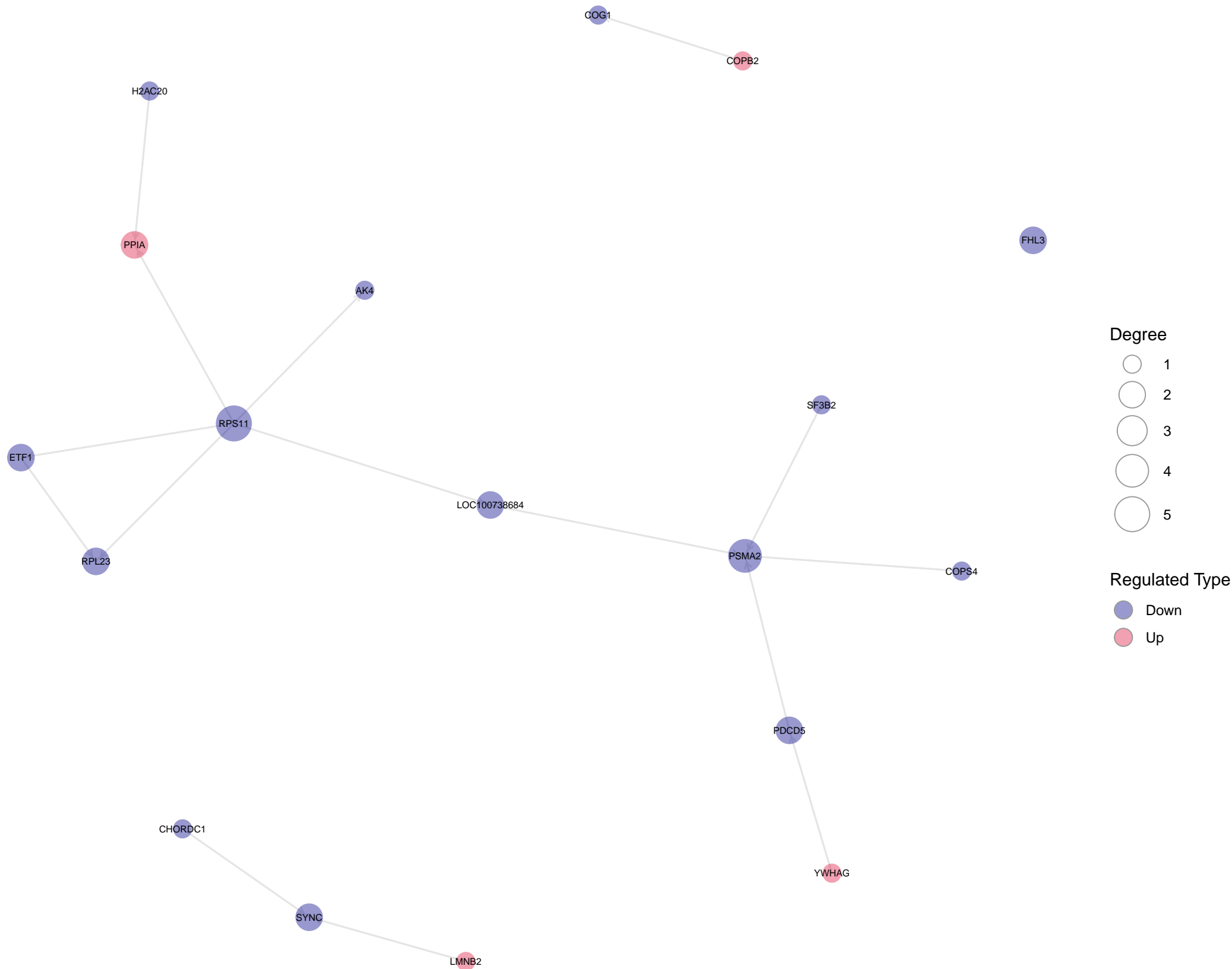

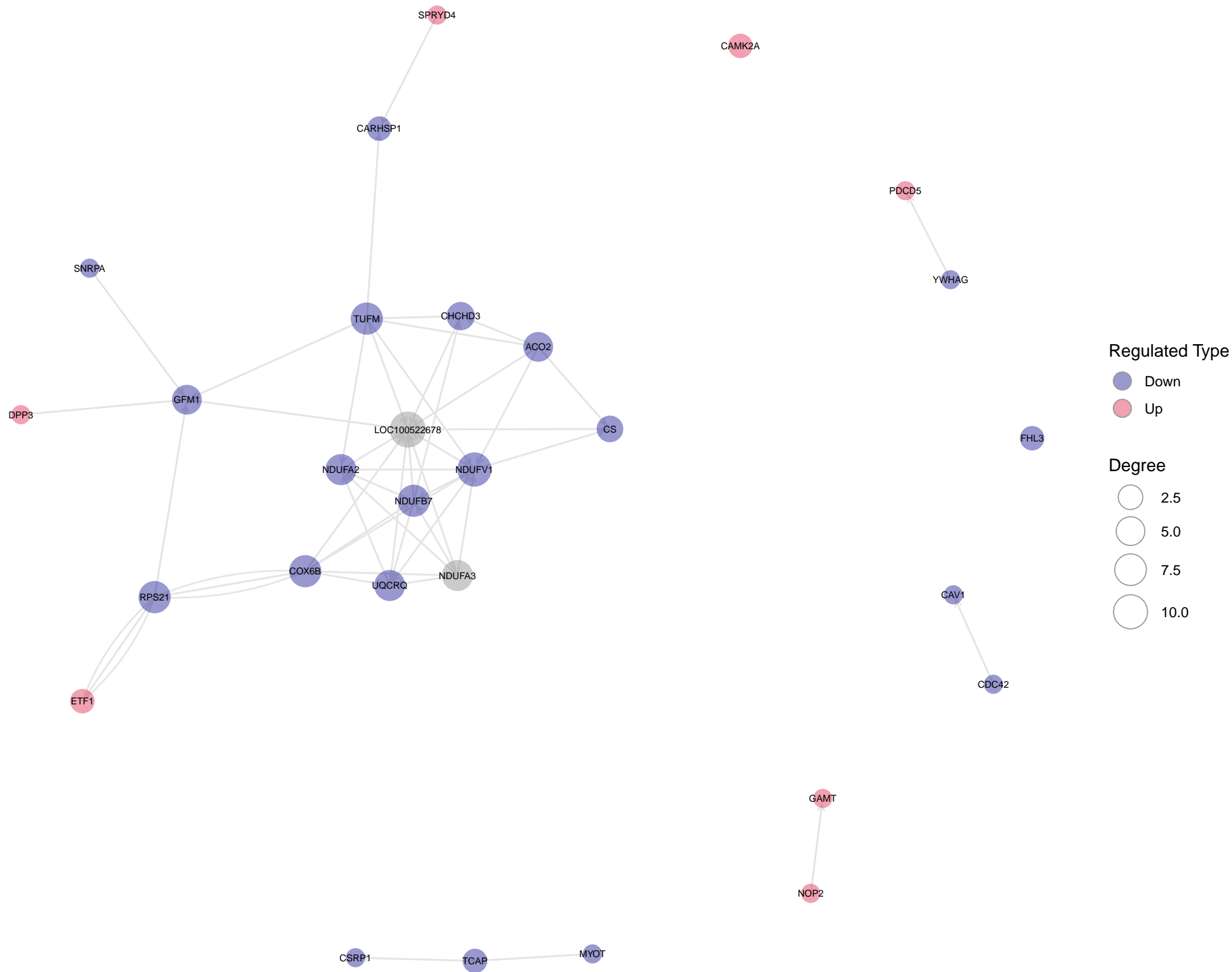

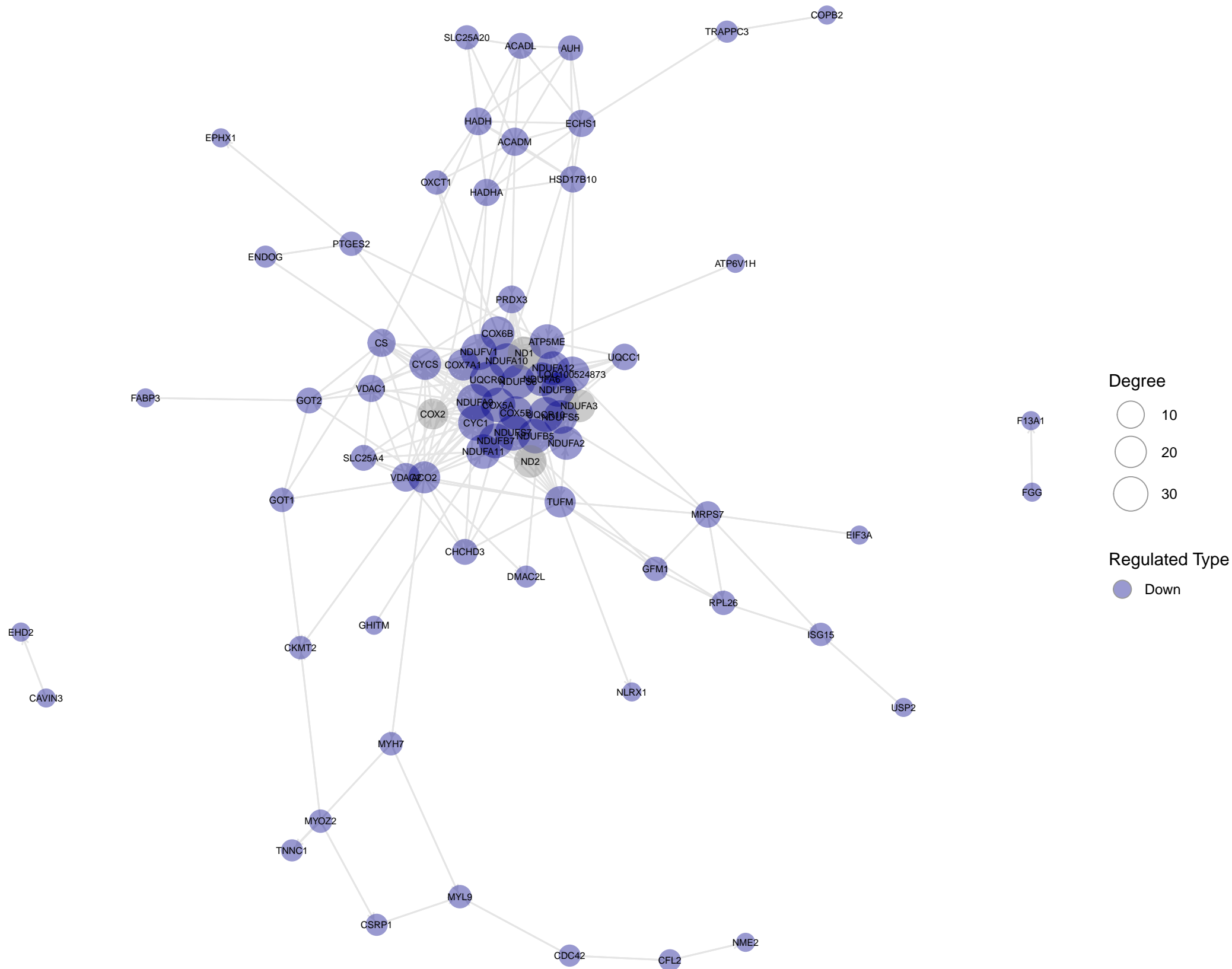

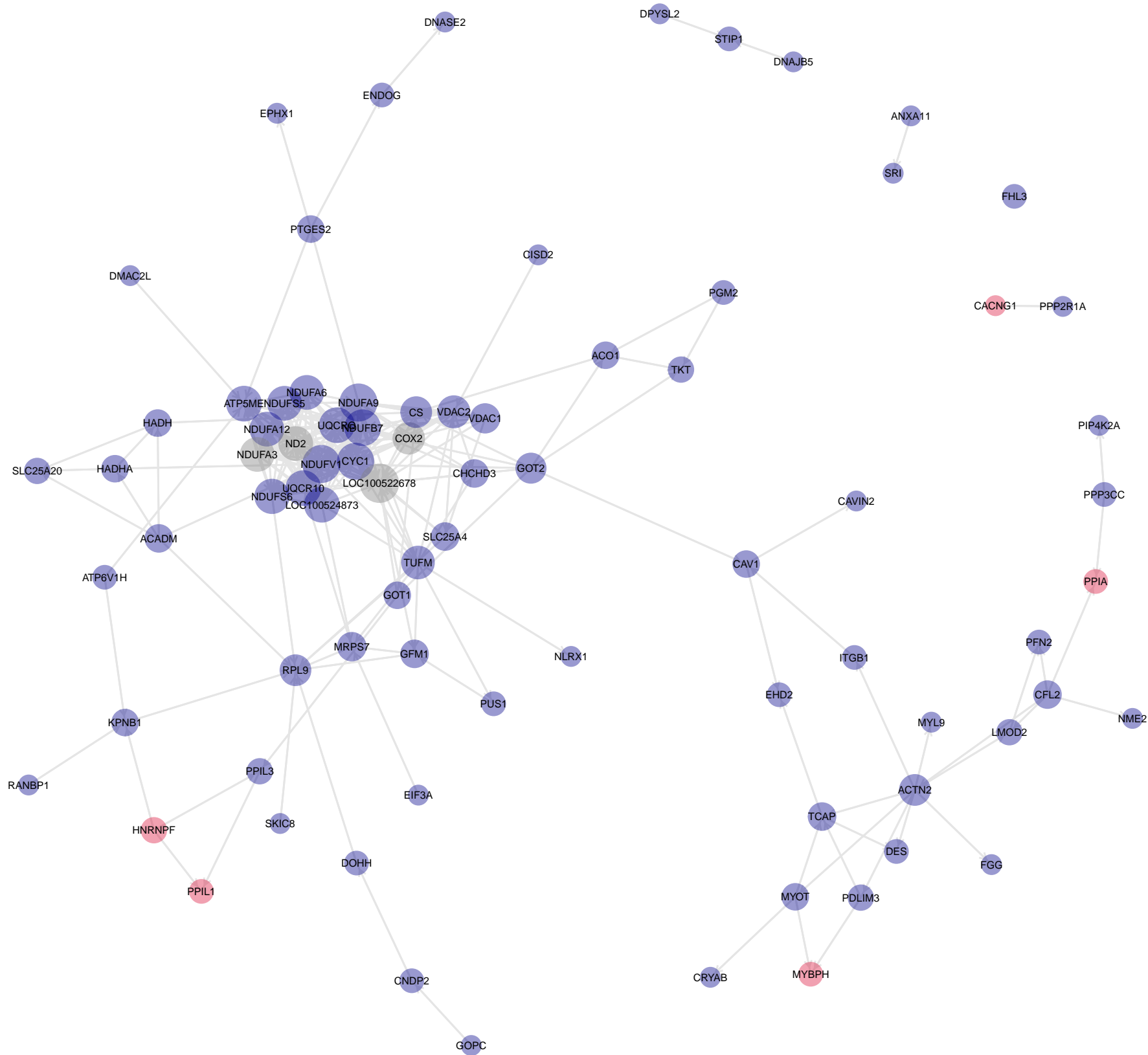

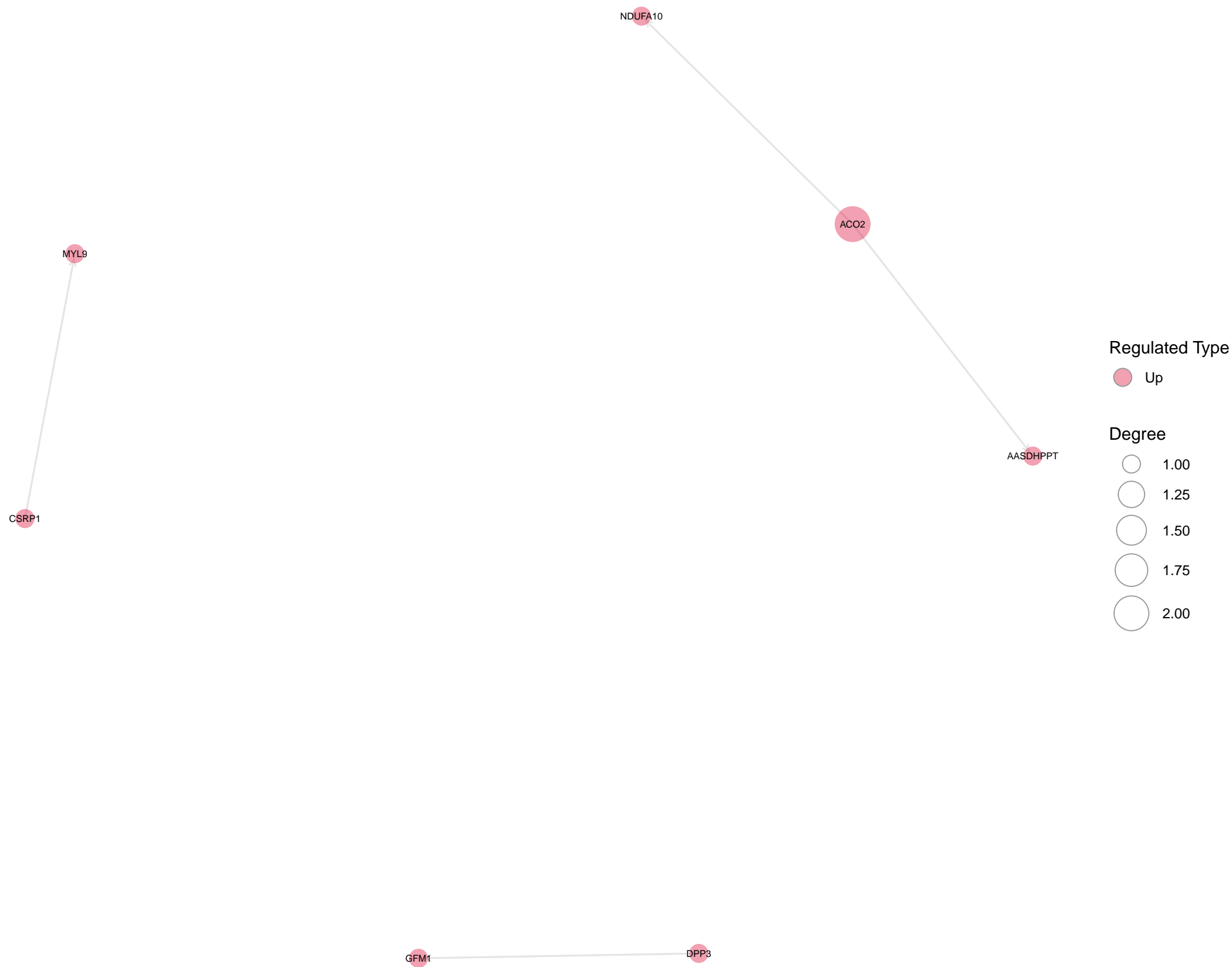

CNDP2

CARNMT1

Regulated Type

- Down
- Up

Degree

1

Supplement: SUPPLEMENTARY FIGURE 7 — Differential protein interaction network diagram (PPI). [file Image_7.pdf]
