# Supplementary material for: Integrative analysis of proteomics and lipidomic profiles reveal the fat deposition and meat quality in Duroc × Guangdong small spotted pig
Source: Front Vet Sci. 2024 Apr 10;11:1361441. doi: 10.3389/fvets.2024.1361441 (PMC11041638; doi:10.3389/fvets.2024.1361441)

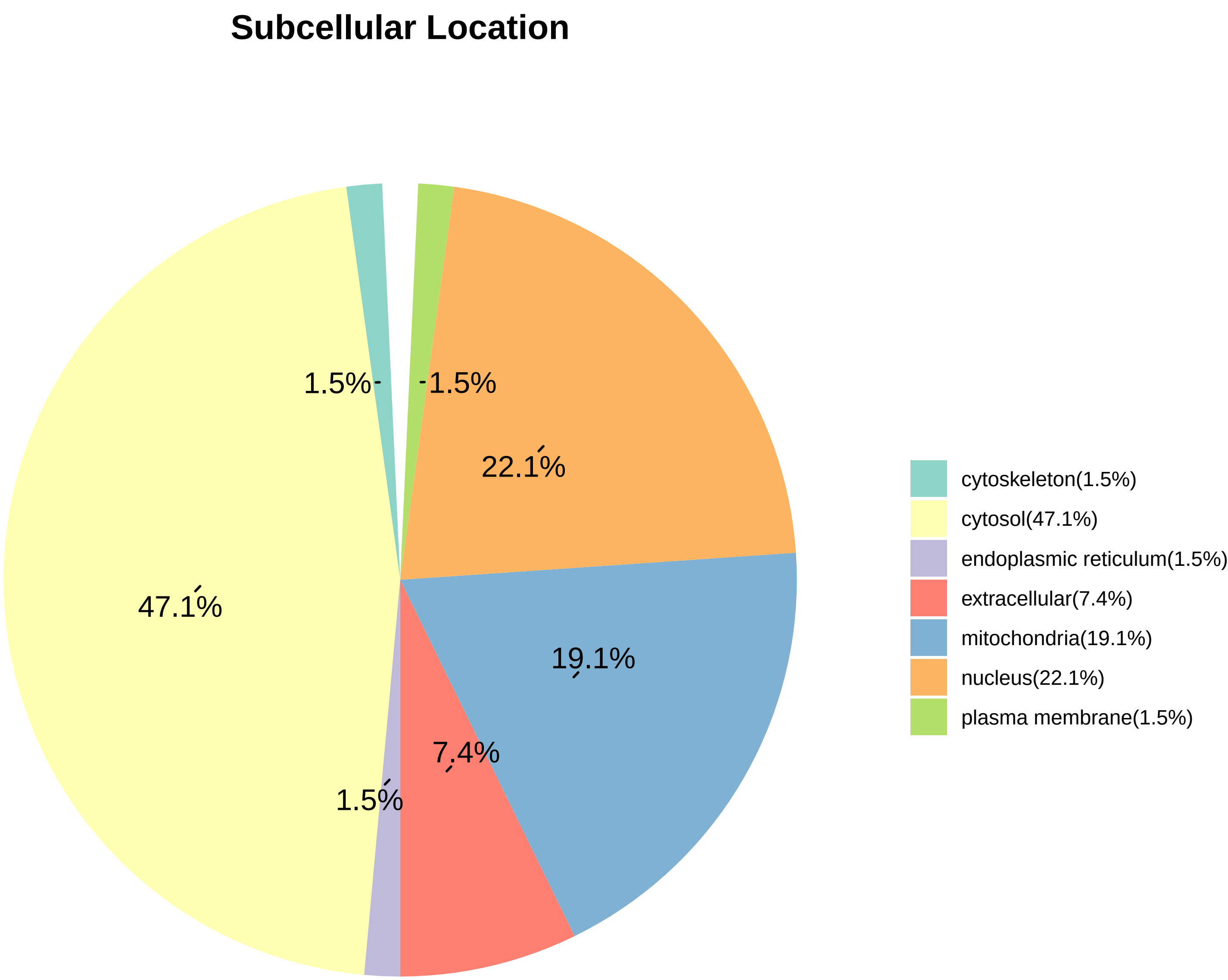

Subcellular Location
1.5%
1.5%
22.1%
47.1%
19.1%
7.4%
1.5%
cytoskeleton(1.5%)
cytosol(47.1%)
endoplasmic reticulum(1.5%)
extracellular(7.4%)
mitochondria(19.1%)
nucleus(22.1%)
plasma membrane(1.5%)

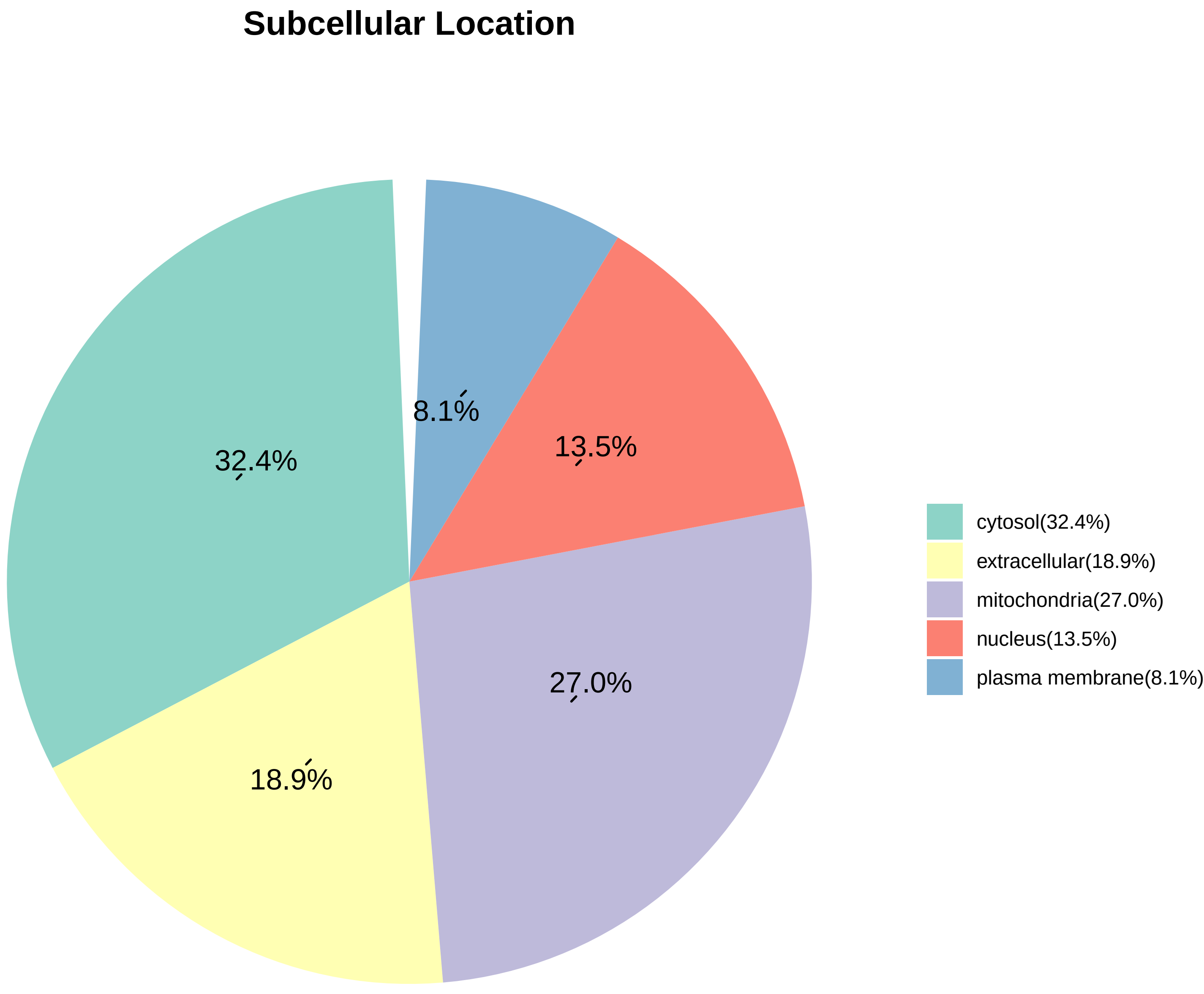
Subcellular Location
32.4%
8.1%
13.5%
27.0%
18.9%
cytosol(32.4%)
extracellular(18.9%)
mitochondria(27.0%)
nucleus(13.5%)
plasma membrane(8.1%)

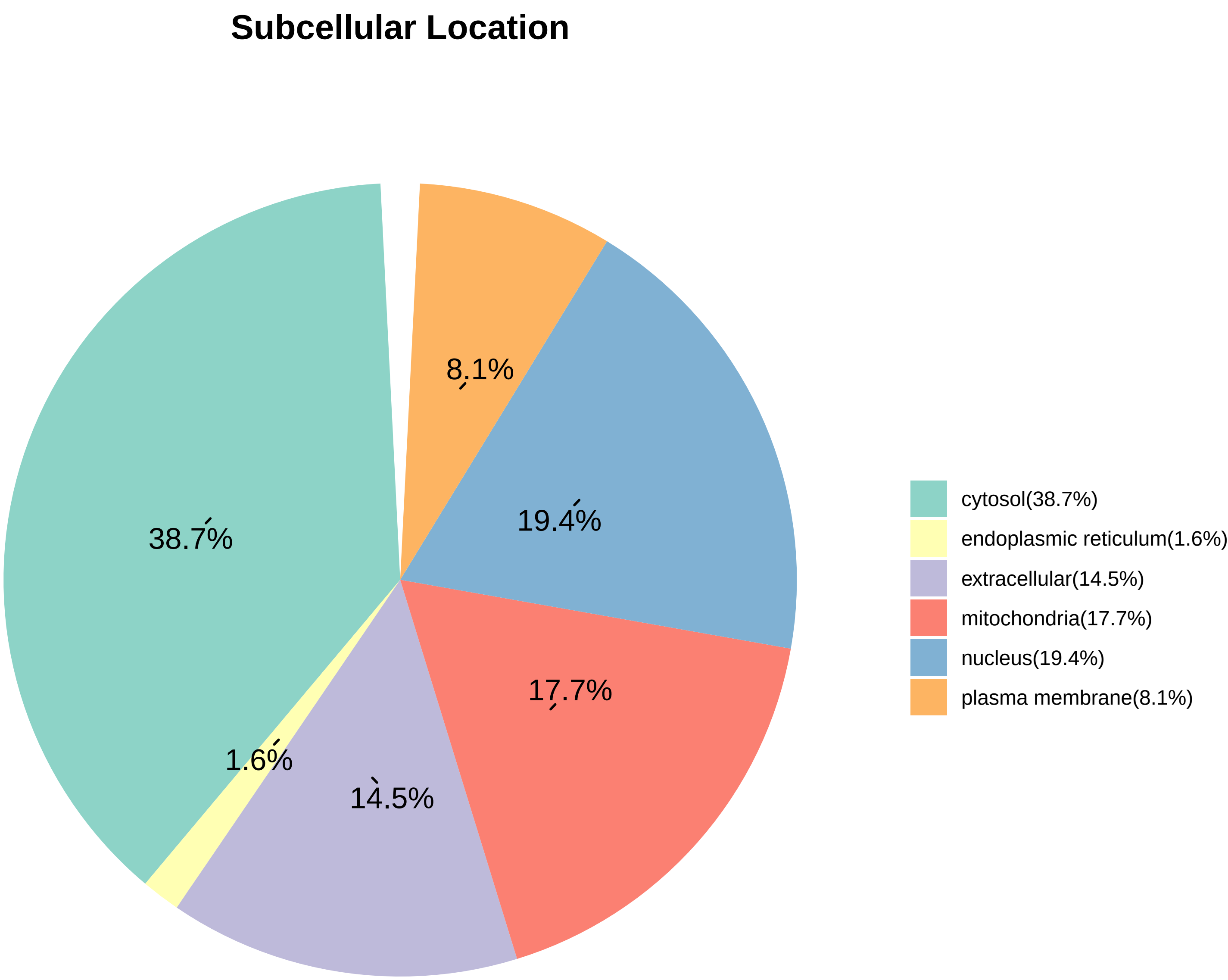

Subcellular Location
38.7%
1.6%
14.5%
17.7%
19.4%
8.1%
cytosol(38.7%)
endoplasmic reticulum(1.6%)
extracellular(14.5%)
mitochondria(17.7%)
nucleus(19.4%)
plasma membrane(8.1%)

Subcellular Location

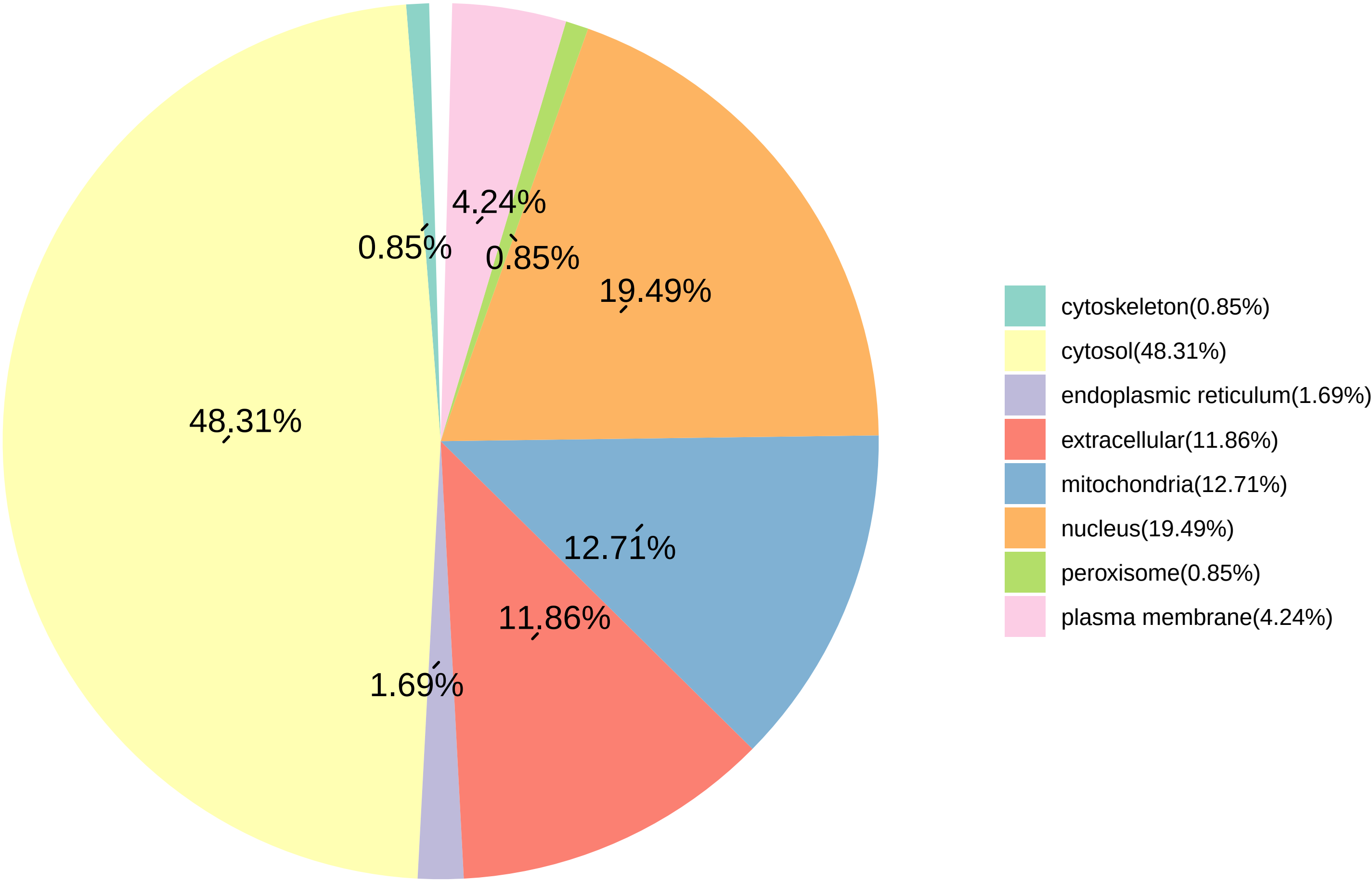

Subcellular Location

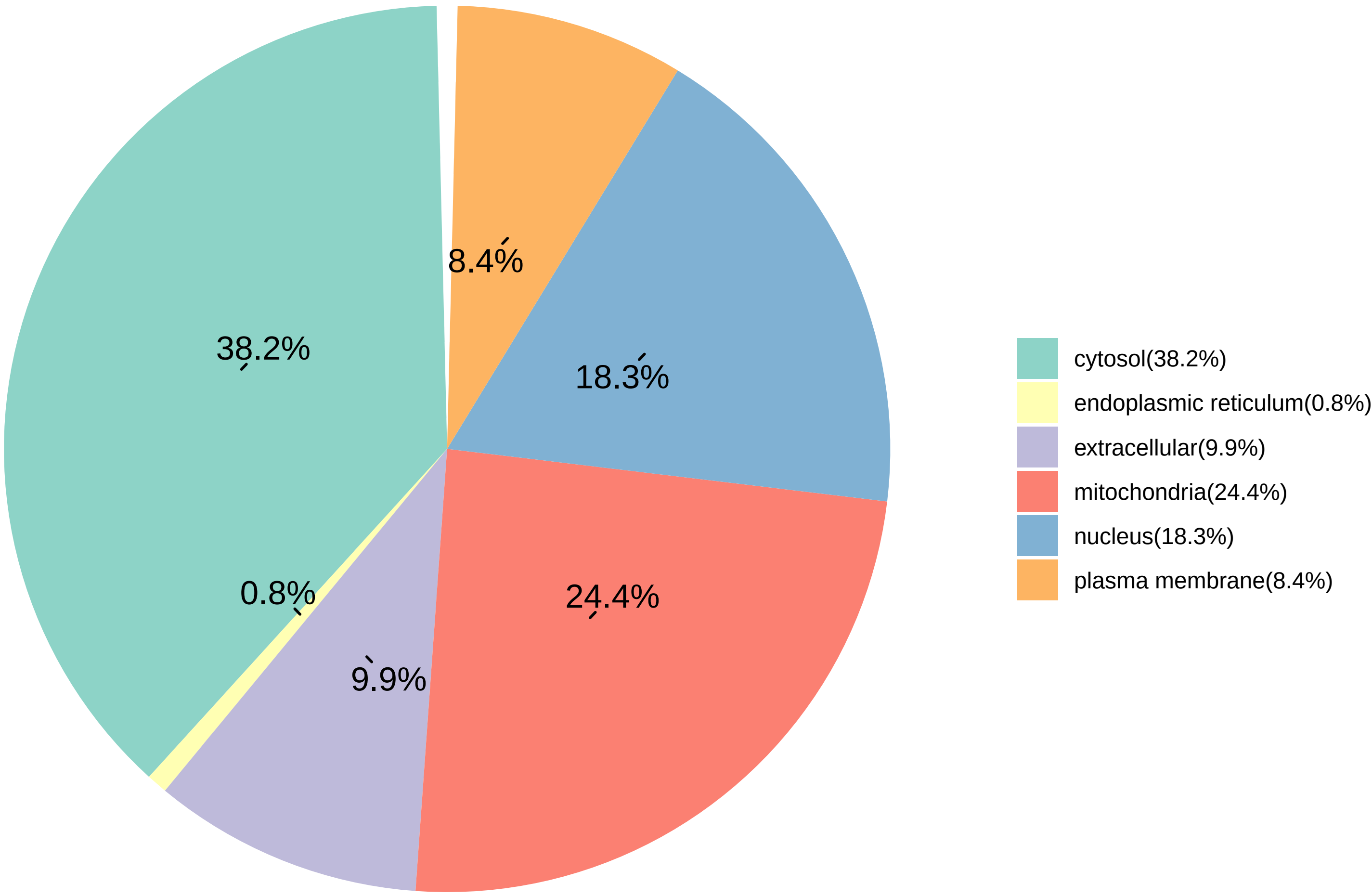

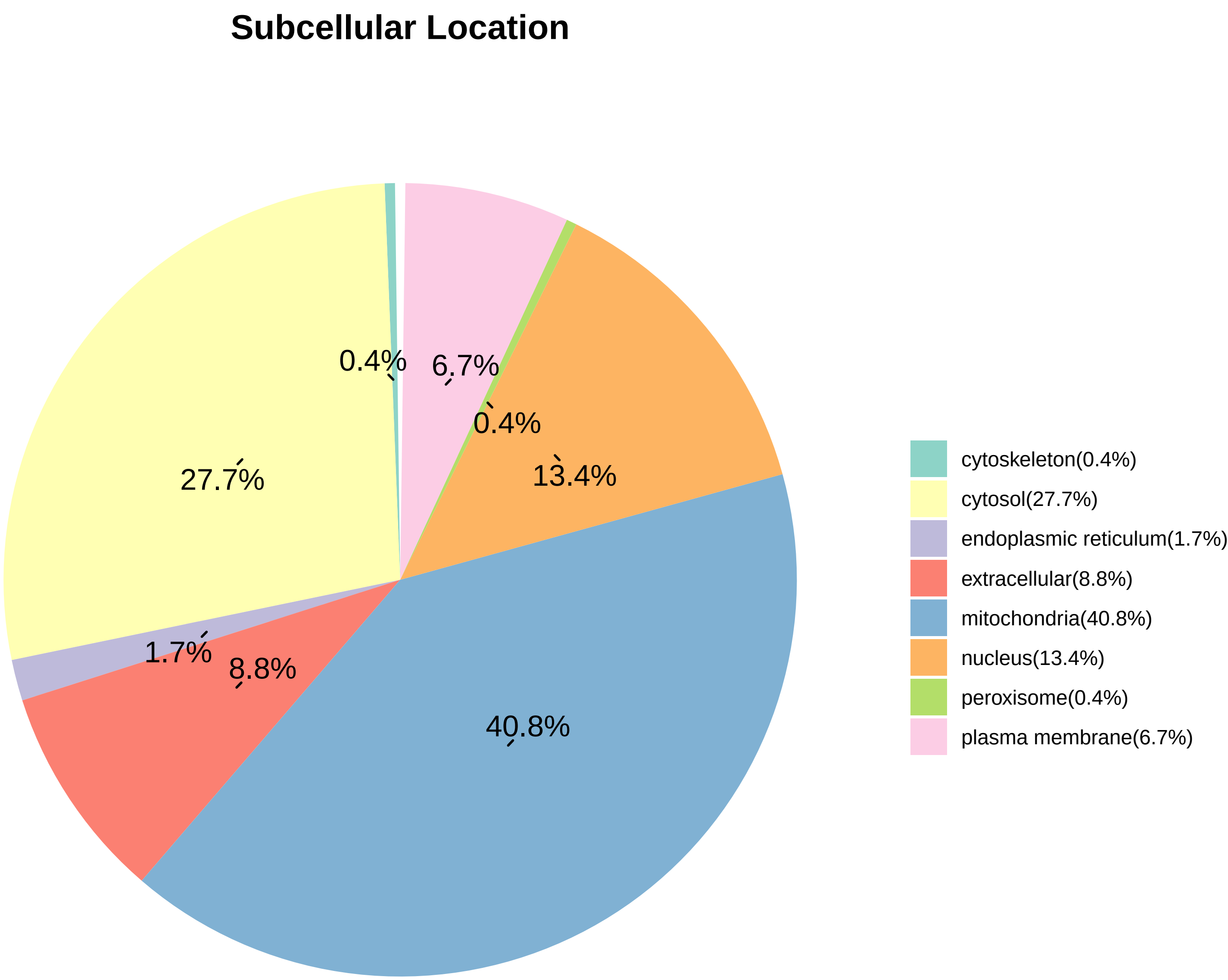
Subcellular Location
0.4%
6.7%
0.4%
13.4%
27.7%
1.7%
8.8%
40.8%
cytoskeleton(0.4%)
cytosol(27.7%)
endoplasmic reticulum(1.7%)
extracellular(8.8%)
mitochondria(40.8%)
nucleus(13.4%)
peroxisome(0.4%)
plasma membrane(6.7%)

Subcellular Location

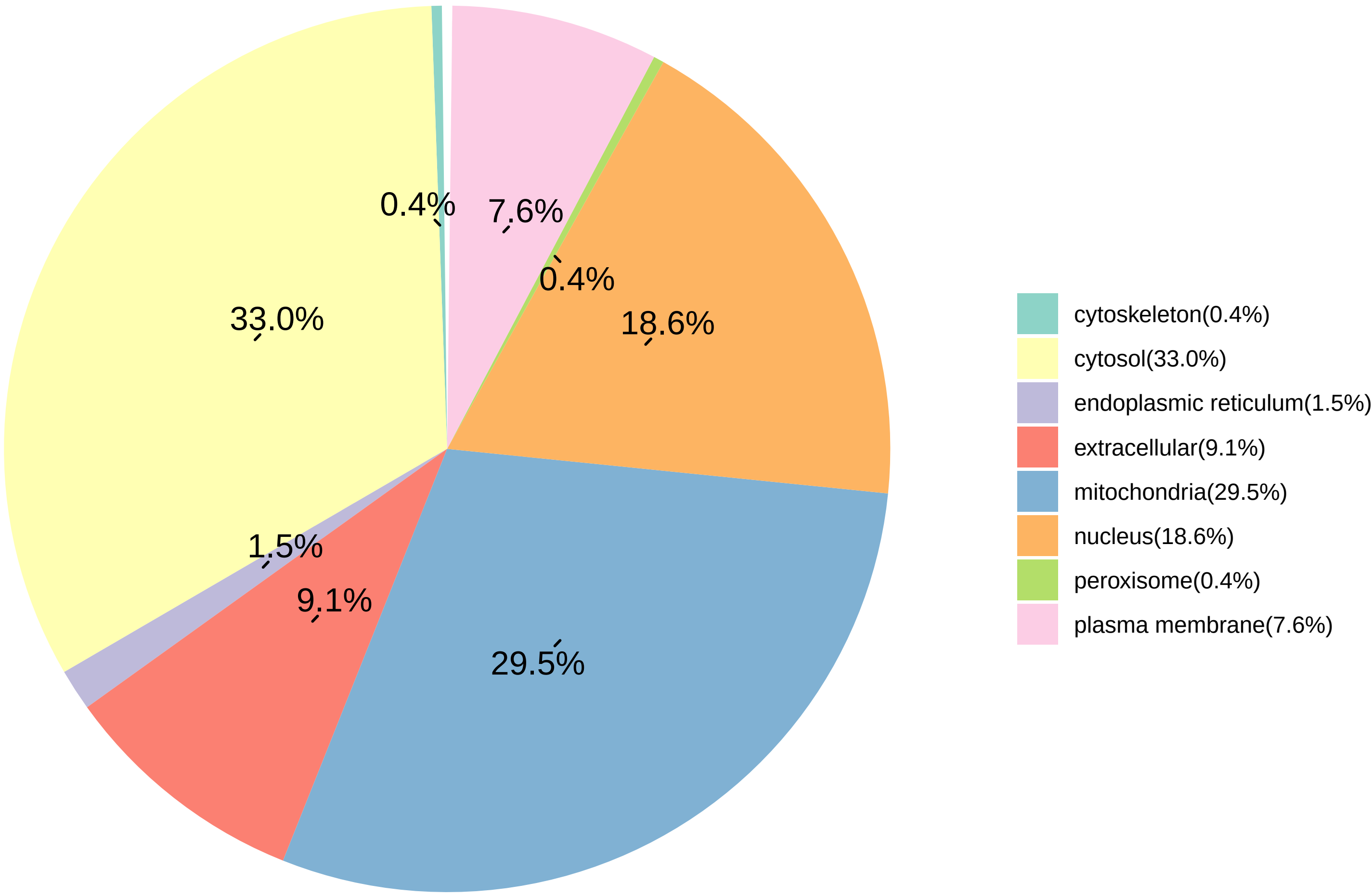

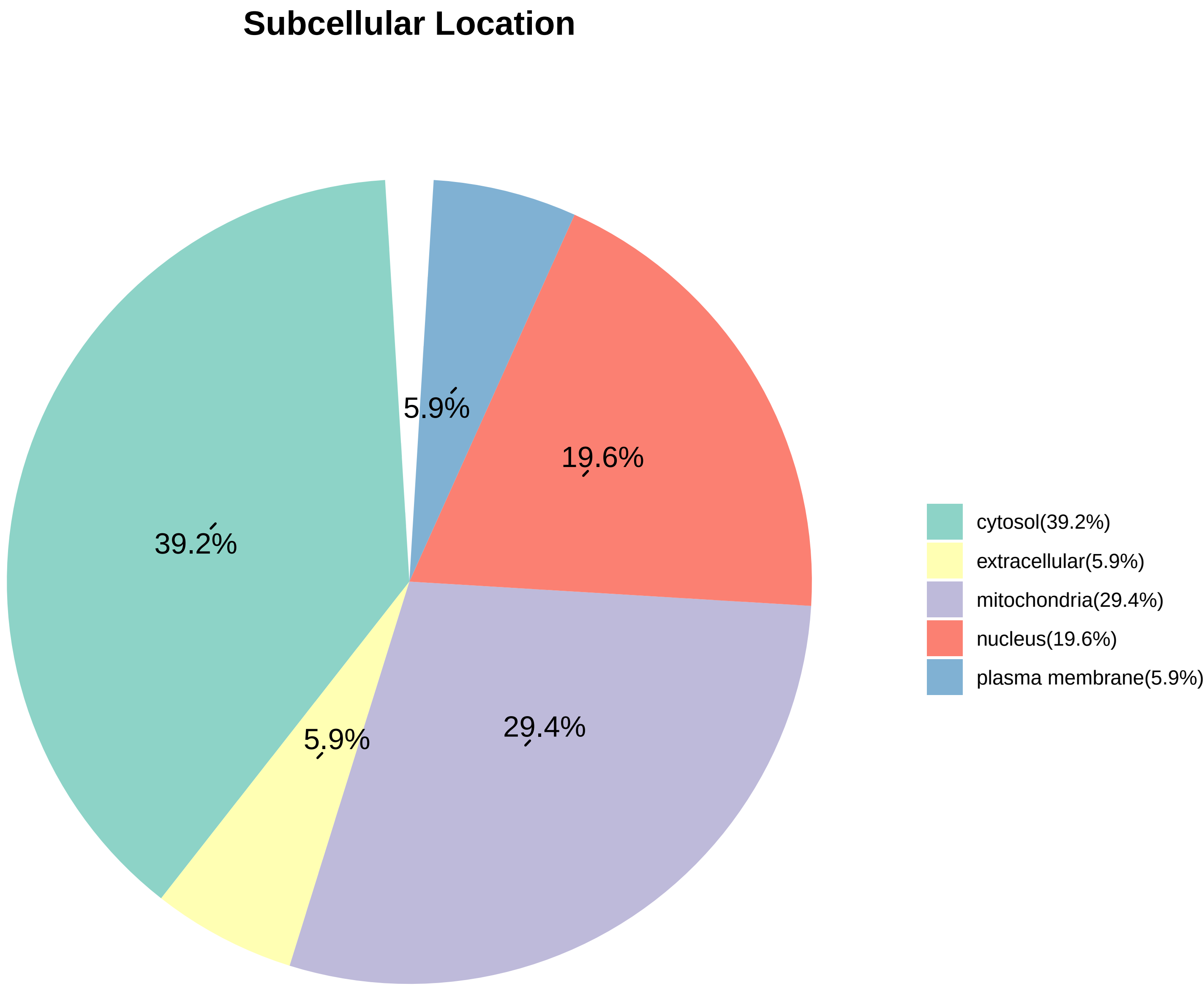
Subcellular Location
5.9%
19.6%
39.2%
5.9%
29.4%
cytosol(39.2%)
extracellular(5.9%)
mitochondria(29.4%)
nucleus(19.6%)
plasma membrane(5.9%)

Subcellular Location

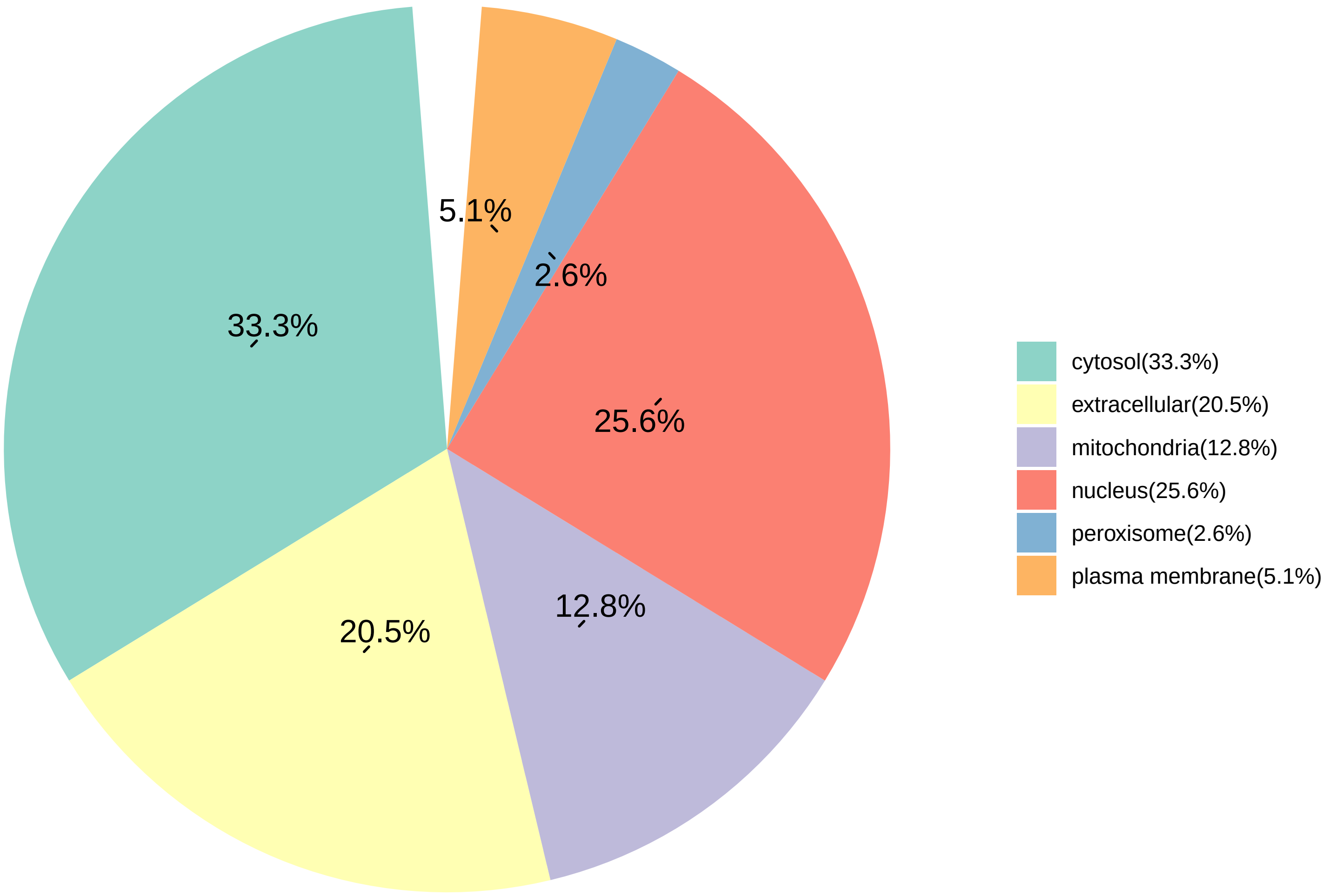

Supplement: SUPPLEMENTARY FIGURE 3 — Subcellular_classify. [file Image_3.pdf]
