# Supplementary material for: Integrative analysis of proteomics and lipidomic profiles reveal the fat deposition and meat quality in Duroc × Guangdong small spotted pig
Source: Front Vet Sci. 2024 Apr 10;11:1361441. doi: 10.3389/fvets.2024.1361441 (PMC11041638; doi:10.3389/fvets.2024.1361441)
Supplement: SUPPLEMENTARY FIGURE 5 — GO bar chart. [file Image_5.pdf]

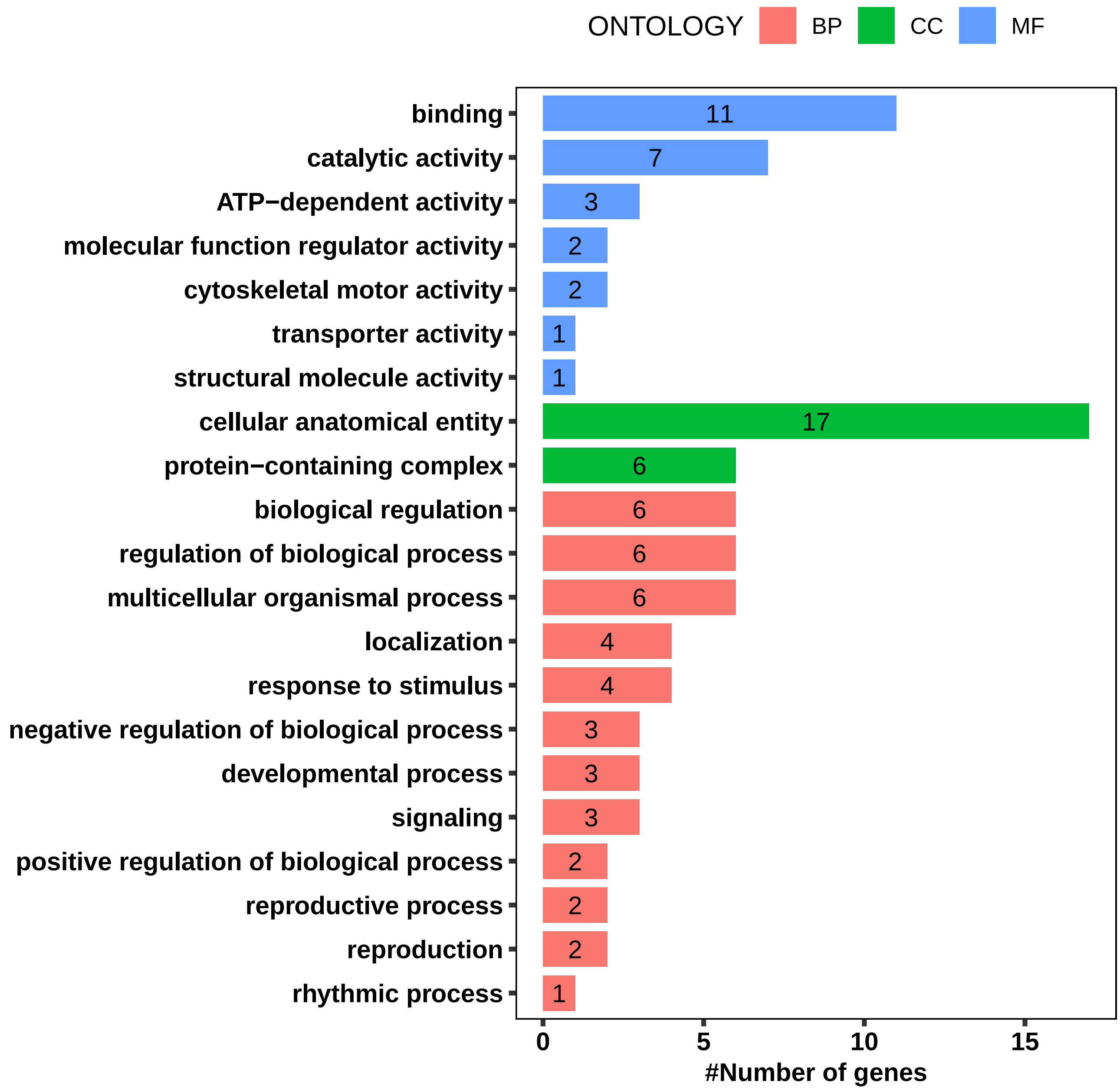
ONTOLOGY
BP
CC
MF
binding
catalytic activity
ATP−dependent activity
molecular function regulator activity
cytoskeletal motor activity
transporter activity
structural molecule activity
cellular anatomical entity
protein−containing complex
biological regulation
regulation of biological process
multicellular organismal process
localization
response to stimulus
negative regulation of biological process
developmental process
signaling
positive regulation of biological process
reproductive process
reproduction
rhythmic process
11
7
3
2
2
1
1
17
6
6
6
6
4
4
3
3
3
2
2
2
1
0
5
10
15
#Number of genes

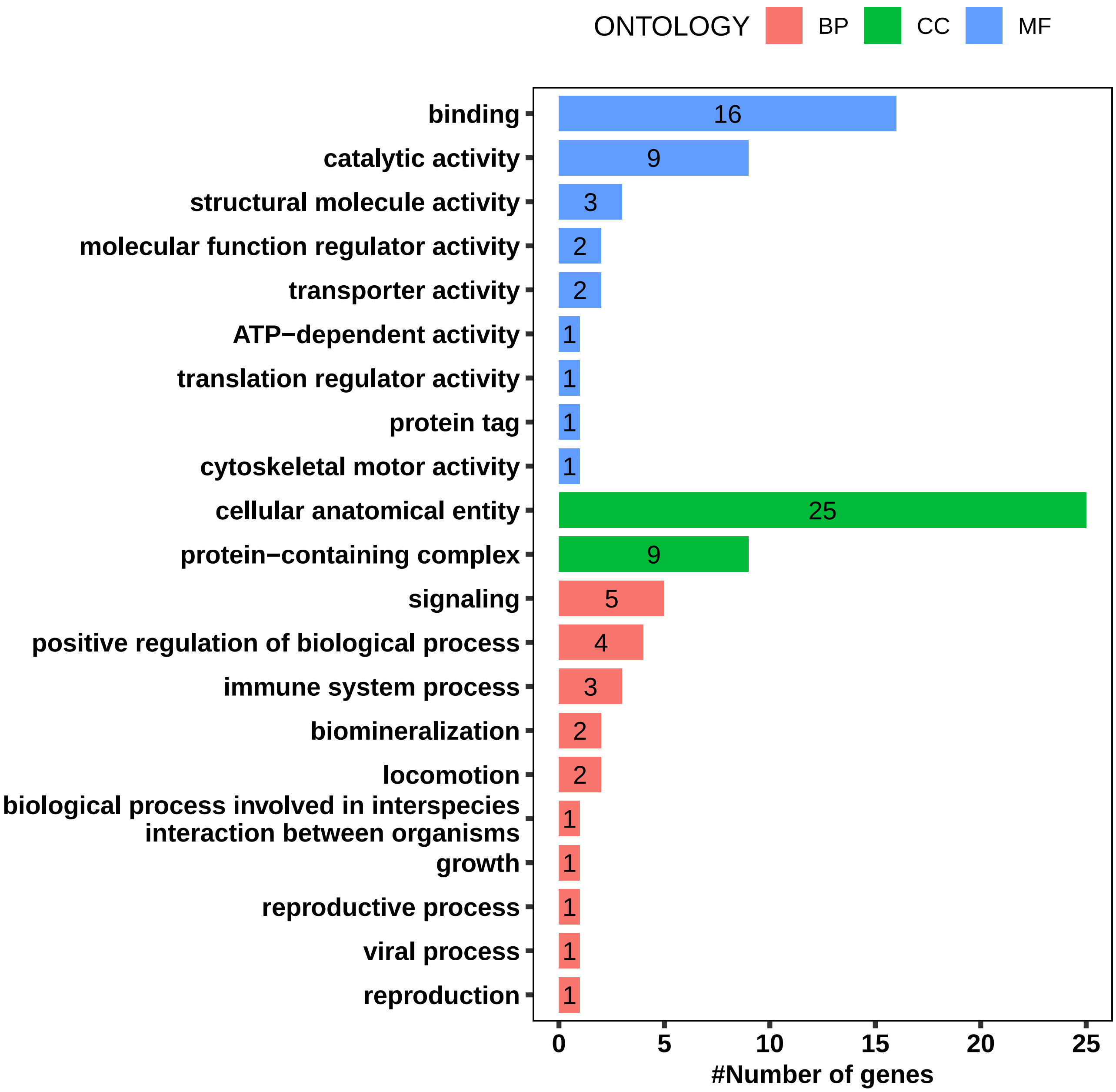
ONTOLOGY
BP
CC
MF
binding
catalytic activity
structural molecule activity
molecular function regulator activity
transporter activity
ATP−dependent activity
translation regulator activity
protein tag
cytoskeletal motor activity
cellular anatomical entity
protein−containing complex
signaling
positive regulation of biological process
immune system process
biomineralization
locomotion
biological process involved in interspecies interaction between organisms
growth
reproductive process
viral process
reproduction
16
9
3
2
2
1
1
1
1
25
9
5
4
3
2
2
1
1
1
1
1
0
5
10
15
20
25
#Number of genes

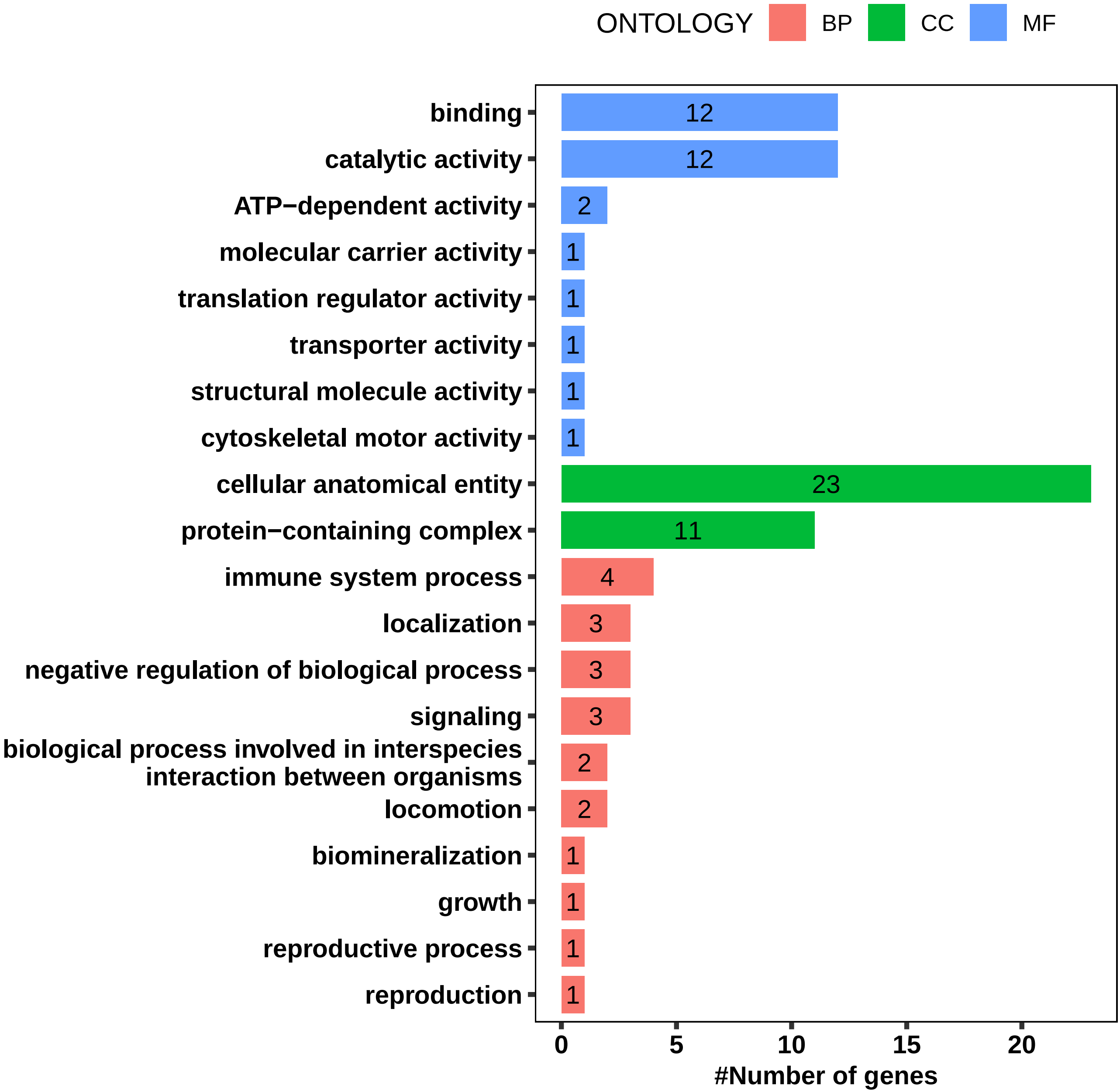
ONTOLOGY
BP
CC
MF
binding
catalytic activity
ATP−dependent activity
molecular carrier activity
translation regulator activity
transporter activity
structural molecule activity
cytoskeletal motor activity
cellular anatomical entity
protein−containing complex
immune system process
localization
negative regulation of biological process
signaling
biological process involved in interspecies interaction between organisms
locomotion
biomineralization
growth
reproductive process
reproduction
12
12
2
1
1
1
1
1
23
11
4
3
3
3
2
2
1
1
1
1
0
5
10
15
20
#Number of genes

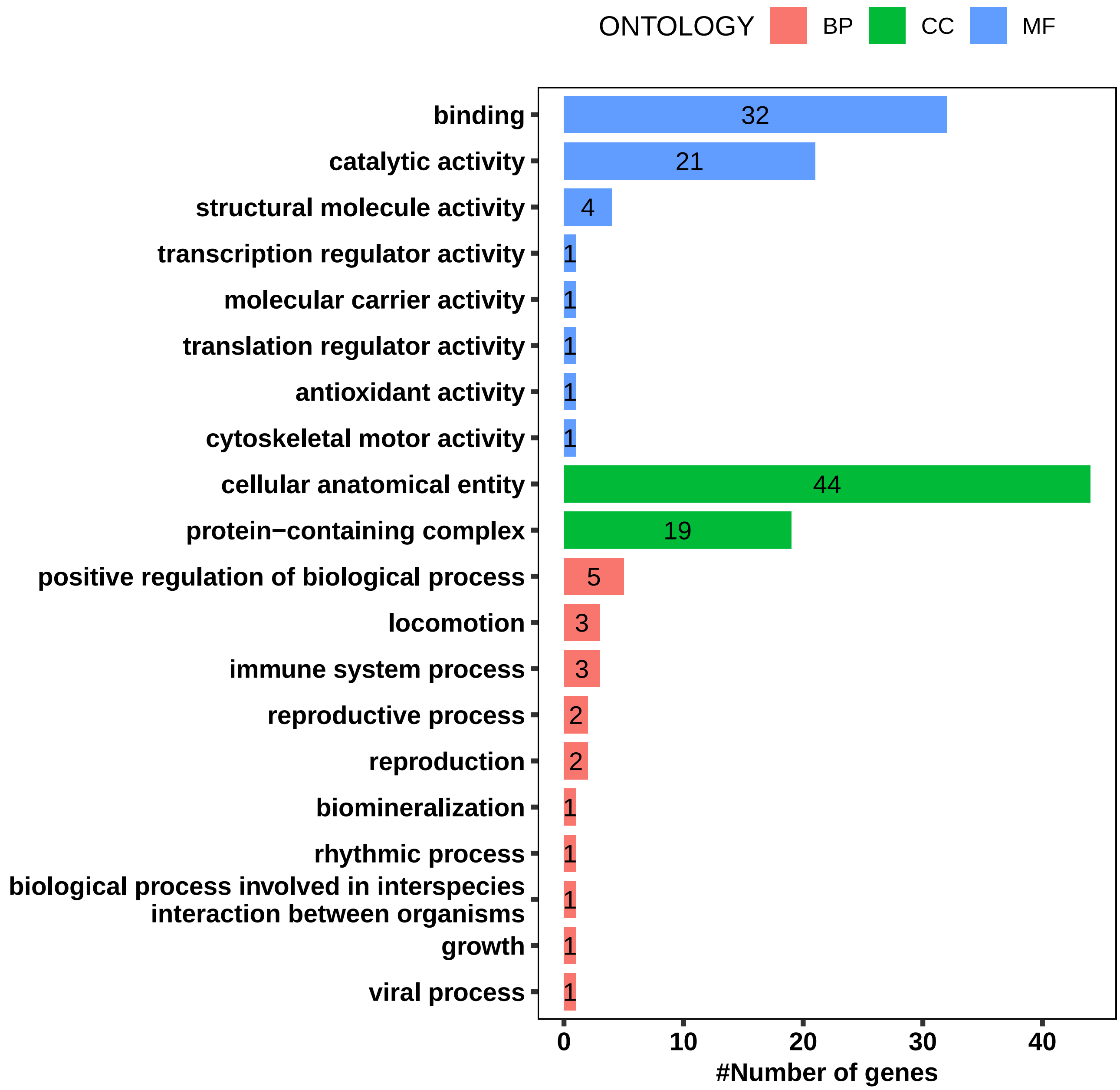

ONTOLOGY
BP
CC
MF
binding
catalytic activity
structural molecule activity
transcription regulator activity
molecular carrier activity
translation regulator activity
antioxidant activity
cytoskeletal motor activity
cellular anatomical entity
protein−containing complex
positive regulation of biological process
locomotion
immune system process
reproductive process
reproduction
biomineralization
rhythmic process
biological process involved in interspecies interaction between organisms
growth
viral process
32
21
4
1
1
1
1
1
44
19
5
3
3
2
2
1
1
1
1
1
0
10
20
30
40
#Number of genes

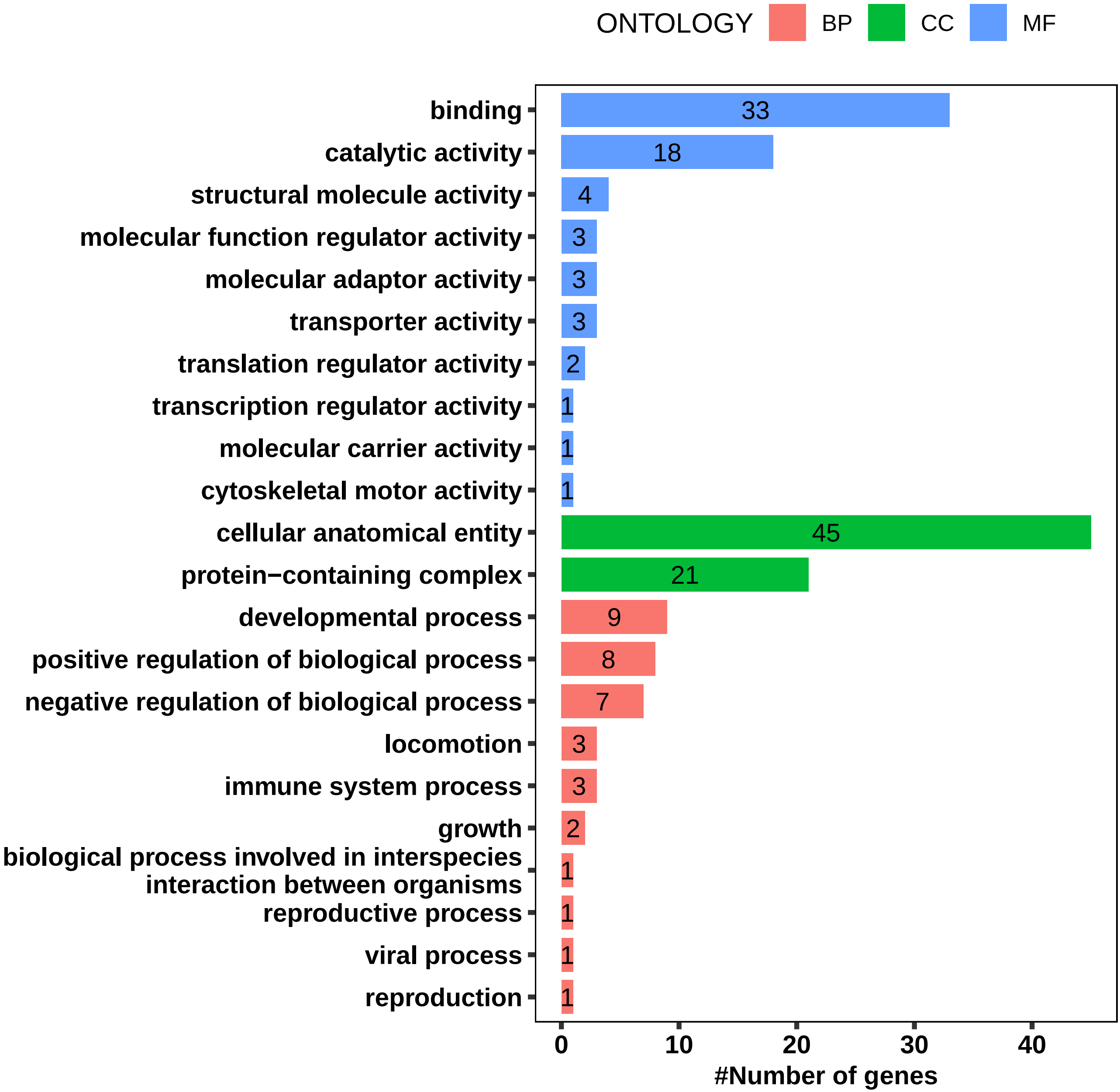

ONTOLOGY
BP
CC
MF
binding
catalytic activity
structural molecule activity
molecular function regulator activity
molecular adaptor activity
transporter activity
translation regulator activity
transcription regulator activity
molecular carrier activity
cytoskeletal motor activity
cellular anatomical entity
protein−containing complex
developmental process
positive regulation of biological process
negative regulation of biological process
locomotion
immune system process
growth
biological process involved in interspecies interaction between organisms
reproductive process
viral process
reproduction
33
18
4
3
3
3
2
1
1
1
45
21
9
8
7
3
3
2
1
1
1
1
0
10
20
30
40
#Number of genes

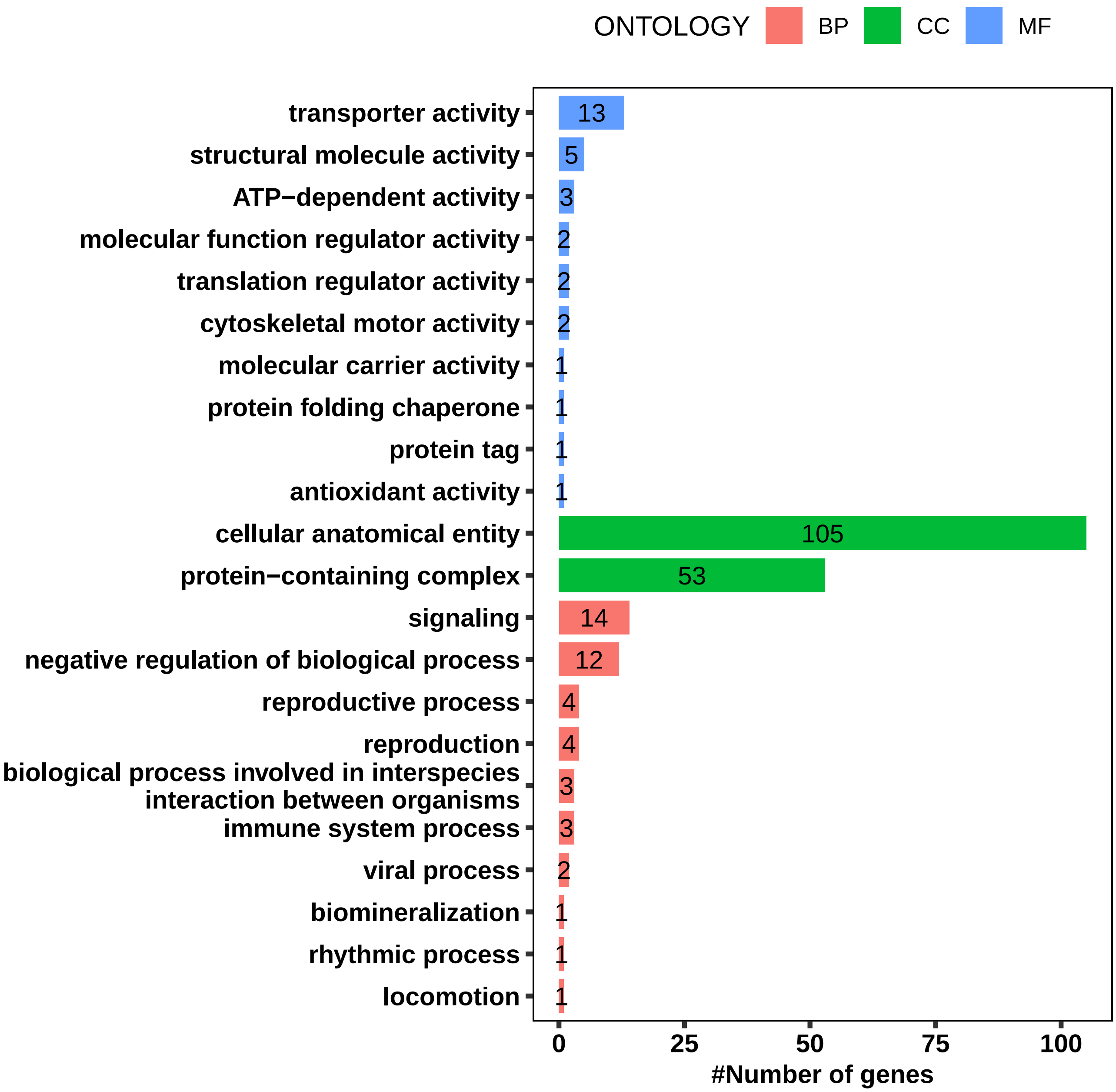

ONTOLOGY
BP
CC
MF
transporter activity
structural molecule activity
ATP−dependent activity
molecular function regulator activity
translation regulator activity
cytoskeletal motor activity
molecular carrier activity
protein folding chaperone
protein tag
antioxidant activity
cellular anatomical entity
protein−containing complex
signaling
negative regulation of biological process
reproductive process
reproduction
biological process involved in interspecies interaction between organisms
immune system process
viral process
biomineralization
rhythmic process
locomotion
13
5
3
2
2
2
1
1
1
1
105
53
14
12
4
4
3
3
2
1
1
1
0
25
50
75
100
#Number of genes

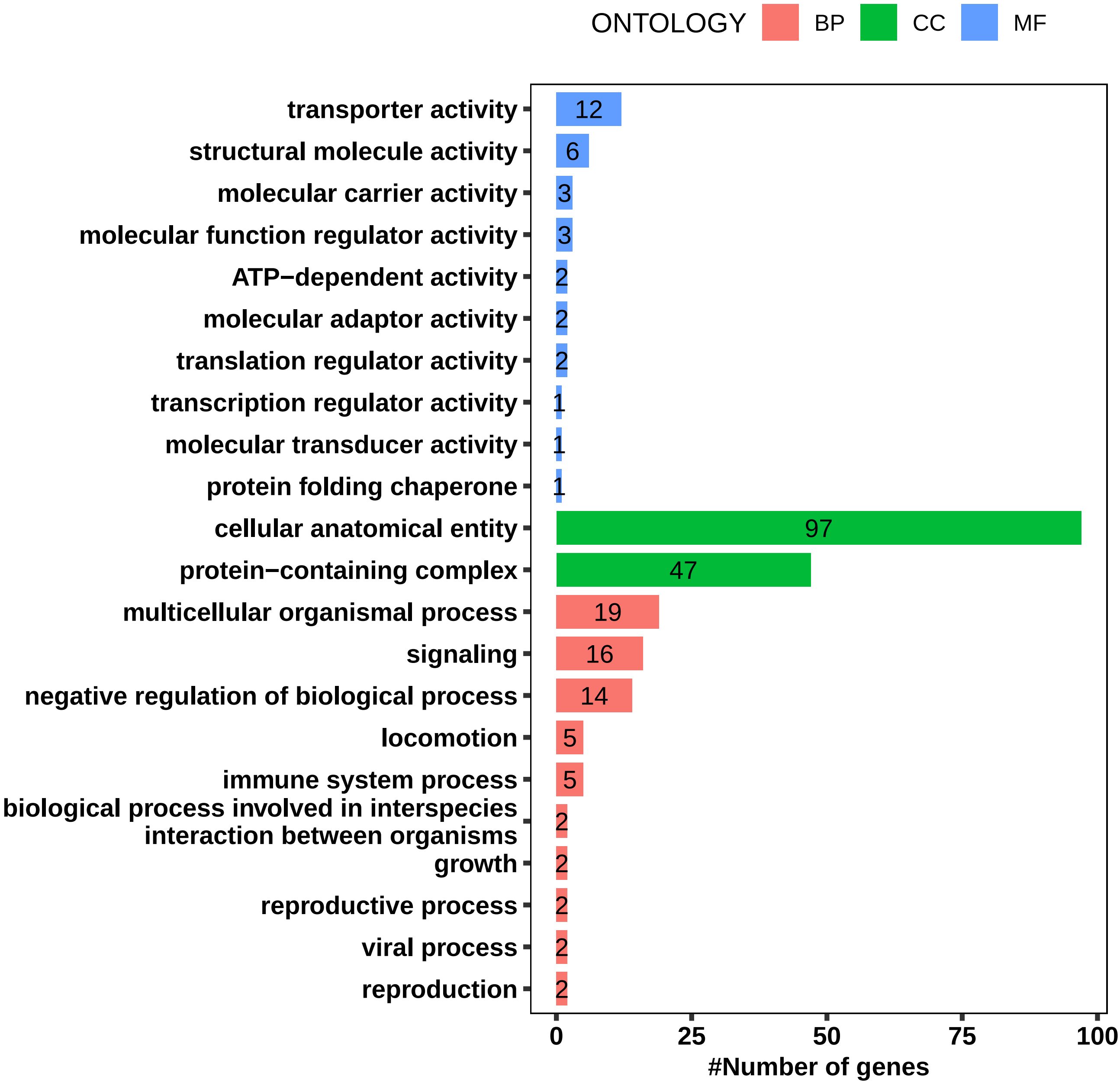
ONTOLOGY
BP
CC
MF
transporter activity
structural molecule activity
molecular carrier activity
molecular function regulator activity
ATP−dependent activity
molecular adaptor activity
translation regulator activity
transcription regulator activity
molecular transducer activity
protein folding chaperone
cellular anatomical entity
protein−containing complex
multicellular organismal process
signaling
negative regulation of biological process
locomotion
immune system process
biological process involved in interspecies interaction between organisms
growth
reproductive process
viral process
reproduction
12
6
3
3
2
2
2
1
1
1
97
47
19
16
14
5
5
2
2
2
2
2
0
25
50
75
100
#Number of genes

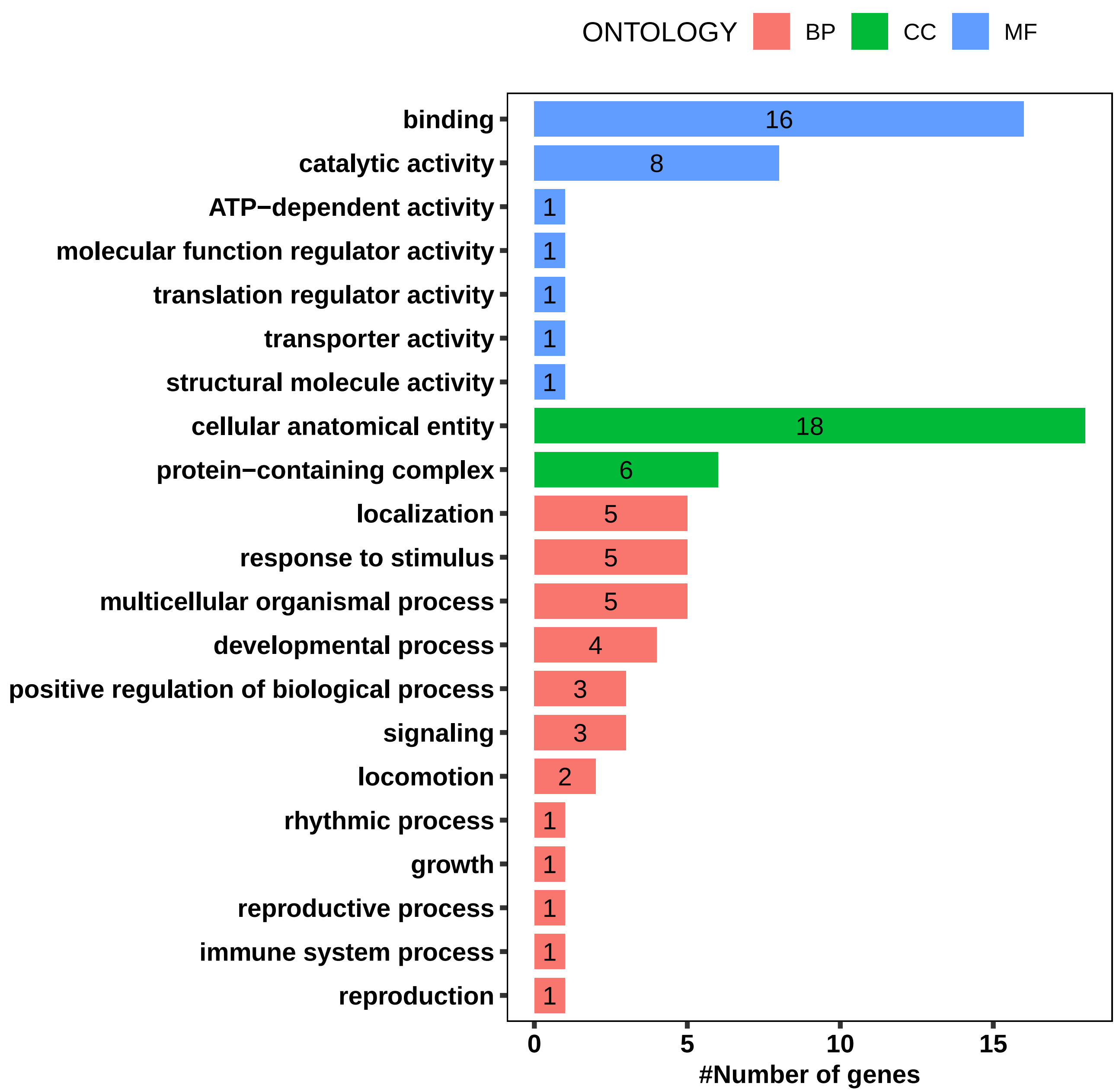
ONTOLOGY
BP
CC
MF
binding
catalytic activity
ATP−dependent activity
molecular function regulator activity
translation regulator activity
transporter activity
structural molecule activity
cellular anatomical entity
protein−containing complex
localization
response to stimulus
multicellular organismal process
developmental process
positive regulation of biological process
signaling
locomotion
rhythmic process
growth
reproductive process
immune system process
reproduction
16
8
1
1
1
1
1
18
6
5
5
5
4
3
3
2
1
1
1
1
1
0
5
10
15
#Number of genes

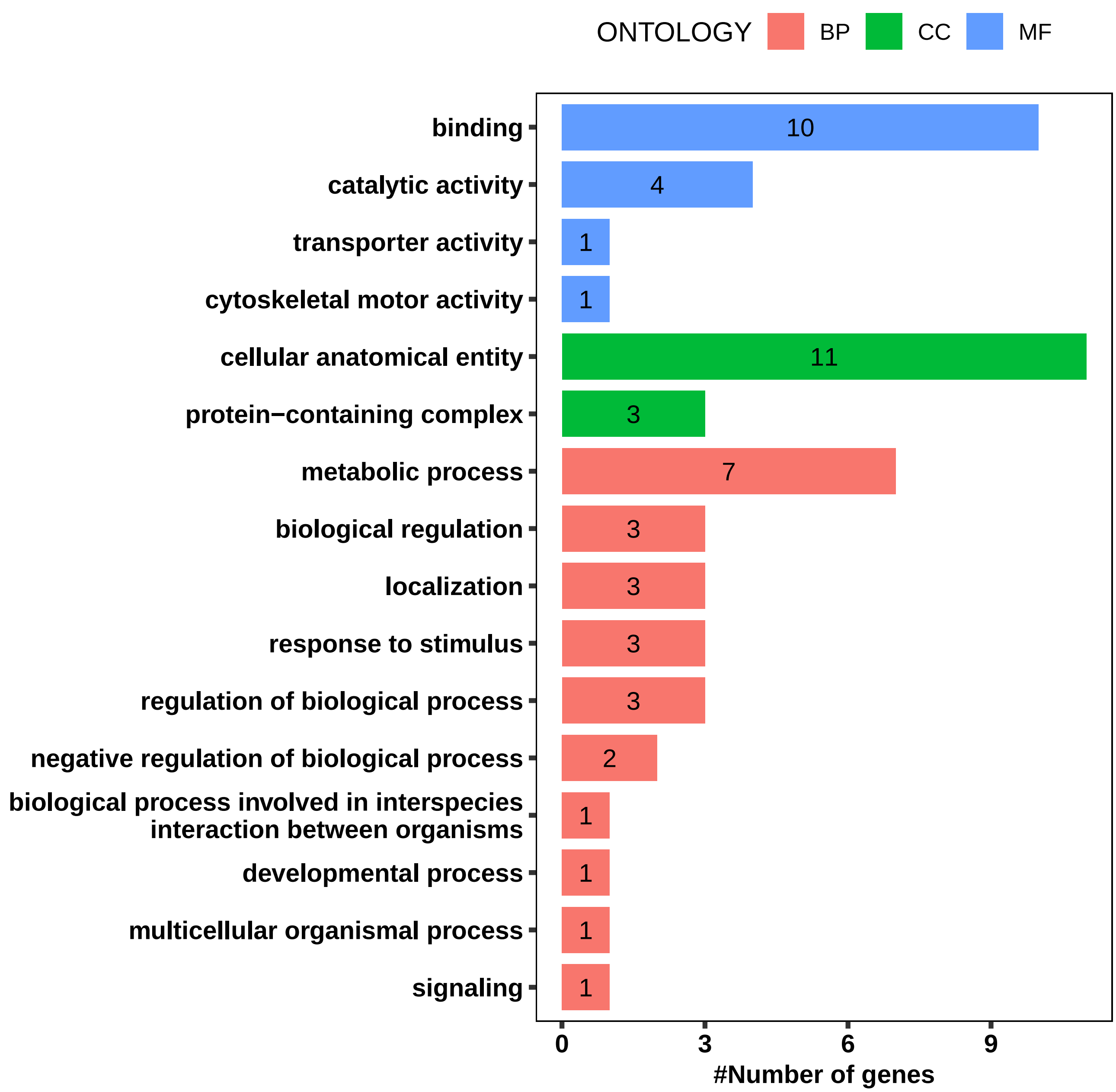
ONTOLOGY
BP
CC
MF
binding
catalytic activity
transporter activity
cytoskeletal motor activity
cellular anatomical entity
protein−containing complex
metabolic process
biological regulation
localization
response to stimulus
regulation of biological process
negative regulation of biological process
biological process involved in interspecies interaction between organisms
developmental process
multicellular organismal process
signaling
10
4
1
1
11
3
7
3
3
3
3
2
1
1
1
1
0
3
6
9
#Number of genes
