# Supplementary material for: Integrative analysis of proteomics and lipidomic profiles reveal the fat deposition and meat quality in Duroc × Guangdong small spotted pig
Source: Front Vet Sci. 2024 Apr 10;11:1361441. doi: 10.3389/fvets.2024.1361441 (PMC11041638; doi:10.3389/fvets.2024.1361441)
Supplement: SUPPLEMENTARY FIGURE 6 — KEGG pathway enrichment analysis. [file Image_6.pdf]

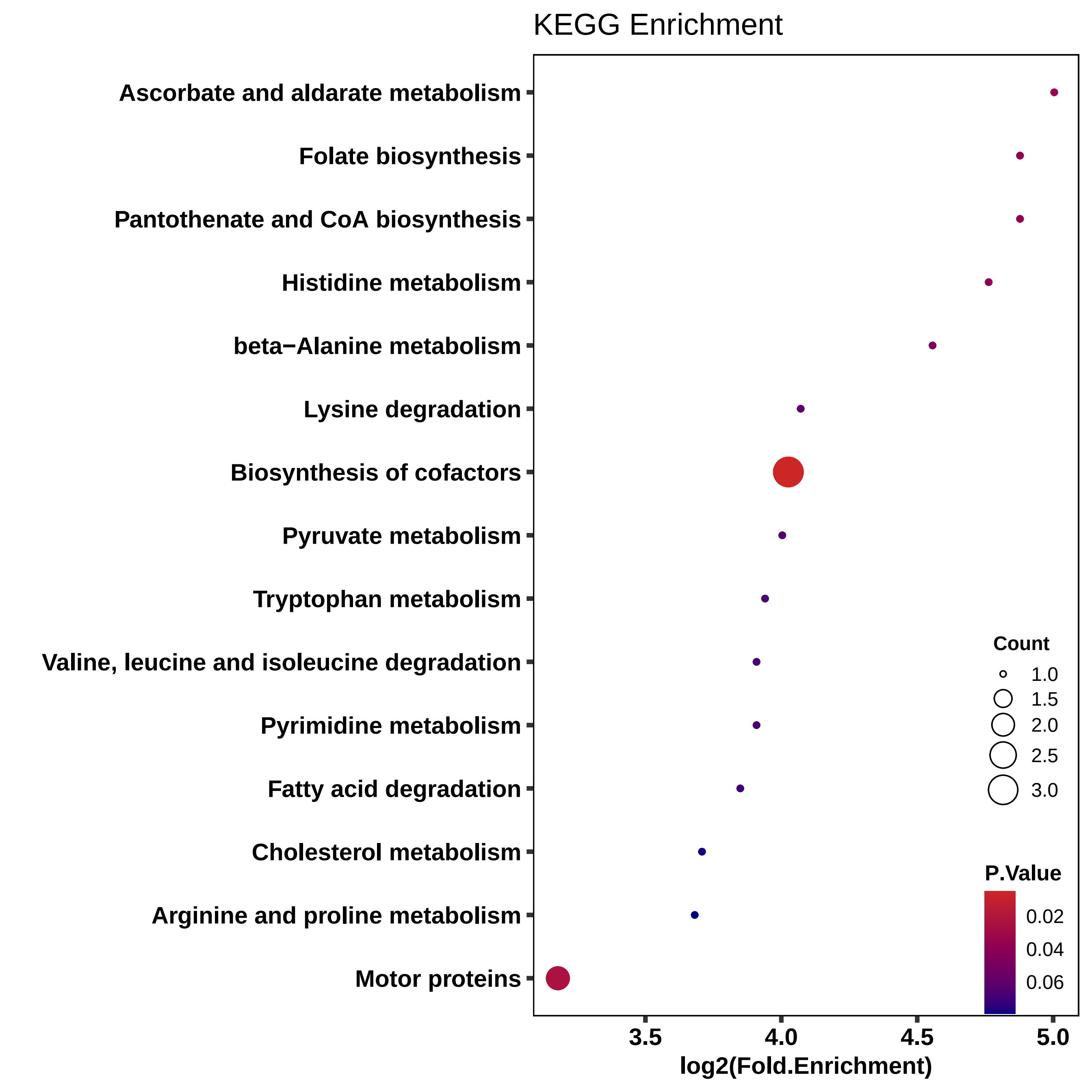
KEGG Enrichment
Ascorbate and aldarate metabolism
Folate biosynthesis
Pantothenate and CoA biosynthesis
Histidine metabolism
beta−Alanine metabolism
Lysine degradation
Biosynthesis of cofactors
Pyruvate metabolism
Tryptophan metabolism
Valine, leucine and isoleucine degradation
Pyrimidine metabolism
Fatty acid degradation
Cholesterol metabolism
Arginine and proline metabolism
Motor proteins
Count
1.0
1.5
2.0
2.5
3.0
P.Value
0.02
0.04
0.06
3.5
4.0
4.5
5.0
log2(Fold.Enrichment)

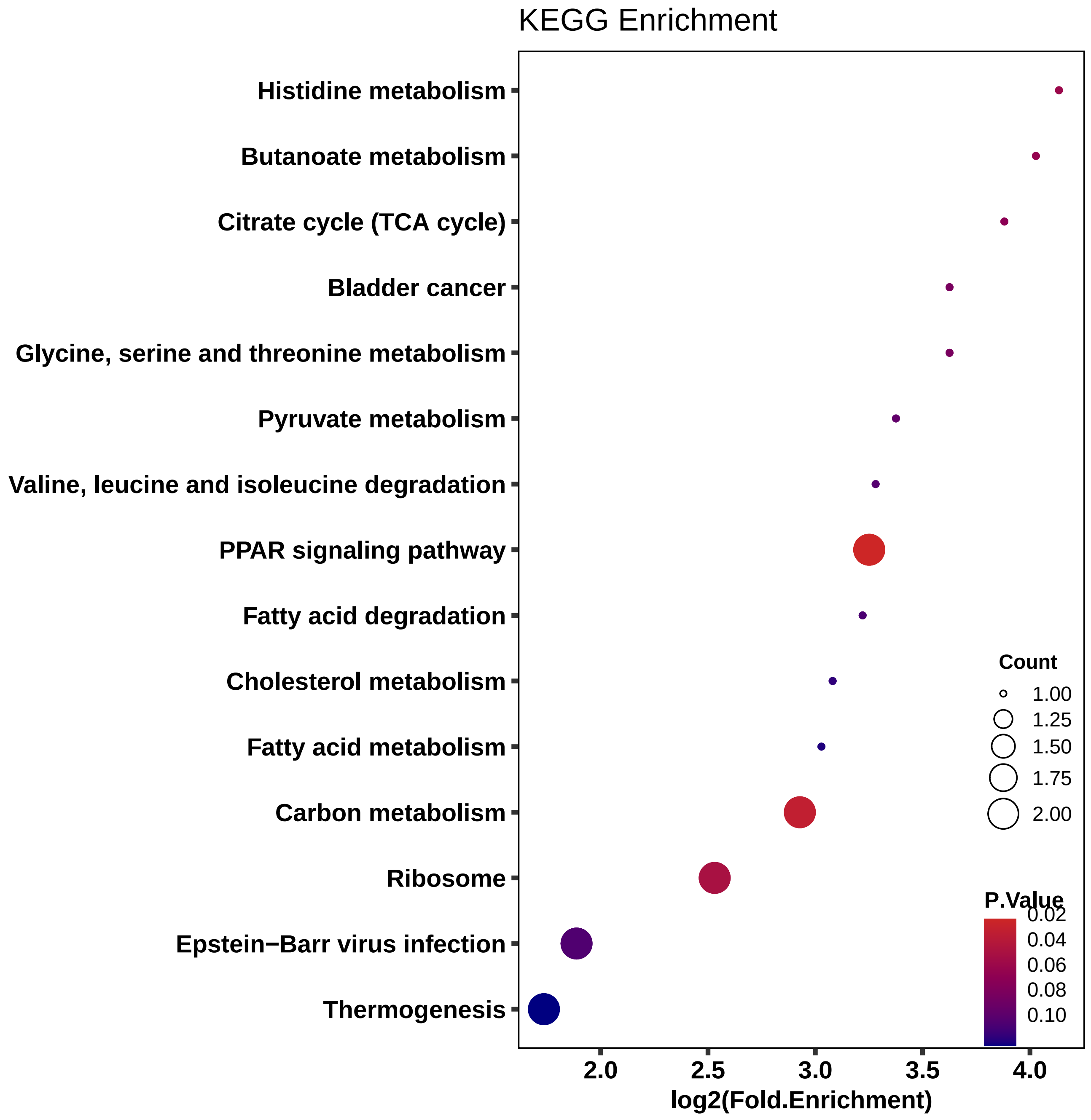
KEGG Enrichment
Histidine metabolism
Butanoate metabolism
Citrate cycle (TCA cycle)
Bladder cancer
Glycine, serine and threonine metabolism
Pyruvate metabolism
Valine, leucine and isoleucine degradation
PPAR signaling pathway
Fatty acid degradation
Cholesterol metabolism
Fatty acid metabolism
Carbon metabolism
Ribosome
Epstein−Barr virus infection
Thermogenesis
2.0
2.5
3.0
3.5
4.0
log2(Fold.Enrichment)
Count
1.00
1.25
1.50
1.75
2.00
P.Value
0.02
0.04
0.06
0.08
0.10

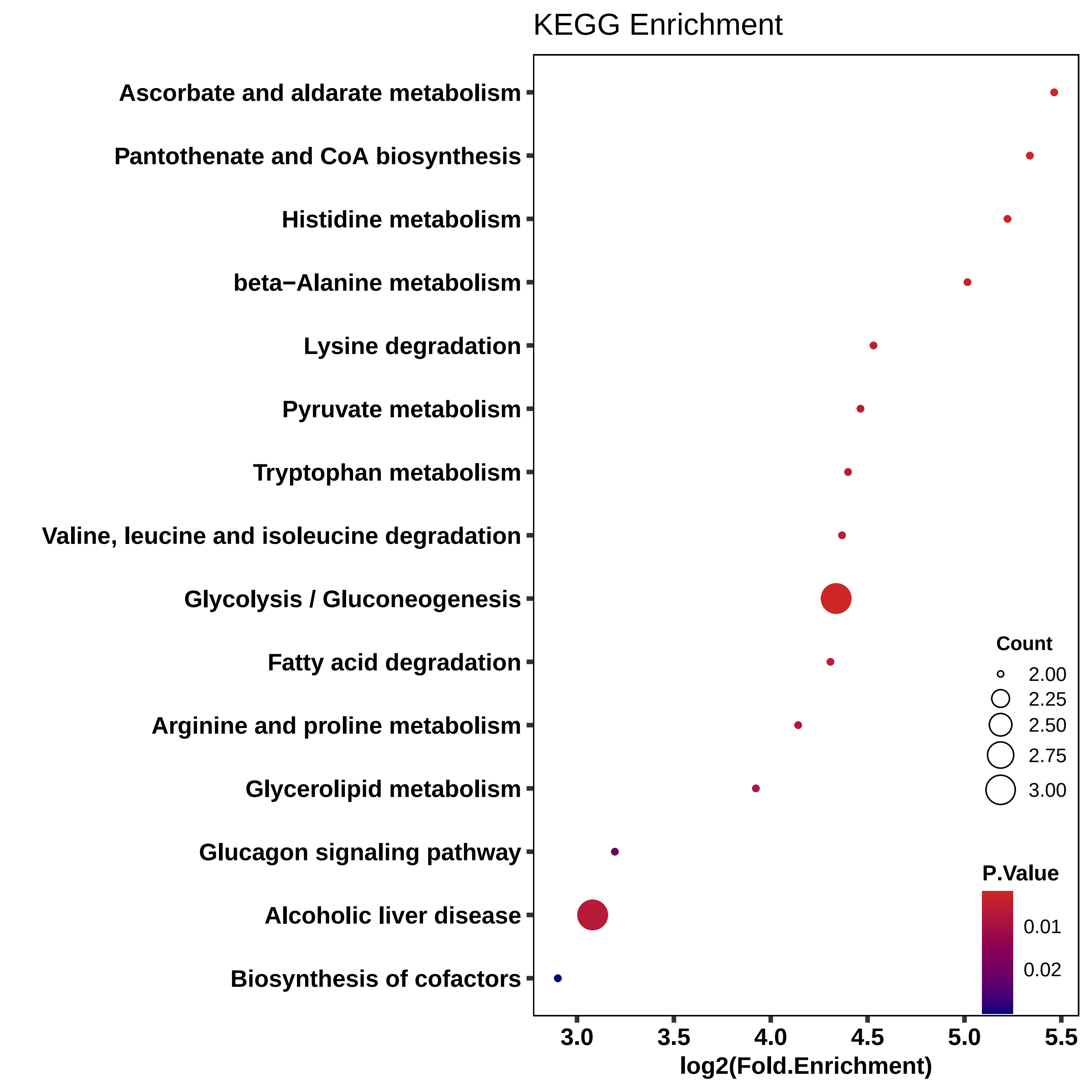
KEGG Enrichment
Ascorbate and aldarate metabolism
Pantothenate and CoA biosynthesis
Histidine metabolism
beta−Alanine metabolism
Lysine degradation
Pyruvate metabolism
Tryptophan metabolism
Valine, leucine and isoleucine degradation
Glycolysis / Gluconeogenesis
Fatty acid degradation
Arginine and proline metabolism
Glycerolipid metabolism
Glucagon signaling pathway
Alcoholic liver disease
Biosynthesis of cofactors
3.0
3.5
4.0
4.5
5.0
5.5
log2(Fold.Enrichment)
Count
2.00
2.25
2.50
2.75
3.00
P.Value
0.01
0.02

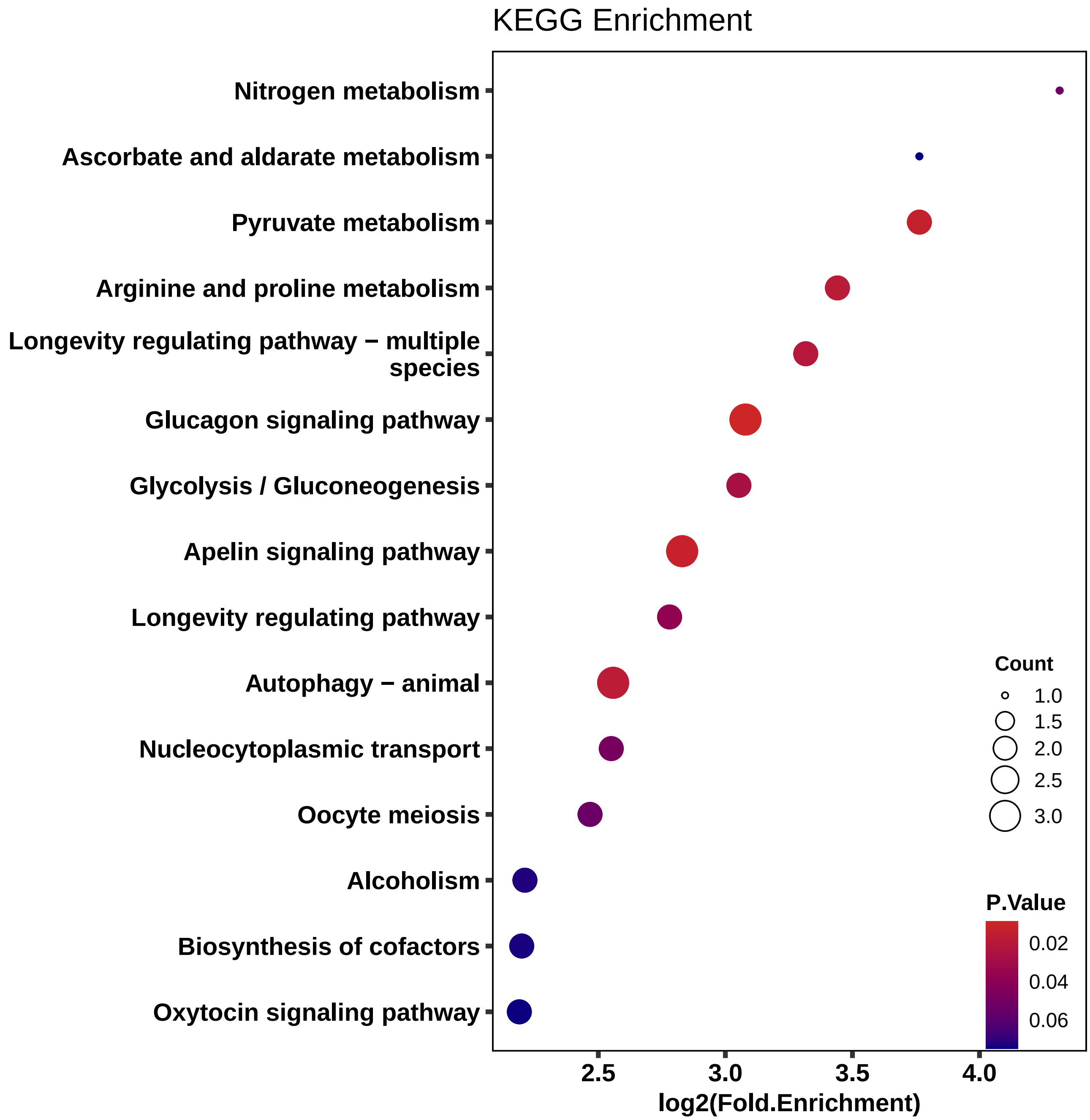

KEGG Enrichment
Nitrogen metabolism
Ascorbate and aldarate metabolism
Pyruvate metabolism
Arginine and proline metabolism
Longevity regulating pathway − multiple species
Glucagon signaling pathway
Glycolysis / Gluconeogenesis
Apelin signaling pathway
Longevity regulating pathway
Autophagy − animal
Nucleocytoplasmic transport
Oocyte meiosis
Alcoholism
Biosynthesis of cofactors
Oxytocin signaling pathway
2.5
3.0
3.5
4.0
log2(Fold.Enrichment)
Count
1.0
1.5
2.0
2.5
3.0
P.Value
0.02
0.04
0.06

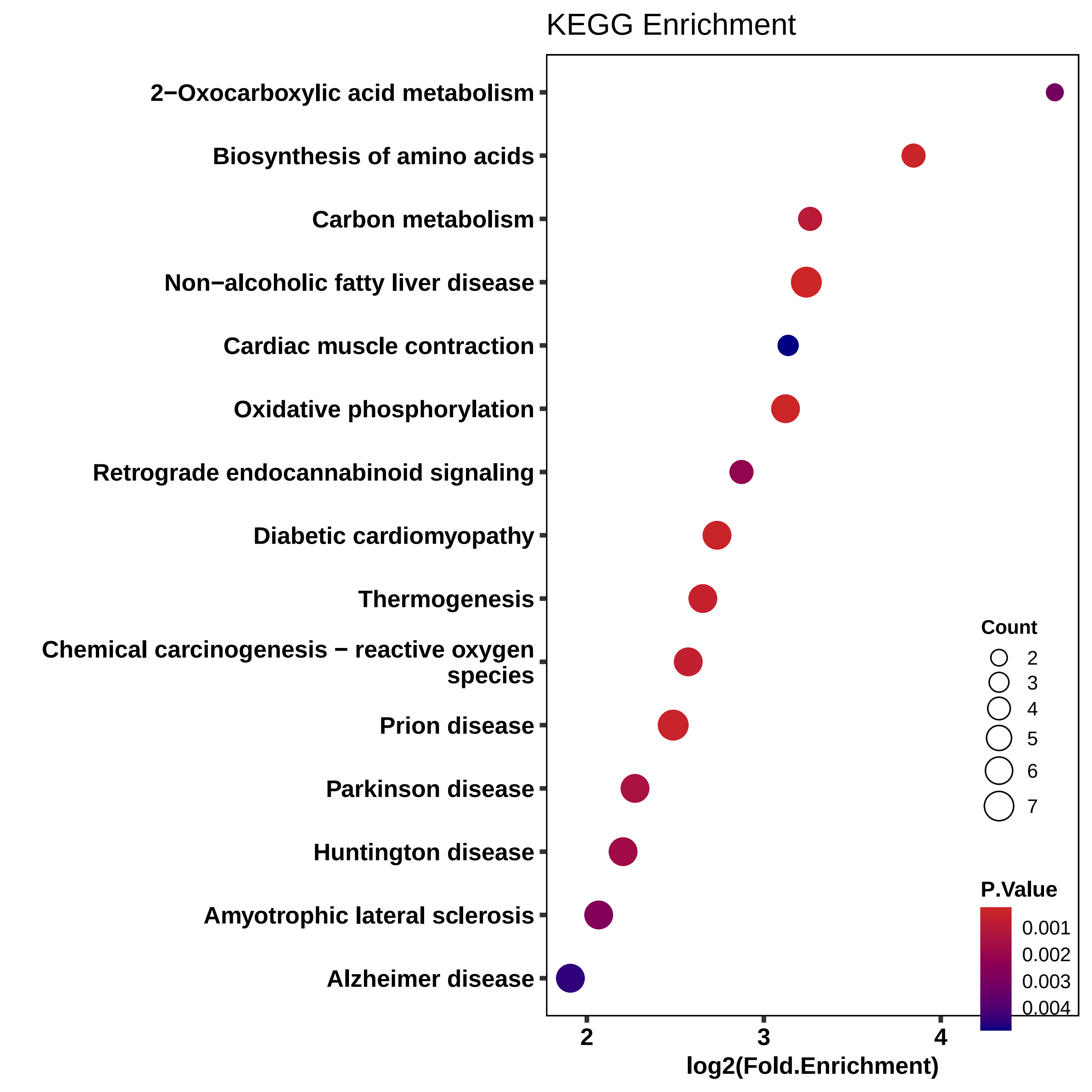

KEGG Enrichment
2−Oxocarboxylic acid metabolism
Biosynthesis of amino acids
Carbon metabolism
Non−alcoholic fatty liver disease
Cardiac muscle contraction
Oxidative phosphorylation
Retrograde endocannabinoid signaling
Diabetic cardiomyopathy
Thermogenesis
Chemical carcinogenesis − reactive oxygen species
Prion disease
Parkinson disease
Huntington disease
Amyotrophic lateral sclerosis
Alzheimer disease
2
3
4
log2(Fold.Enrichment)
Count
2
3
4
5
6
7
P.Value
0.001
0.002
0.003
0.004

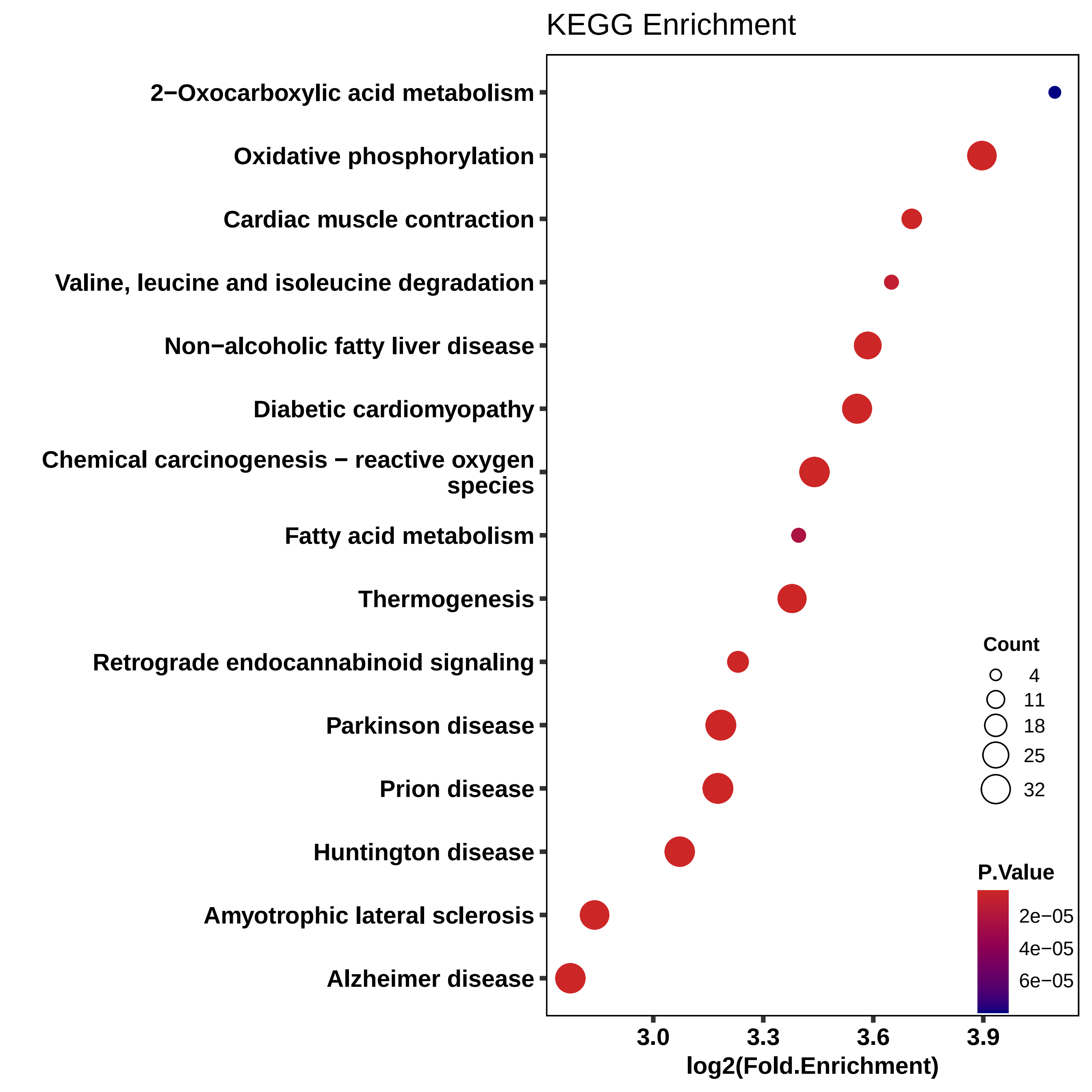

KEGG Enrichment
2−Oxocarboxylic acid metabolism
Oxidative phosphorylation
Cardiac muscle contraction
Valine, leucine and isoleucine degradation
Non−alcoholic fatty liver disease
Diabetic cardiomyopathy
Chemical carcinogenesis − reactive oxygen species
Fatty acid metabolism
Thermogenesis
Retrograde endocannabinoid signaling
Parkinson disease
Prion disease
Huntington disease
Amyotrophic lateral sclerosis
Alzheimer disease
Count
4
11
18
25
32
P.Value
2e−05
4e−05
6e−05
3.0
3.3
3.6
3.9
log2(Fold.Enrichment)

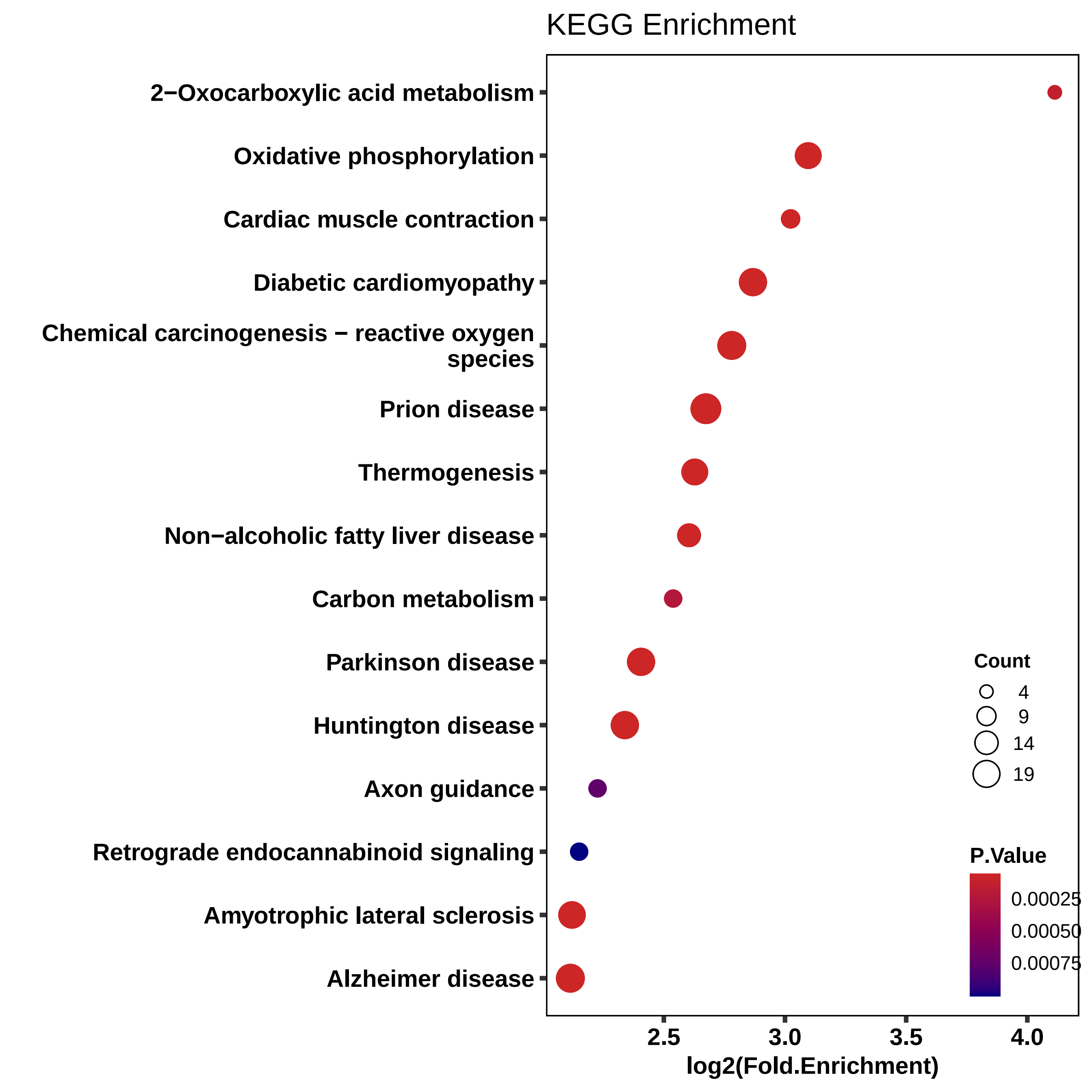

KEGG Enrichment
2−Oxocarboxylic acid metabolism
Oxidative phosphorylation
Cardiac muscle contraction
Diabetic cardiomyopathy
Chemical carcinogenesis − reactive oxygen species
Prion disease
Thermogenesis
Non−alcoholic fatty liver disease
Carbon metabolism
Parkinson disease
Huntington disease
Axon guidance
Retrograde endocannabinoid signaling
Amyotrophic lateral sclerosis
Alzheimer disease
Count
4
9
14
19
P.Value
0.00025
0.00050
0.00075
2.5
3.0
3.5
4.0
log2(Fold.Enrichment)

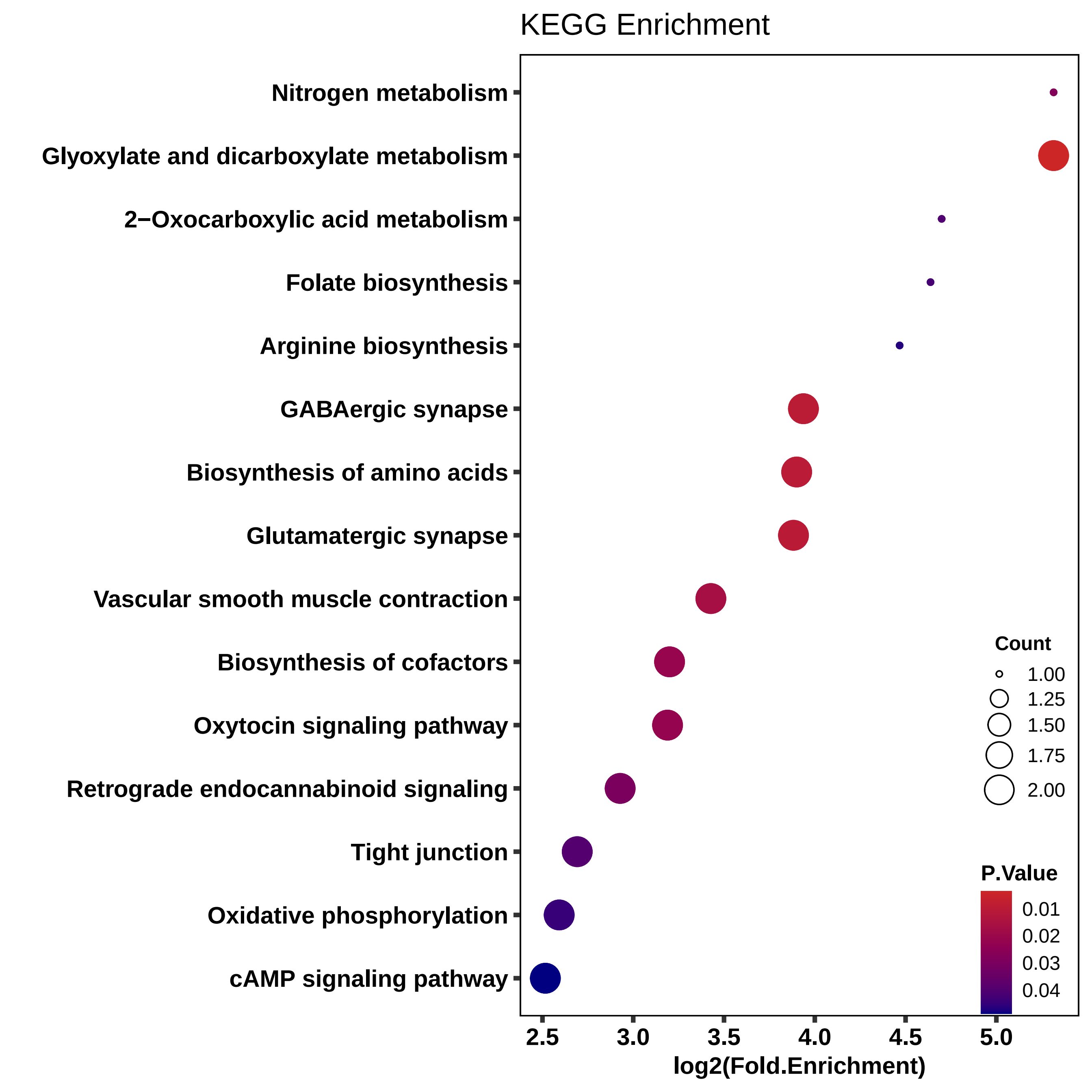
KEGG Enrichment
Nitrogen metabolism
Glyoxylate and dicarboxylate metabolism
2−Oxocarboxylic acid metabolism
Folate biosynthesis
Arginine biosynthesis
GABAergic synapse
Biosynthesis of amino acids
Glutamatergic synapse
Vascular smooth muscle contraction
Biosynthesis of cofactors
Oxytocin signaling pathway
Retrograde endocannabinoid signaling
Tight junction
Oxidative phosphorylation
cAMP signaling pathway
2.5
3.0
3.5
4.0
4.5
5.0
log2(Fold.Enrichment)
Count
1.00
1.25
1.50
1.75
2.00
P.Value
0.01
0.02
0.03
0.04

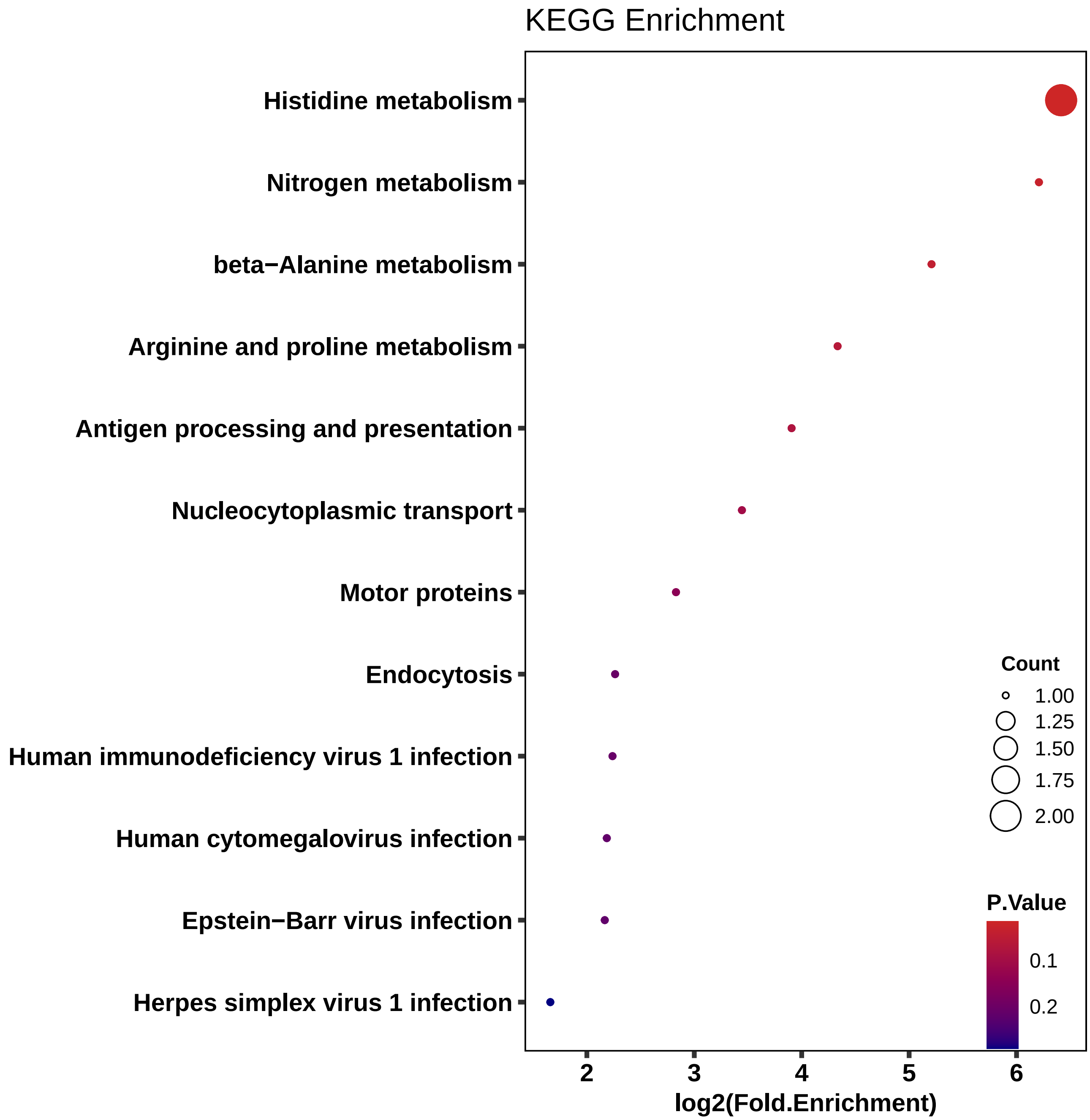
KEGG Enrichment
Histidine metabolism
Nitrogen metabolism
beta−Alanine metabolism
Arginine and proline metabolism
Antigen processing and presentation
Nucleocytoplasmic transport
Motor proteins
Endocytosis
Human immunodeficiency virus 1 infection
Human cytomegalovirus infection
Epstein−Barr virus infection
Herpes simplex virus 1 infection
Count
1.00
1.25
1.50
1.75
2.00
P.Value
0.1
0.2
2
3
4
5
6
log2(Fold.Enrichment)
